# Cluster Anion Substitution Tunes Flexibility and Porosity of MIL-53(Sc)-fum Conformational Isomers Isolated by Solvent-Induced MOF-to-MOF Self-Assembly

**DOI:** 10.1021/jacs.6c06041

**Published:** 2026-07-28

**Authors:** Catherine A. Walshe, Zachary H. Davis, Chi C. Hong, Thach N. Tu, Sanliang Ling, Claire Wilson, Joshua J. B. Levinsky, Claire L. Hobday, Sharon E. Ashbrook, Ross S. Forgan

**Affiliations:** † School of Chemistry, 3526 University of Glasgow, Joseph Black Building, University Avenue, Glasgow G12 8QQ, U.K.; ‡ EaStCHEM School of Chemistry and Centre of Magnetic Resonance, 7486 University of St Andrews, St Andrews KY16 9ST, U.K.; § EaStCHEM School of Chemistry and Centre for Science at Extreme Conditions, University of Edinburgh, King’s Buildings, David Brewster Road, Edinburgh EH9 3FJ, U.K.; ∥ Advanced Materials Research Group, Faculty of Engineering, 6123 University of Nottingham, University Park, Nottingham NG7 2RD, U.K.

## Abstract

Metal–organic frameworks (MOFs) of the type MIL-53­(M)-fum, where M^3+^ cations are connected by fumarate (fum) linkers in the canonical MIL-53 topology, have been investigated as possible adsorbents due to their structural rigidity, high porosity, and biologically endogenous organic linker. Examples reported to date are limited to those comprised of *p*-block metal ions (e.g., Al^3+^, Ga^3+^, and In^3+^); *d*-block metal ions (e.g., Fe^3+^ and Sc^3+^) tend to form MIL-88A­(M) materials with the fumarate linker. Herein, we describe the solvent-induced self-assembly of MIL-53­(Sc)-fum, by immersing MIL-88A­(Sc) in water or aqueous solutions of *N*,*N*-dimethylformamide (DMF). Single-crystal X-ray diffraction confirms the solvent-selective formation of two different conformational isomers of MIL-53­(Sc)-fum in this unusual MOF-to-MOF synthesis, and both are found to exhibit structural breathing, in contrast to the predominantly rigid *p*-block metal homologues. Furthermore, we show that the extent of flexibility can be tuned by postsynthetic cluster anion substitution. Refluxing MIL-53­(Sc)-fum samples in methanol results in 40–50% exchange of μ_2_–OH units for μ_2_-OCH_3_ groups, where the steric bulk holds the MOFs in a slightly more open pore configuration, enabling significant N_2_ adsorption at low pressures in contrast to the fully closed, unfunctionalized precursors. This study provides significant insight into MOF-to-MOF self-assembly, conformational isomerism in MOFs, and the effect of both metal ion and cluster anion substitution on structural flexibility, enabling careful tuning of highly porous MOFs from simple components.

## Graphical abstract

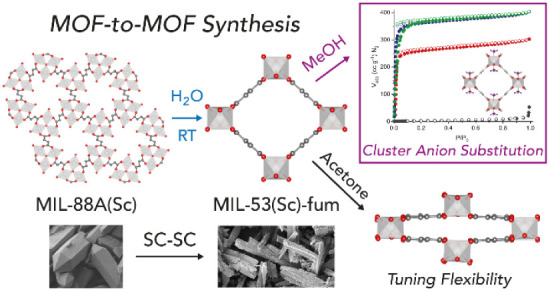

## Introduction

Metal–organic frameworks (MOFs) are coordination networks wherein metal ions or clusters are connected by organic linkers into multidimensional structures.^1^ MOFs are typically synthesized by combining an appropriate metal source (usually a simple metal salt such as an acetate, nitrate or halide) with a linker (or linkers) and providing an energy input, be that direct or microwave heating (usually in an appropriate solvent), sonication, mechanochemical grinding, etc.^2^ Alternatively, novel MOFs can be synthesized from extant MOFs. The most common route is through postsynthetic modification,^3^ whereby new functionality is added by forming coordinative or covalent bonds at different sites on the MOF, including the organic ligand,^4^ the metals^5^ and/or attendant anions^6^ of the inorganic secondary building unit (SBU), and at bound modulator molecules.^7^ Postsynthetic exchange^8^ and/or insertion of both metals^9^ and ligands^10^ is also possible, while external physical stimuli such as light^11^ or heat^12^ can also be used to modify network topology and/or interpenetration.

Much rarer is the deliberate topological phase transformation of one MOF into another while retaining the same metals and ligands.^13^ Many metal–ligand combinations occupy a complex phase space, where numerous competing MOF structures can form depending on synthetic conditions.^14^ A prime example is that of trivalent metal cations with linear dicarboxylate linkers. Using benzene-1,4-dicarboxylate (BDC) and Sc^3+^ as an example, there are at least five possible phases with three distinct inorganic SBUs: MIL-88B­(Sc)^15^ and MIL-101­(Sc)^16^ are polymorphs which share the discrete [Sc_3_O­(RCO_2_)_6_(solvent)_2_(monoanion)] SBU (Figure 1a), with typical formula [Sc_3_O­(BDC)_3_(OH)­(OH_2_)_2_]; MIL-53­(Sc)^16,17^ and MIL-68­(Sc)^18^ are a second pair of polymorphs with formula [Sc­(OH)­(BDC)] which share the infinite one-dimensional [Sc­(μ_2_–OH)­(RCO_2_)_2_]_*n*_ chain SBU (Figure 1b); while Sc_2_BDC_3_ contains an infinite one-dimensional [Sc­(RCO_2_)_3_]_*n*_ chain SBU (Figure 1c).^19^ Other trivalent metals such as Fe^3+^ give rise to further possible MOFs with BDC and its derivatives.^20^ As such, there is significant interest in (i) controlling the synthetic selection of a single phase-pure material, typically using modulated self-assembly,^21^ and (ii) establishing the thermodynamic landscape that dictates the formation of different phases.^22^ It is generally accepted across the trivalent metals that the relative thermodynamic stabilities of these phases can be ordered as MIL-101­(M) < MIL-88B­(M) < MIL-68­(M) < MIL-53­(M).^20,23^ The Sc_2_BDC_3_ phase has so far only been obtained for Sc^3+^ and so its place in this series is not conclusively established; the high temperatures required for synthesis suggest it may be the thermodynamic product.^19,24^ There have been reports of the conversion of individual phases in this system to more thermodynamically stable ones. For example, both MIL-101­(V) and MIL-88B­(V) have been converted to MIL-47 (the vanadium homologue of MIL-53) by heating in ethanol at 200 °C.^23^ Extended isoreticular analogues of MIL-68­(Al) and MIL-53­(Al) have also been reported to interconvert during solvation and desolvation.^25^ This would suggest that, given sufficient kinetic lability around the trivalent metal cation,^26^ MOF-to-MOF synthesis is feasible in these complex phase landscapes. Indeed, *in situ* experiments^27^ have shown the formation of intermediate kinetic phases during the early stages of the syntheses of MIL-53­(Fe)^28^ and MIL-53­(Al)_NH_2_.^29^

**Figure 1:**
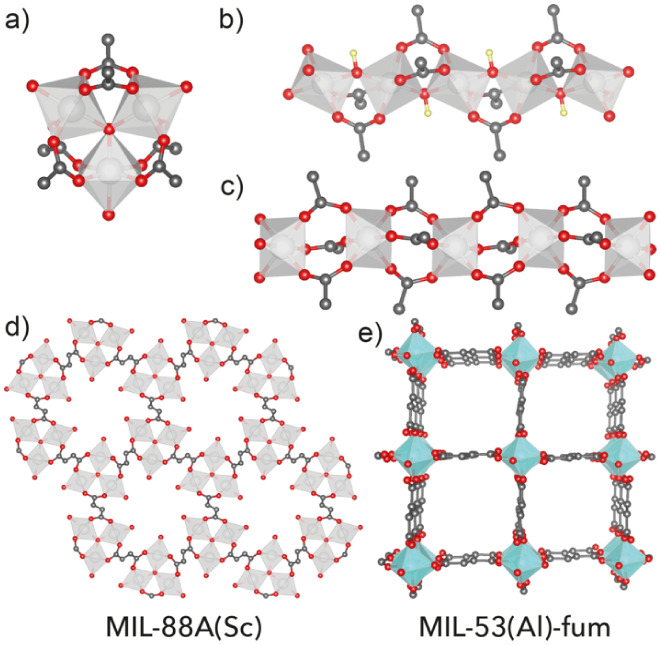
Depictions of common SBUs found in Sc MOFs, including a) the [Sc_3_O­(RCO_2_)_6_(OH)­(OH_2_)_2_] SBU, b) a portion of the [Sc­(μ_2_–OH)­(RCO_2_)_2_]*_n_* chain SBU, and c) a portion of the [Sc­(RCO_2_)_3_]*_n_* chain SBU. Portions of crystal structures of fumarate MOFs of trivalent metals, d) MIL-88A­(Sc), which contains the trimeric SBU depicted in part a), and e) MIL-53­(Al)-fum, which contains the chain SBU depicted in part b). Sc: light gray polyhedra; Al: pale blue polyhedra; C: dark gray; O: red; H: yellow. H atoms removed for clarity other than μ_2_–OH ligands in part b).

The fumarate (fum) linker has frequently been used to prepare isoreticular derivatives of these BDC-based MOFs with trivalent metals, given that its low molecular mass enhances gravimetric gas uptakes^30^ and, being an endogeneous component of the Krebs cycle,^31^ its resultant MOFs often display excellent biocompatibility.^32^ Known fumarate MOFs of trivalent metals include: MIL-88A­(M) (M = Sc^3+^, Fe^3+^),^33^ a flexible MOF containing the trimeric [M_3_O] SBU which is isoreticular to MIL-88B (Figure 1d); and MIL-53­(M)-fum (M = Al^3+^, Ga^3+^, In^3+^),^34–36^ an isoreticular analogue of MIL-53 whose varying metal homologues are predominantly rigid and display permanent porosity (Figure 1e). We have recently described the three possible conformational isomers that MIL-53­(M)-fum can adopt, based on the relative orientations of the different fumarate linkers, with one isomer of MIL-53­(In)-fum showing unexpected flexibility.^37^ It is notable that MIL-88A homologues have previously only been isolated with *d*-block metals, in contrast to MIL-53-fum which has been isolated only with *p*-block metals.^38^ The fact that the flexibility of MIL-53­(M)-fum seems to be both metal ion and conformational isomer dependent underpins the need for the synthesis of additional metal homologues in an isomerically selective manner.

We have previously reported the modulated self-assembly of MIL-88A­(Sc) and a related formate-linked analogue, and investigated their structural flexibilities in response to solvent exchange.^39^ Herein we describe the serendipitous observation that immersion of MIL-88A­(Sc) in aqueous media results in the spontaneous recrystallization of different conformational isomers of MIL-53­(Sc)-fum, a MOF that we have not been able to synthesize directly. The flexibility and postsynthetic modification of the different polymorphs are investigated, and we show that cluster anion substitution of the bridging μ_2_–OH units in the SBUs for μ_2_-OCH_3_ groups is facile and endows these flexible MOFs with permanent porosity.

## Results and Discussion

Having prepared MIL-88A­(Sc) by solvothermal synthesis in *N*,*N*-dimethylformamide (DMF), modulated with ten equivalents of HCl per metal ion, we sought to examine its solvent induced flexibility. As previously reported, soaking a sample of as-synthesized MIL-88A­(Sc) (Figure 2a) in water for 1 h results in significant swelling, with an increase in unit cell volume (*V*) from 2073.8(11) Å^3^ (single crystal X-ray diffraction) to 2410(16) Å^3^ (Pawley fit of powder X-ray diffraction data).^39^ After the sample had been immersed in water for 10 days, colorless block-shaped crystals were evident which, on analysis by single crystal X-ray diffraction, were found to comprise MIL-53­(Sc)-fum (see SI, Section S2). For this study we term MIL-53­(Sc)-fum as **1**, and use a specific naming scheme (SI, Section S2.1) to describe samples synthesized under different conditions and at different stages of activation.

We have previously identified three possible conformational isomers of MIL-53­(M)-fum based on the relative orientations of the fumarate linkers with respect to one another.^37^ The crystal structure of **1**-100, a sample of **1** synthesized from MIL-88A­(Sc) in 100% H_2_O, was solved from data collected by the EPSRC National Crystallography Service^40^ and, despite some minor disorder which could not be fully modeled (SI, Section S2.2), shows the MOF adopts the type (*a*) conformational isomer. The structure is closely related to those reported for the hydrated Al^35^ and Ga^34^ homologues derived from powder X-ray diffraction data. The diamondoid pores are filled with heavily disordered, partially occupied water molecules (Figure 2b), which form hydrogen bonded networks with themselves and the μ_2_–OH groups of the inorganic SBU (Figure 2c). The lack of order and apparent mobility of the water molecules within the pores suggest noncovalent interactions with the host are not significantly structure directing, and may account for the centrosymmetric conformation of the **1**-100 crystal structure.

**Figure 2:**
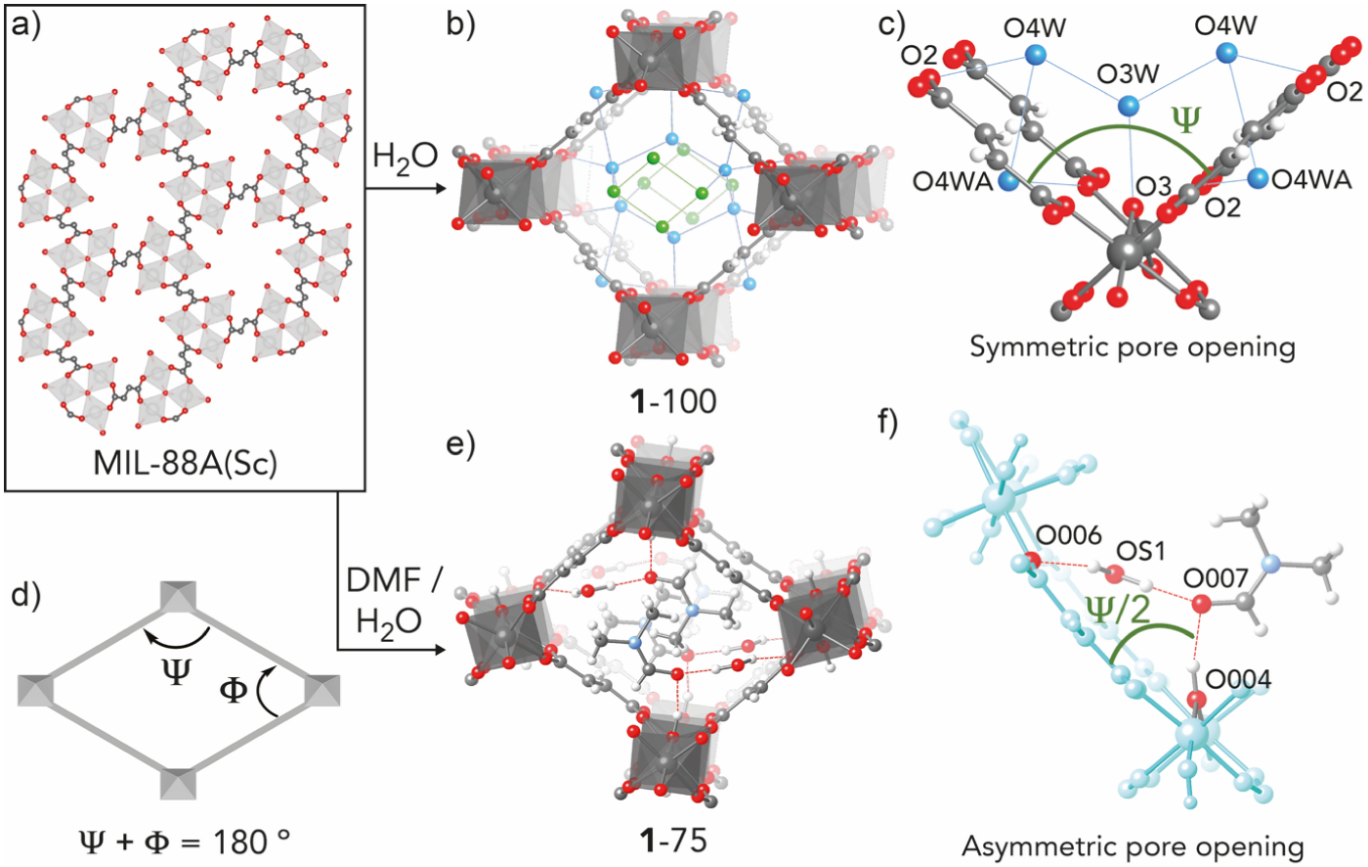
Conversion of a) MIL-88A­(Sc) to different isomers of MIL-53­(Sc)-fum. Portions of the crystal structure of **1**-100 depicting b) different hydrogen bonded networks of H_2_O within the internal pore and c) a section of the diamondoid pore showing the effect of hydrogen bonded H_2_O molecules on the symmetry of the pore opening, angles of which are summarized in part d). Selected H-bonds: O3···O3W = 2.75(2) Å, μ_2_–OH to H_2_O; O2···O4WA = 2.95(5) Å, COO to H_2_O; O3W···O4WA = 2.91(5) Å, H_2_O to H_2_O; Ψ = 95.14(1)°; Φ = 84.86(1)°. Sc: light gray polyhedra; C: dark gray; O: red; H: white. Oxygen atoms of pore-located H_2_O colored blue and green. Disorder removed for clarity. Portions of the crystal structure of **1**-75 (main component) depicting e) hydrogen bonded H_2_O and DMF guests within the internal pore and f) a section of the pore showing the asymmetric nature of the pore opening and lowered overall symmetry due to the differing solvent molecules in the pores. O007···O004 = 2.754(7) Å, μ_2_–OH to DMF; O007···OS1 = 2.844(7) Å, DMF to H_2_O; O006···OS1 = 3.141(7) Å, COO to H_2_O; Ψ = 99.89(1)°; Φ = 78.37(2)°. C: dark gray; N: light blue; O: red; H: white. Atoms of the framework depicted in pale turquoise. Disorder removed for clarity.

To assess the level of pore “openness”, the angles Φ and Ψ, corresponding to the smaller and larger of the two internal vertices of the rhombic pore, respectively, are measured (Figure 2d). We have successfully used this parameter to assess flexibility across related MIL-53 topology materials as it does not have the temperature sensitivity of unit cell volumes;^41,42^ the closer to 90° that Φ and Ψ approach, the more open the pore. In **1**-100, the value of Φ = 84.86(1)° indicates that the pore bound solvent holds the MOF in an open pore form.

Bulk samples of **1**-100 were isolated in the as-synthesized (*as*) form by drying in a vacuum desiccator. Compositional analysis (SI, Section S2.2) of the bulk material, **1**-100*as*, by solution ^1^H NMR spectroscopy after acid digestion showed the presence of DMF, presumably retained from the MIL-88A­(Sc) starting material (a partial DMF solvate). The molar ratio with the fumarate linker yielded a predicted formula of [Sc­(OH)­(fum)]·DMF_0.5_, which correlated closely with elemental analysis (experimental: N, 3.08%; calculated: N, 3.30%) and TGA data, that show a mass loss of 14% between 120 and 180 °C ascribed to DMF removal (calculated: 15.5%) and a Sc_2_O_3_ residue of 32.0 wt % (calculated: 32.4 wt %). These data suggest any pore-bound water was removed during the drying process. While it was possible to model water in the pores of the crystal structure, DMF could not be visualized in this particular crystal.

The presence of DMF in **1**-100*as* led us to study the role of solvent in the MOF-to-MOF synthesis of **1**, by immersing MIL-88A­(Sc) in DMF/H_2_O mixtures and assessing the structural transformation (if any) at different time points by powder X-ray diffraction. **1**-100*as* formed cleanly in approximately 5 h in pure water (Figure 3a). Full conversions were also observed when MIL-88A­(Sc) was immersed in 75% (*v*/*v*) and 50% (*v*/*v*) water in DMF, to give **1**-75 (Figure 3b) and **1**-50 (SI, Figure S13), respectively, taking approximately 48 h, but only partial conversion was observed in 25% (*v*/*v*) water in DMF even after 10 days. As such, **1**-25 was not further pursued (see SI, Section S2.5).

**Figure 3:**
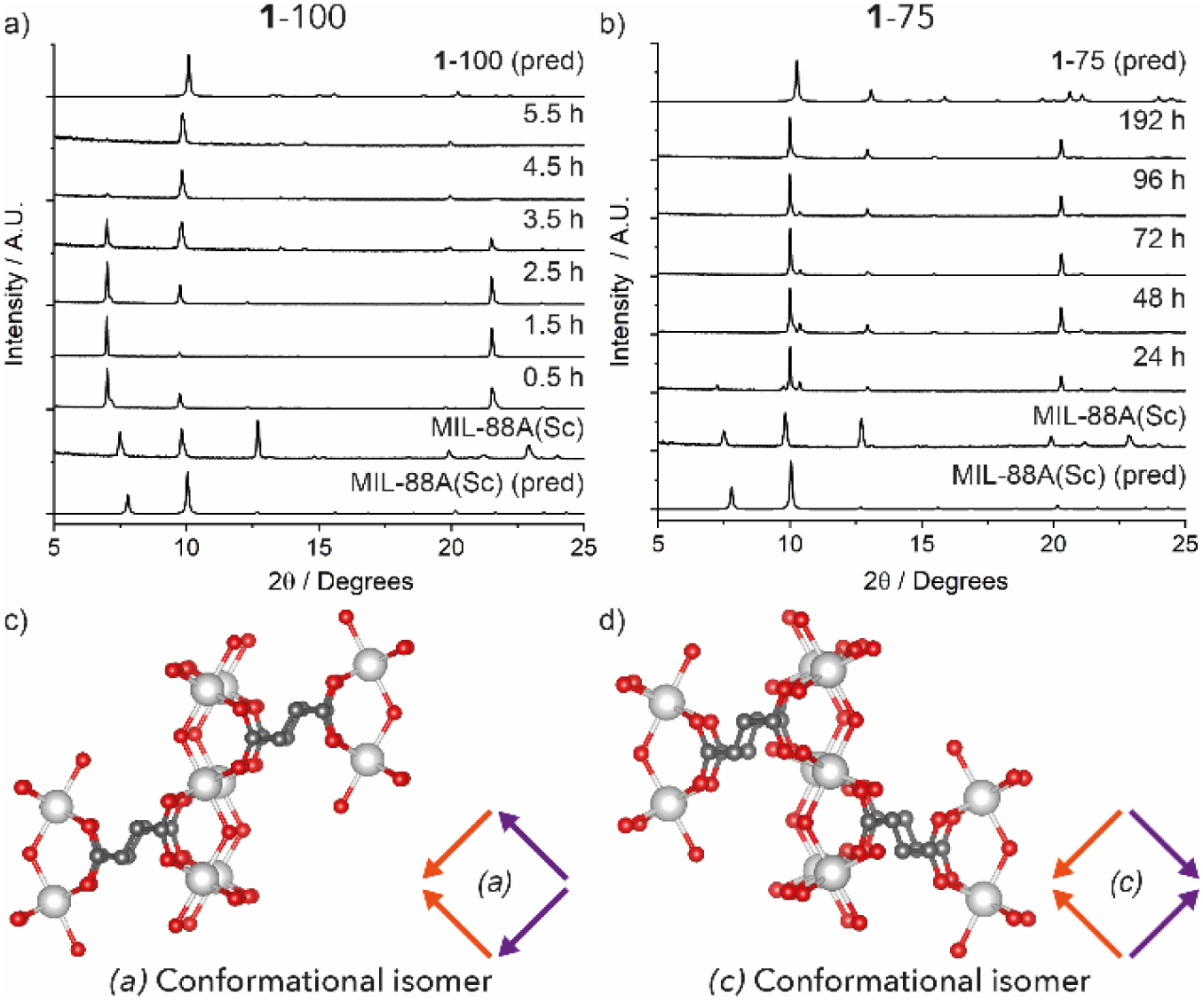
Stacked partial powder X-ray diffractograms showing the conversion of MIL-88A­(Sc) to MIL-53­(Sc)-fum over time in a) 100% H_2_O to form **1**-100, and b) 75% (*v*/*v*) H_2_O in DMF to form **1**-75. Sections of the crystal structures of c) **1**-100, and d) the major component of **1**-75, to show their formation of the *(a)* and *(c)* conformational isomers of MIL-53­(Sc)-fum, respectively. Sc: light gray; C: dark gray; O: red. H atoms and disorder removed for clarity.

The single crystal structure of **1**-75 was obtained from a sample that had been immersed in 75% (*v*/*v*) water in DMF for 10 days. The crystal structure revealed that **1**-75 crystallizes in the chiral *P*2_1_2_1_2_1_ orthorhombic space group with ordered DMF and water molecules bound within the pores, to give an overall crystallographic formula of [Sc­(OH)­(fum)]·DMF·H_2_O. Despite disorder, with three orientations of the fumarate linkers and two orientations of the Sc SBUs observable, the structure refines and converges to *R*_1_ = 4.7% (SI, Section S2.3). The occupancies of the three linker orientations were competitively refined to give one full fumarate, leading to values of 79.3%, 11.3% and 9.4%; the two orientations of the SBUs were also refined to give a main component of 89% and a minor component of 11%. These occupancies make sense as one of the disordered components of fumarate (refined as 11.3%) bonds exclusively to the minor component of the SBU (refined to 11%), whereas the two other fumarate orientations (refined to a total occupancy of 88.7%) bond through the major SBU component (refined to 89% occupancy). Each of these individual components of the crystal structure of **1**-75 comprise a conformational isomer of **1**-100, adopting the type (*c*) structure (Figure 3c and 3d) that we have previously observed in the single crystal structure of the indium homologue synthesized in DMF.^37^ While it is not possible to describe the average structure as a pure isomer, the major component (80%) is unequivocally isomer *(c)*, and the orientations of the minor components would suggest that isomer *(c)* is strongly favored under these solvent conditions.

This type of conformational isomerism has been observed in related MOFs containing similar nonlinear ligands,^43,44^ but these two structures unequivocally demonstrate by single crystal diffraction, for the first time, that MIL-53­(Sc)-fum (and by extension, the other metal homologues in the series) can adopt different conformational isomers when linked by the same metal. To exclude the possibility of correlated disorder seen in related MOFs such as DUT-8,^44^ precession images were synthesized. **1**-75 shows no diffuse scattering, while **1**-100 exhibits some within the *0kl* plane (SI, Figure S2), which we have attributed to the severely disordered and mobile water molecules found within the diamondoid pore that runs down the crystallographic *a* axis.

An array of DMF molecules is found along the one-dimensional channel of **1**-75, forming hydrogen bonds with both the μ_2_–OH units and pore-bound water molecules. The water molecules then form hydrogen bonds with oxygen atoms from the fumarate linkers (Figure 2e). As a result, hydrogen bonds between the above four components lead to the asymmetrical opening angle between two adjacent fumarate linkers, resulting in a change in the pore shape and a lowering of the total symmetry of **1**-75 compared to that of **1**-100 (Figure 2f). Despite the differing symmetries, both structures have unit cells that contain four fumarate linkers, four Sc^3+^ cations and four OH^–^ anions. **1**-100 (*I*2/*m*, *V* = 1123.7(3) Å^3^) has a slightly larger unit cell volume than **1**-75 (*P*2_1_2_1_2_1_, *V* = 1100.94(5) Å^3^), indicating it is slightly more open. The internal pore angle of **1**-100 (Φ = 84.86(1)°) is slightly larger than that for the major component of the structure of **1**-75 (Φ = 78.37(2)°), confirming this observation. The crystal structure of **1**-75 is reminiscent of our previously reported^37^ DMF solvate of MIL-53­(In)-fum prepared by conventional solvothermal synthesis; both are orthorhombic, contain pore-bound DMF, and have the same relative fumarate orientations. Together, these structures suggest that DMF may direct the formation of one conformational isomer of MIL-53­(M)-fum, while water induces the others.

As-synthesized materials were isolated after drying in a vacuum desiccator (SI, Section S2.3). Bulk samples of **1**-75*as* were confirmed to retain 1 mol of DMF per fumarate linker after drying by ^1^H NMR spectroscopy of acid digests. It is likely that water is again lost during the drying process: data from elemental analysis (experimental: N, 5.49%; calculated: N, 5.62%) and TGA (experimental Sc_2_O_3_ residue: 27.8 wt %; calculated Sc_2_O_3_ residue: 27.7 wt %) correlate closely with a bulk formula of [Sc­(OH)­(fum)]·DMF. With no single crystals of **1**-50 available, powder X-ray diffraction analysis of **1**-50*as* resulted in a very similar diffractogram to that of **1**-75*as* (Figure 4a), while similar bulk compositional analysis confirmed the same formula of [Sc­(OH)­(fum)]·DMF (SI, Section S2.4). This would suggest the as-synthesized materials have the same structure and relative fumarate orientations, i.e., both form type (*c*) conformational isomers, and chemical composition.

**Figure 4:**
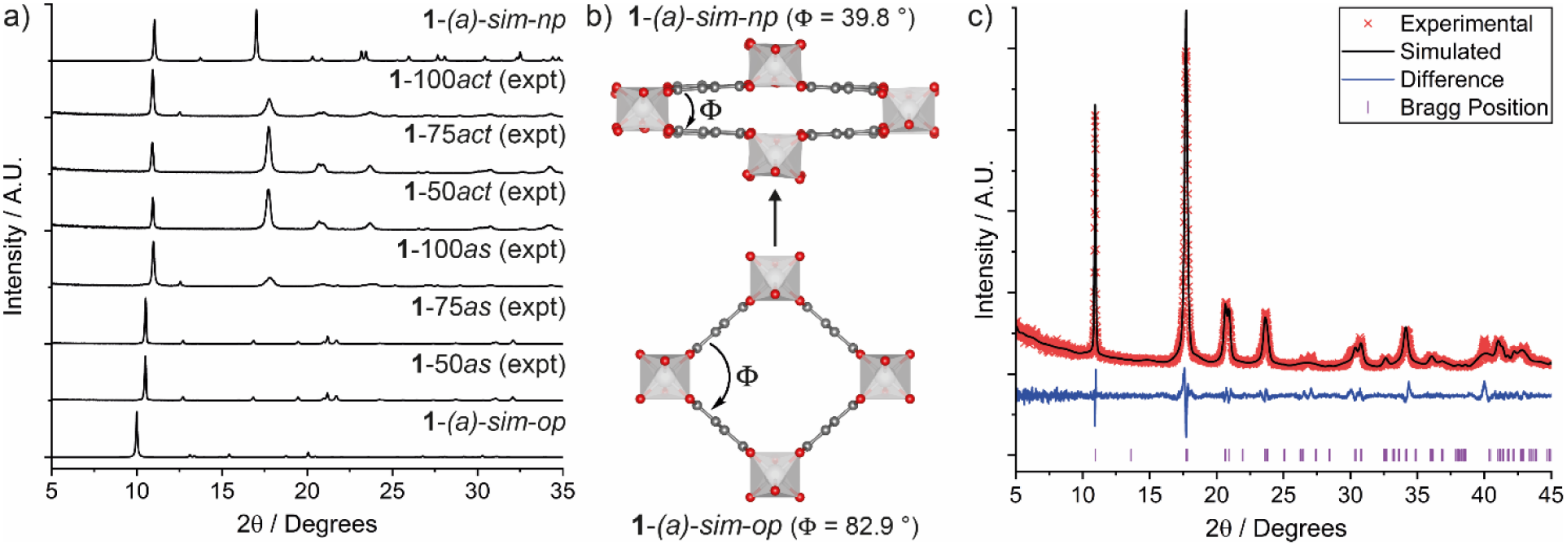
a) Stacked partial powder X-ray diffractograms (room temperature) for as-synthesized (as) and activated (act) samples of **1**-100, **1**-75, and **1**-50 compared to diffractograms derived from DFT optimized open pore (**1**-*(a)-sim-op*) and narrow pore (**1**-*(a)-sim-np*) structures. b) Portions of the DFT optimized structures of the *(a)* conformational isomer in the open and narrow pore forms. The pore opening angle, Φ, is indicated on both structures. Sc: light gray polyhedra; C: dark gray; O: red. H atoms removed for clarity. c) Pawley fitting of the diffractogram of **1**-50*act*. The fit yielded a proposed structure (*a* = 7.569 Å, *b* = 16.495 Å, *c* = 5.603 Å, β = 106.99°, *V* = 669 Å^3^, *R*_p_ = 8.00%, *R*_wp_ = 10.32%) with Φ = 37.5°.

Water has previously been shown to induce transformation of related Fe MOFs into denser, more thermodynamically favorable phases, including the formation of MIL-53­(Fe)_2CH3 from MIL-101­(Fe)_2CH3, where the ligand is 2,5-dimethylbenzene-1,4-dicarboxylate.^45^ This effect was attributed to the oxophilicity of Fe^3+^; Sc^3+^ is a stronger Lewis acid and is therefore even more oxophilic, which may be reflected in the fact that MIL-88A­(Fe) has recently been studied for water adsorption with no indication of any water-induced transformation as observed here for the Sc homologue.^46^ Additionally, the transformation of MIL-88A­(Sc) into MIL-53­(Sc)-fum can even be induced by placing MIL-88A­(Sc) into a humid chamber (SI, Section S2.8); full immersion in water is not required. Analysis by SEM (SI, Section S2.9) showed that the morphology of the particles changes completely on conversion, from the characteristic hexagonal prismatic needles of MIL-88A­(Sc) to smaller rectangular rods for each MIL-53­(Sc)-fum sample, which suggests a dissolution-recrystallization mechanism. It is noteworthy that the previous literature examples of MOFs generated by solvent-induced phase transitions cover examples that had previously been synthesized directly. In our hands, all attempts to prepare MIL-53­(Sc)-fum by direct self-assembly from metal salts and fumaric acid were unsuccessful, including using conditions resembling those which induce conversion from MIL-88A­(Sc).

Comparison of the powder X-ray diffractograms of freshly crystallized samples of **1**-100, **1**-75, and **1**-50 with those after drying (**1**-100*as*, **1**-75*as*, and **1**-50*as*) indicated some structural changes ascribed to flexibility, with differences in the diffractograms observed for **1**-100*as* (synthesized in pure water) compared to **1**-75*as* and **1**-50*as* (synthesized in water/DMF mixtures). **1**-100*as* adopts a more closed pore form than **1**-75*as* and **1**-50*as*, which is evident on comparing their diffractograms (Figure 4a) and likely originates in the lower DMF content of **1**-100*as* compared to the other samples. Variable temperature powder X-ray diffraction (SI, Section S3.2) showed no structural change on heating for **1**-100*as*, while both **1**-75*as* and **1**-50*as* exhibited a temperature-induced phase change to a more closed pore form, presumably on removal of solvent, which was confirmed by ^1^H NMR spectroscopy of acid digests. Activating the samples by heating to 423 K under 10^–4^ Torr vacuum for 20 h led to the activated (*act*) materials, **1**-100*act*, **1**-75*act*, and **1**-50*act*, showing broadly the same powder X-ray diffractogram, but with differences in peak intensities and broadness when comparing **1**-100*act* to **1**-75*act* and **1**-50*act* (Figure 4a). This may result from the closed pore forms of the different conformational isomers being crystallographically very similar, or interconversion between these forms, by concerted linker rotations, under the activation condition.

To interpret the extent of flexibility, density functional theory (DFT) calculations were performed (SI, Section S3.1). Starting from the crystal structures of **1**-100 (conformational isomer (*a*)) and **1**-75 (conformational isomer (*c*)), simulated (*sim*) models were generated in the open pore (*op*) form, which were named **1**-*(a)*-*sim*-*op* and **1**-*(c)*-*sim*-*op*, respectively, for the two conformational isomers. To generate narrow pore (*np*) structures, the DFT optimized open pore structures were used as the starting points, and further geometry optimizations of the structures using DFT under a hydrostatic pressure of 1 GPa were run, which promoted open-to-narrow pore phase transitions. Subsequently, the hydrostatic pressure was removed and the narrow pore structures reoptimized under ambient pressure, where the narrow pore structures remained energetically stable. The DFT optimized narrow pore structures for the two conformational isomers were named **1**-*(a)*-*sim*-*np* and **1**-*(c)*-*sim*-*np*, respectively. Portions of the structures of **1**-*(a)*-*sim*-*op* and **1**-*(a)*-*sim*-*np* are shown in Figure 4b. Predicted diffractograms derived from the simulated structures **1**-*(a)*-*sim*-*np* and **1**-*(c)*-*sim*-*np* show subtle differences (SI, Figure S17), but in general correlate well to the experimental diffractograms, confirming significant closing in all three activated samples (Figure 4a). The broadness of some of the Bragg peaks in the experimental diffractograms, however, precludes any conclusions being made as to whether the conformational isomers can interconvert under the activation conditions. The pore angle Φ was used to assess the level of pore “openness” across the different conformational isomers in both the experimental and simulated structures (Figure 4b). The crystal structures of **1**-100 (*a* isomer) and **1**-75 (*c* isomer) gave Φ = 84.86(1)° and Φ = 78.37(2)°, respectively (Figure 2), while the DFT optimized open pore models **1**-*(a)*-*sim*-*op* and **1**-*(c)*-*sim*-*op* give Φ = 82.9° and Φ = 77.9°, and all are in agreement with an open pore form. In contrast, **1**-*(a)*-*sim*-*np* and **1**-*(c)*-*sim*-*np* predict significant pore contraction, giving values of Φ = 39.8° and Φ = 38.0°, respectively.

Pawley fitting of the diffractogram of **1**-50*act* (as representative of the highly similar diffractograms for all activated samples, Figure 4c) in the *I*2/*m* space group gave a unit cell volume (*V*) of 669 Å^3^ (*R*_wp_ = 10.32%), which represents a contraction in volume of 40.5% compared to the single crystal structure of **1**-100 (*I*2/*m*, *V* = 1123.7(3) Å^3^) and of 39.2% compared to the single crystal structure of **1**-75 (*P*2_1_2_1_2_1_, *V* = 1100.94(5) Å^3^). The formation of a closed pore material on activation, indicated by the fit yielding a much smaller pore angle (Φ = 37.5°, correlating closely with the DFT models), was confirmed by the lack of N_2_ adsorption at 77 K by **1**-100*act*, **1**-75*act* and **1**-50*act* (SI, Figure S24). All the MIL-53­(Sc)-fum samples are essentially nonporous to N_2_, in contrast to their rigid Al, Ga, and In homologues.^30,34,36^

To fully assess the flexibility of the samples, we turned to solvent exchange to optimize activation. Samples of **1**-100*as*, **1**-75*as*, and **1**-50*as* were refluxed in either acetone (*ace*) or methanol (*MeOH*) for 16 h, followed by isolation by centrifugation, drying in a vacuum desiccator, then activation by heating to 423 K under 10^–4^ Torr vacuum for 20 h. Recently, we have shown that postsynthetic modification can occur at the bridging hydroxyl units of the MOF GUF-1­(Sc),^41^ which has the same infinite 1D chain [Sc­(μ_2_–OH)­(RCO_2_)_2_]_*n*_ inorganic SBU as **1**, through substitution with methanol to form bridging μ_2_-OCH_3_ units under either high pressure or high temperature conditions.^6^ This cluster anion substitution process is a novel alternative mode of postsynthetic modification that takes advantage of the labile nature of the small anionic ligands often required to form specific SBUs to further diversify MOF structures. In addition, it has been reported that synthesis of MIL-53­(In)-fum in MeOH results directly in a methoxylated MOF with a hydrophobic pore interior,^34^ complementing reports of the direct synthesis in MeOH of other MOFs with the MIL-53 topology containing methoxylated SBUs.^47,48^ MIL-53­(Mn)-OCH3 was shown to be unexpectedly structurally rigid, suggesting methoxylated SBUs can influence framework flexibility;^47^ activation of the varying samples of **1** in MeOH was therefore attempted to determine the extent of any methoxylation of the SBUs and any resulting effect on flexibility.

Acetone exchange under reflux conditions did not remove the entrapped DMF from as-synthesized samples of **1** (SI, Section S3.3). Samples of **1**-100*ace*, **1**-75*ace*, and **1**-50*ace* all retained significant amounts of DMF after drying in a vacuum desiccator, as indicated by TGA and NMR spectroscopic analyses, while powder X-ray diffractograms suggested the MOFs were in an open pore form (SI, Figure S31). After activation and the collection of N_2_ adsorption/desorption isotherms (77 K), **1**-100*ace-act*, **1**-75*ace-act*, and **1**-50*ace-act* adopted a closed pore form, and contained only trace solvent according to NMR spectroscopy (which could conceivably be through contamination) and TGA (SI, Table S5). The powder X-ray diffractograms (SI, Figure S34) closely resembled those the analogous samples which were directly activated (**1**-100*act*, **1**-75*act*, and **1**-50*act*) as well as the DFT models for the closed pore materials, clearly indicating the application of heat and vacuum was required to remove the pore-bound DMF. Pawley fitting of the diffractogram of **1**-50*ace*-*act* (SI, Figure S35) as representative of the acetone-refluxed and activated samples gave very similar unit cell data (*I*2/*m*, *V* = 665 Å^3^, w*R*_p_ = 11.04%) to the directly activated structures, with a similarly contracted pore angle Φ = 37.4°. The formation of a closed pore structure was again confirmed for **1**-100*ace-act*, **1**-75*ace-act*, and **1**-50*ace-act*, with the MOFs exhibiting negligible N_2_ uptake at 77 K (SI, Figure S38).

In contrast, refluxing **1**-100*as*, **1**-75*as*, and **1**-50*as* in MeOH (SI, Section S3.4) resulted in near complete removal of DMF. Samples that had been dried in a vacuum desiccator, **1**-100*MeOH*, **1**-75 *MeOH*, and **1**-50*MeOH*, showed only trace amounts of DMF but displayed a resonance assigned to MeOH in ^1^H NMR spectra of acid digested samples, suggestive of significant cluster anion substitution of μ_2_–OH to μ_2_-OCH_3_. The resonances were retained in ^1^H NMR spectra of digested samples even after activation by heating to 423 K under 10^–4^ Torr vacuum for 20 h, strong indication that they correspond to μ_2_-OCH_3_ groups directly bonded to the framework backbones (subsequently released as MeOH by acid digestion) rather than physisorbed methanol solvent molecules.^6^ Integration of the resonances assigned to the proposed μ_2_-OCH_3_ and fumarate linker C–H protons allowed quantification of cluster anion substitution by comparing the fumarate to methanol stoichiometric ratio with a general formula [Sc_1_(fum)_1_(OH)_1–*x*_(OCH_3_)_*x*_]. **1**-100*MeOH-act* showed 37% substitution of μ_2_–OH to μ_2_-OCH_3_; **1**-75*MeOH-act* showed 36% substitution; and **1**-50*MeOH-act* showed 31% substitution. These values correlate well with compositional analysis by TGA and elemental analysis (full formulas are provided in the SI, Table S7). A control experiment was carried out, where **1**-100*as* was solvent exchanged with MeOH at room temperature, and only trace methanol was present in **1**-100*MeOH*-*RT*-*as*, even before activation (SI, Figure S47). While room temperature MeOH exchange successfully removed all DMF from the pores, the results indicate reflux conditions are required to induce SBU methoxylation, consistent with our previous work on GUF-1­(Sc).^6^

Solid-state NMR spectroscopy was used to gain a better understanding of the postsynthetic cluster anion exchange in **1**-75 on the bulk scale, through analysis of as-synthesized and methanol-activated samples; **1**-75*as* and **1**-75*MeOH-as*, respectively. Initially, ^13^C cross polarized (CP) MAS NMR spectra were acquired. The resonances at 174 and 139 ppm can be assigned to carbon atoms in the fumarate linker: the carbonyl carbon (C=O) and alkene carbons (C=C), respectively, based on their chemical shifts. The presence of DMF was confirmed in **1**-75*as*, as indicated by the three resonances at 164, 37, and 34 ppm (Figure 5, bottom). When the sample is reacted to give **1**-75*MeOH-as*, the intensities of these three resonances decrease, indicating DMF is removed during the exchange process (Figure 5, middle). An additional resonance emerges at 54 ppm, which is assigned to μ_2_-OCH_3_. This is a similar chemical shift to that observed previously (56 ppm) for μ_2_-OCH_3_ exchanged GUF-1­(Sc).^6^ A quantitative, direct polarization ^13^C MAS NMR spectrum (SI, Figure S48) was acquired for **1**-75*MeOH-as* following measurement of the T_1_ relaxation time (see SI for details) to ensure the spectrum is quantitative. Fitting this spectrum and comparing the intensities of the resonances for the fumarate and OCH_3_ carbon atoms indicates a 1:1.37(3) stoichiometry of fum:OCH_3_. Given the general formula [Sc_1_(fum)_1_(OH)_1–*x*_(OCH_3_)_*x*_] shows that a fully methoxylated material would result in a 1:1 stoichiometry, this ratio indicates that the signal likely arises from overlapping contributions from coordinated μ_2_-OCH_3_ and pore-bound methanol. **1**-75*MeOH-as* was therefore calcined using conditions that mimic activation (423 K, 20 h, 10^–4^ Torr), to yield a sample similar to **1**-75*MeOH-act*; retention of the resonance assigned to μ_2_-OCH_3_ confirms it is bonded to the SBU, particularly as the resonances assigned to residual pore-bound DMF disappear (Figure 5, top). Fitting of the quantitative ^13^C MAS NMR spectrum of calcined **1**-75*MeOH-as* (SI, Figure S49) gives a fum:OCH_3_ stoichiometry of 1:0.56(3); in other words, 56(3)% cluster anion substitution of μ_2_–OH to μ_2_-OCH_3_ has occurred, significantly higher than the percentage conversion on bulk samples of GUF-1­(Sc), which showed a conversion of ∼20%.^6^

**Figure 5:**
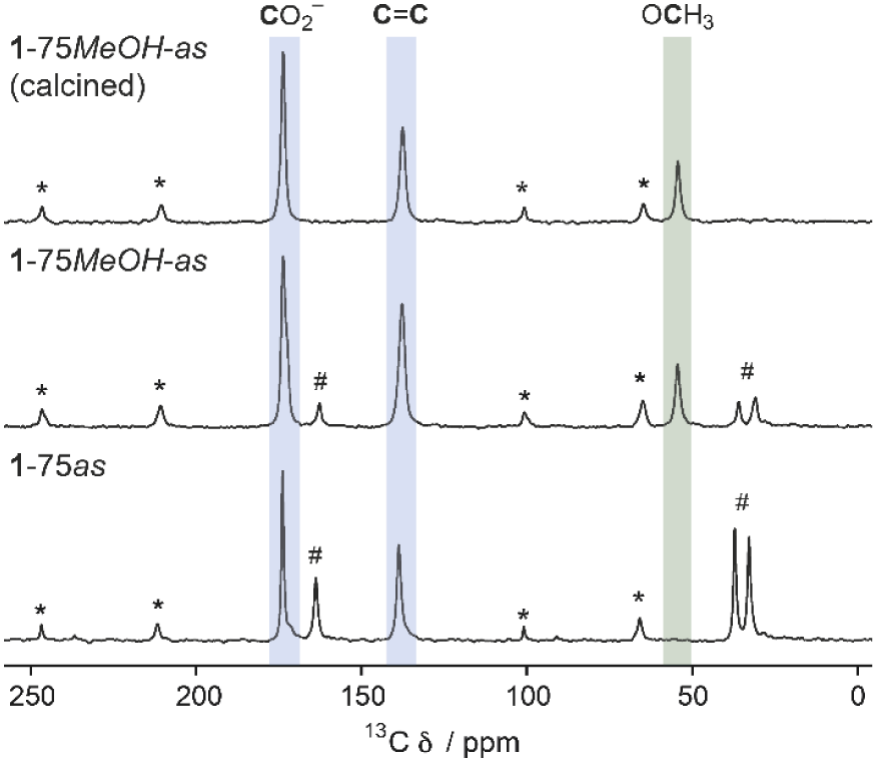
Stacked ^13^C (12.5 kHz MAS, 14.1 T) CP MAS NMR spectra of **1**-75*as*, **1**-75*MeOH-as* and **1**-75*MeOH-as* (calcined). Blue shading highlights resonances corresponding to the carbon atoms of the fumarate ligand and green shading highlights the resonance assigned to the μ_2_-OCH_3_ carbon. Hash symbols (#) denote resonances assigned to the carbon atoms of DMF, and asterisks (*) denote spinning sidebands.

The quantitative ^13^C MAS NMR spectra contain a broad background signal which has been taken into account when fitting the signals from the fumarate linker and μ_2_-OCH_3_ peaks, which may induce some uncertainty in the absolute intensity value. Nevertheless, the conclusions derived from the solid-state NMR spectra agree well with the extent of μ_2_-OCH_3_ exchange (36%) estimated from acid digestion liquid NMR spectroscopy of **1**-75*MeOH-act*, in combination with elemental analysis and TGA (SI, Table S7), and confirms that chemical exchange of the μ_2_–OH bridge is seen and not merely the presence of residual solvent in the pores of the framework.

We have previously postulated^6^ that the mechanism for this process involves methanol coordination to the SBU, followed by proton transfer from coordinated CH_3_OH to coordinated OH^–^ (generating coordinated OCH_3_^–^ and H_2_O), followed by loss of H_2_O. This process could conceivably involve 6-coordinate Sc^3+^ cations, if the cluster anions bind in a μ_1_ fashion, or 7-coordinate Sc^3+^ cations, if they bind with bridging μ_2_ motifs (see SI, Figure S50 for plausible mechanisms). Given the propensity for Sc^3+^ to adopt a 7-coordinate geometry,^49^ we expect that steric hindrance around the SBU will influence the actual mechanism of cluster anion substitution in this case; mechanistic investigations will form the basis of future study, particularly given the proposed importance of such cluster bound methoxides in catalytic processes.^50^

With methoxylation confirmed by complementary spectroscopic techniques, the effect on framework flexibility was examined. Comparison of the powder X-ray diffractograms of **1**-100*MeOH-act*, **1**-75*MeOH-act*, and **1**-50*MeOH-act* with the acetone-refluxed analogues showed notable differences. The diffractograms of the three methanol-refluxed, activated samples were very similar, but the most intense Bragg peak was found at lower values of 2θ than in the three acetone-refluxed analogues, which is indicative that the methoxylated materials display reduced flexibility and adopt a more open-pore structure on activation (Figure 6a). Pawley fitting of the diffractogram of **1**-50*MeOH*-*act* as representative of the samples (SI, Figure S55) gave a significantly larger unit cell volume (*I*2/*m*, *V* = 835 Å^3^, w*R*_p_ = 9.65%) compared to the acetone-refluxed and directly activated samples (V ∼ 660–670 Å^3^). This is consistent with the increased steric bulk of the μ_2_-OCH_3_ units coordinated to the SBUs, compared to μ_2_–OH, which effectively hold the structure in this more open form; the pore angle predicted from the fit is significantly increased (Φ = 52.7°) compared to the nonmethoxylated samples (Φ ∼ 37.5°). In contrast, the diffractograms for the room temperature, methanol-exchanged sample after activation, **1**-100*MeOH*-*RT*-*act* (Figure 6a), closely resembles those of the simulated and experimental closed pore structures, further suggesting that methoxylation does not take place at room temperature.

**Figure 6:**
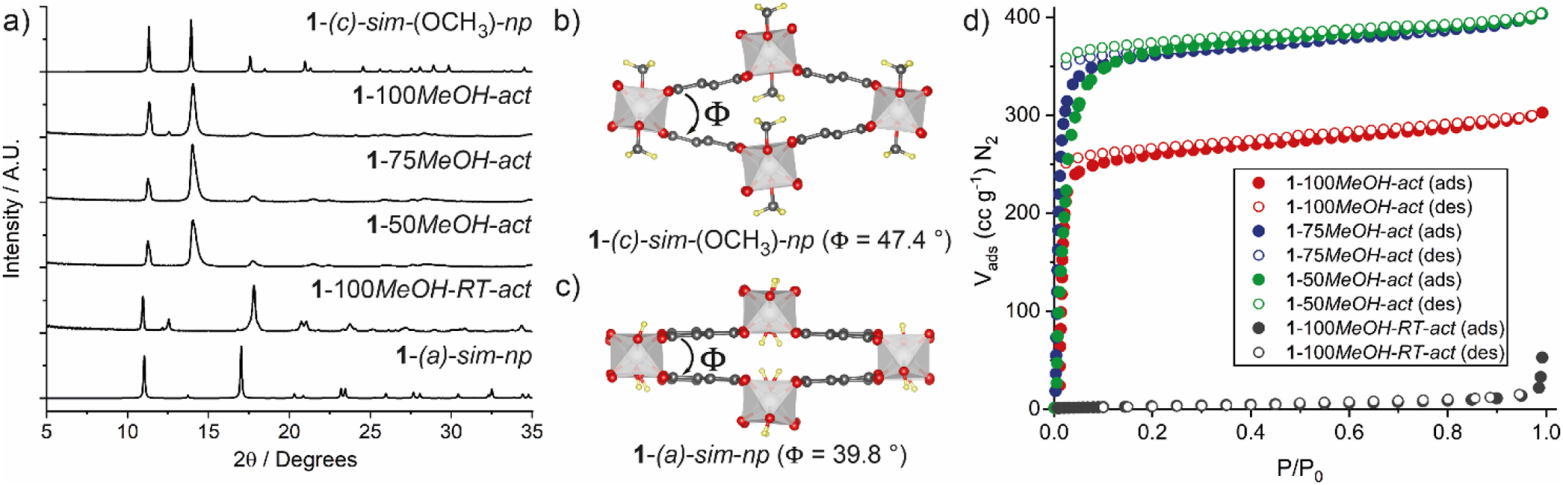
a) Stacked partial powder X-ray diffractograms of methanol refluxed, activated samples compared to that of a control soaked in methanol at room temperature then activated (**1**-100*MeOH-RT-act*), and those predicted from the DFT models of **1**-*(a)-sim-np* and fully methoxylated **1**-*(c)-sim*-(OCH_3_)-*np*. Representations of the DFT model structures of b) **1**-*(a)-sim-np* and c) **1**-*(c)-sim*-(OCH_3_)-*np*, showing the effect of methoxylation on pore opening angle, Φ. d) N_2_ adsorption/desorption isotherms (77 K) of the methanol refluxed, activated samples, which show porosity due to methoxylation, compared to the nonporous **1**-100*MeOH-RT-act*.

This difference in flexibility after methoxylation was assessed by additional DFT simulations on structures of **1** (in both the (*a*) and (*c*) conformational isomers) with varying degrees of methoxylation. In addition to the previously described **1**-*(a)*-*sim* and **1**-*(c)*-*sim* native structures, the additional simulations were named **1**-*(a)*-*sim*-(OCH_3_) and **1**-*(c)*-*sim*-(OCH_3_) for fully methoxylated materials, and **1**-*(a)*-*sim*-(OCH_3_)_0.5_(OH)_0.5_ and **1**-*(c)*-*sim*-(OCH_3_)_0.5_(OH)_0.5_ for intermediates where 50% of the OH groups had been exchanged for OCH_3_ (arranged so that μ_2_–OH and μ_2_-OCH_3_ units alternate along each SBU chain, see SI, Section S3.4.1). The suffixes -*op* and -*np* are again used to denote simulations in the open and narrow pore forms, respectively. The extent of methoxylation did not affect the configuration of the simulated open pore forms, but the effect on flexibility is clear; increasing methoxylation is predicted to yield less closed forms of **1** upon activation (Figures 6b and 6c). **1**-*(a)*-*sim*-(OCH_3_)_0.5_(OH)_0.5_ and **1**-*(c)*-*sim*-(OCH_3_)_0.5_(OH)_0.5_ predict expanded pore angles of Φ = 46.7° and Φ = 46.1°, respectively, which increase further on complete methoxylation to Φ = 47.1° and Φ = 47.4° for **1**-*(a)*-*sim*-(OCH_3_) and **1**-*(c)*-*sim*-(OCH_3_). Comparison of experimental diffractograms with those derived from the DFT models also results in excellent agreement (SI, Section 3.4.2). The methanol-exchanged samples, for example **1**-75*MeOH-act*, give diffractograms which are most closely reminiscent with the **1**-*sim*-(OCH_3_) models, in particular **1**-*(c)*-*sim*-(OCH_3_). This suggests that the partial methoxylation of the framework is randomly distributed, to induce pore opening across the material, rather than concentrated on only one side of the SBU as shown in **1**-*sim*-(OCH_3_)_0.5_(OH)_0.5_. Together, the combination of theory and experiment clearly show the effect of cluster anion substitution in tuning MOF flexibility.

It is noteworthy that the flexibility of all the MIL-53­(Sc)-fum samples reported herein contrasts with the reported rigidity of the Al, Ga and In homologues (other than the one conformational isomer of the In homologue, MIL-53­(In)-fum*(c)*, which is flexible),^34–36,51^ which could be a consequence of Sc^3+^ having a slightly larger ionic radius. This flexibility impacts their adsorption behavior. The acetone-refluxed and directly activated samples of **1** showed effectively no N_2_ uptake up to 1 bar at 77 K, which is to be expected given they adopt a closed pore form under these conditions; similar behavior is exhibited by the BDC-linked MIL-53­(Sc).^16^ In contrast, the methoxylated samples showed Type I isotherms typical of microporous materials for N_2_ adsorption, with uptakes in excess of those reported for the rigid *p*-block metal homologues (Figure 6d).^30,34,36^ BET areas were calculated using BETSI software (SI, Figures S57–S62);^52^ for **1**-75*MeOH-act* and **1**-50*MeOH-act* the values were 1483 m^2^ g^–1^ and 1491 m^2^ g^–1^, higher than the value of 1053 m^2^ g^–1^ found for **1**-100*MeOH-act*. The sample exchanged with methanol at room temperature **1**-100*MeOH*-*RT*-*act*, which did not undergo cluster anion substitution and formed a closed pore material, showed no appreciable N_2_ adsorption, in agreement with the other closed pore forms derived from direct and acetone activation.

While the two samples originally prepared in DMF/water mixtures had higher porosities than the sample originally synthesized in pure water, we are hesitant to ascribe this porosity difference to the difference in conformational isomerism in the as-synthesized materials, given that methanol-exchange (with associated cluster methoxylation) and activation yielded materials with closely related powder X-ray diffractograms which cannot unequivocally be assigned to a specific conformational isomer. We expect that this significant N_2_ uptake is a consequence of the steric bulk of the cluster-bound μ_2_-OCH_3_ groups preventing the frameworks from closing completely, allowing uptake of N_2_ at low partial pressures like a classic microporous material, with seamless opening of the structures as the partial pressure increases. Structural flexibility is also hinted by the long equilibration times needed to obtain the isotherms, and tentative hysteresis on the desorption profiles.

## Conclusions

This study has shown that MIL-53­(Sc)-fum can be accessed directly by immersion of the related scandium fumarate MOF, MIL-88A­(Sc), in water or aqueous DMF mixtures. Single crystal structures unequivocally show that MIL-53­(M)-fum MOFs can display conformational isomerism when linked by the same metal, in line with our previous predictions, and tentatively suggest solvent templation of specific conformational isomers during crystallization. The MIL-53­(Sc)-fum materials are the first definitively characterized examples of this MOF connected by a *d*-block metal, and show significant structural flexibility, which has been characterized experimentally and computationally, in contrast to their rigid homologues linked with group 13, *p*-block metals. This flexibility can be modulated by substitution of bridging μ_2_–OH moieties at the 1D chain SBUs with μ_2_-OCH_3_ units, achieved through refluxing the MOFs in methanol. With 40–50% cluster anion exchange, the extent of flexibility is greatly reduced due to the additional steric bulk located within the porosity, allowing the methoxylated MOFs to adsorb significant quantities of N_2_ at 77 K, in contrast to the effectively nonporous native structures.

These findings complement the small but growing number of studies that show conversion of a kinetic MOF phase into a more thermodynamically stable one under relatively mild recrystallization conditions can be a powerful technique to access specific materials; however, in our case this is made more significant by the fact that we have not yet uncovered conditions to directly synthesize MIL-53­(Sc)-fum from metal salts and fumaric acid. It also shows the potential in tuning the behavior of adsorbents through controlled substitution of cluster bound anions such as the OH units that are ubiquitous in MOFs; flexibility of related MIL-53 materials has previously been tuned through deliberate synthesis of analogues with μ_2_-F units at the SBU instead of μ_2_–OH,^53^ rather than postsynthetically. Indeed, this study complements a small but growing collection of examples wherein flexibility can be influenced by postsynthetic modification,^39,54^ highlighting its great potential for fine-tuning of MOF properties. Taken together, both methodologies serve to diversify the range of MOF adsorbents available and allow precise control over flexibility and adsorption.

## Supplementary Material



## Data Availability

The data that support the findings of this study can be obtained free of charge at https://doi.org/10.5525/gla.researchdata.2331.

## Notes

Experimental section; synthetic and postsynthetic modification protocols; materials characterization (X-ray diffraction, thermogravimetric analysis, solution and solid-state NMR spectroscopy, FTIR spectroscopy, elemental analysis, porosimetry); DFT simulations (PDF)
