# Supplementary material for: Cluster Anion Substitution Tunes Flexibility and Porosity of MIL-53(Sc)-fum Conformational Isomers Isolated by Solvent-Induced MOF-to-MOF Self-Assembly
Source: J Am Chem Soc. 2026 Jul 28;148(31):33206–16. doi: 10.1021/jacs.6c06041 (PMC13474951; doi:10.1021/jacs.6c06041)
Supplement: Supplementary file 1 [file ja6c06041_si_001.pdf]

Cluster Anion Substitution Tunes Flexibility and Porosity of MIL-53(Sc)-fum Conformational Isomers Isolated by Solvent-Induced MOF-to-MOF Self-Assembly

Catherine A. Walshe,[1] Zachary H. Davis,[2] Chi C. Hong,[3] Thach N. Tu,[1] Sanliang Ling,[4] Claire Wilson,[1] Joshua J. B. Levinsky,[3] Claire L. Hobday,[3] Sharon E. Ashbrook[2] and Ross S. Forgan[1*]

1. School of Chemistry, University of Glasgow, Joseph Black Building, University Avenue, Glasgow, G12 8QQ, UK. E-mail: Ross.Forgan@glasgow.ac.uk
2. EaStCHEM School of Chemistry and Centre of Magnetic Resonance, University of St Andrews, St Andrews, KY16 9ST, UK.
3. EaStCHEM School of Chemistry and Centre for Science at Extreme Conditions, University of Edinburgh, King's Buildings, David Brewster Road, Edinburgh, EH9 3FJ, UK.
4. Advanced Materials Research Group, Faculty of Engineering, University of Nottingham, University Park, Nottingham, NG7 2RD, UK

SUPPORTING INFORMATION

Table of Contents:

S1. Materials and Methods S2.
S2. Synthesis S5.
S2.1 Sample Naming Scheme S5.
S2.2. Crystallisation of **1**-100 and Characterisation of **1**-100*as* S6.
S2.3. Crystallisation of **1**-75 and Characterisation of **1**-75*as* S13.
S2.4. Crystallisation of **1**-50 and Characterisation of **1**-50*as* S19.
S2.5. Crystallisation of **1**-25 S23.
S2.6. Comparison of As-Synthesised Samples S24.
S2.7. Comparison of Crystallographic Data S28.
S2.8. Transformation by Humidity S30.
S2.9. Morphological Comparison S31.
S3. Activation and Flexibility S33.
S3.1. DFT Modelling S33.
S3.2. Activation by Temperature and Vacuum S34.
S3.3. Acetone Solvent Exchange and Activation S42.
S3.4. Methanol Solvent Exchange and Activation S54.
S4. References S77.

S1. Materials and Methods

Unless otherwise stated, all reagents were purchased from Alfa Aesar, Acros Organics, Tokyo Chemical Industry, Sigma Aldrich, Fluorochem or Strem and used without any further purification.

Thermogravimetric Analysis: TGA measurements of the materials was carried out in air using a Discovery Series TGA 5500, manufactured by TA Instruments, a Division of Waters. The gas flows are 10 mL min^{-1} balance and 25 mL min^{-1} over sample and the analysis was completed with a heating rate of 10 K min^{-1} and a heating range of 298 K to 1073 K. The data was analysed using the software TRIOS.

Powder X-Ray Diffraction: Room temperature PXRD patterns were collected using a benchtop Rigaku MiniFlex diffractometer at 298 K. Radiation was CuKα with a characteristic wavelength of 1.5418 Å. Scans ranged from 3-45 ° and were collected on spinning zero-background holders. All indexing and Pawley fitting was completed using Materials Studio software.

Variable Temperature X-Ray Diffraction: Variable-temperature PXRD patterns were collected on an Anton Paar HTK 1200N stage using a PANalytical X'Pert PRO diffractometer (monochromated λ ($CuK\alpha_1$ = 1.54056 Å) ranging from 298 K to 523 K in 25 K increments, then the sample was cooled in 50 K steps until room temperature.

Liquid State Nuclear Magnetic Resonance Spectroscopy: ^1H and ^{13}C NMR spectroscopic data were recorded on a 400 MHz Bruker AVIII spectrometer. MOFs were digested in DMSO-d_6/D_2SO_4 and all spectra were referenced to residual solvent resonances.

Single-Crystal X-Ray Diffraction: Data for **1**-100 and **1**-75 were collected at the National Crystallography Service at the University of Southampton[S1] using Cu Kα rotating anode radiation source (λ = 1.54178 Å). Single crystals were mounted on cryoloops and

diffraction data collected at 100 K using a Rigaku 007HF diffractometer equipped with a UG2 goniometer and HyPix 6000HE detector. The unit cell and data reduction were determined using CrysAlisPro software and a multi-scan absorption correction applied.

The crystal structures were solved *ab initio* with SHELXT[S2] and refined using SHELXL within OLEX2. Non-H atoms were refined with anisotropic atomic displacement parameters (ADPs) and H-atoms were placed in geometrically calculated positions and included as part of a riding model except the OH and H_2O hydrogens for **1**-100 which were not included explicitly in the model but are included in the unit cell contents and all values derived from them. Selected values of the refinement are included in Section S2.7.

Gas Sorption: Measurements were obtained on a Quantachrome Autosorb iQ. Samples were degassed at 423 K for 20 hours prior to analysis, and nitrogen adsorption and desorption isotherms were collected at 77 K up to 1 bar. BET areas were calculated using the BETSI software package.[S3]

Elemental Analysis: CHN data was collected using an Exeter CE440 Elemental Analyser, run in CHN mode with combustion temperature set to 1228 K and reduction temperature set to 923 K.

Scanning Electron Microscopy: Samples were imaged using a Carl Zeiss Sigma variable Pressure Analytical SEM. Powders were deposited onto carbon tabs mounted on aluminium stubs and coated with Pd for 150 seconds using a Polaron SC7640 sputter coater.

Solid-State NMR Spectroscopy: Solid-state NMR spectra were acquired using a Bruker Avance III spectrometer equipped with a 14.1 T wide-bore magnet. ^{13}C MAS NMR spectra were acquired using either direct polarisation or cross polarisation (CP)[S4] from ^{1}H with a 2.5 ms contact pulse (ramped for ^{1}H). TPPM-15[S5] ^{1}H decoupling (with a rf nutation rate of 100 kHz) was applied during the acquisition of all ^{13}C MAS NMR spectra. All MAS NMR spectra were acquired at spinning speeds of 12.5 kHz using a conventional 4 mm HX

Bruker probe, with rf nutation rates of 90-100 kHz for ^{13}C and are referenced relative to TMS (using a secondary reference of l-alanine, 20.5 ppm (**C**H_3)). Lineshape analyses have been fitted using the SOLA package available as part of TopSpin version 4.1.

^{13}C T_1 relaxation experiments were carried out using a saturation recovery experiment on **1**-75*MeOH-as* before and after calcination and indicate maximum T_1 values of 33 s and 400 s for the carbonyl carbon (C=O) signal at 174 ppm, respectively. As a result, recycle intervals of 3 minutes and 40 minutes were used in the direct polarisation experiments to acquire quantitative ^{13}C MAS NMR spectra. The increase in T_1 times following calcination can be attributed to the removal of dynamic solvent from the MOF pores leading to longer relaxation times.

Density Functional Theory (DFT): All DFT calculations were performed using the CP2K code, which uses a mixed Gaussian/plane-wave basis set.[S6] We employed double-ζ polarization quality Gaussian basis sets[S7] and a 400 Ry plane-wave cutoff for the auxiliary grid, in conjunction with the Goedecker-Teter-Hutter pseudopotentials.[S8] All DFT calculations were performed in the Γ-point approximation with sufficiently large supercells (the minimum cell length being >11 Å, and the total number of atoms in each simulation cell ranging between 104 and 256). Total energy calculations and structural optimizations, including both atomic coordinates and cell parameters, were performed under periodic boundary conditions at the DFT level using the PBE exchange and correlation functional,[S9] with Grimme's D3 van der Waals correction (PBE+D3).[S10] A convergence threshold of 1.0×10^{-6} Hartree was used for the self-consistent field cycle, and structural optimizations were considered to have converged when the maximum force on all atoms falls below 4.5×10^{-4} Hartree/Bohr.

S2. Synthesis

MIL-88A(Sc) was synthesised according to our previously reported methods.[S11]

S2.1. Sample Naming Scheme

In this manuscript, MIL-53(Sc)-fum is simplified as **1**. To fully encapsulate the synthesis and processing/activation conditions used, the following naming scheme has been derived:

- Samples of **1** will be given the suffix -100, -75, or-50 to describe the solvent of self-assembly. Samples **1**-100, **1**-75, and **1**-50 therefore correspond to samples crystallised in 100% H_2O, 75% *v/v* H_2O:DMF, and 50% *v/v* H_2O:DMF, in turn.
- Samples of **1** with no additional suffix refer to those which have been isolated from these solvents by centrifugation or filtration without additional drying.
- Samples of **1** that have been dried overnight in a vacuum desiccator from their crystallisation solvent, without any additional treatment, are described as *as-synthesised*, and given the suffix "*as*".
- Samples of **1** that have been *activated* by heating to 423 K under 10^{-4} Torr vacuum for 20 h are given the additional suffix "*act*".
- Samples of **1** that have been solvent exchanged by refluxing overnight in *methanol* or *acetone*, isolated by filtration or centrifugation, then dried overnight in a vacuum desiccator, will be given the additional suffix "*MeOH*" or "*ace*", respectively.
- Samples of **1** that have been solvent exchanged at *room temperature* (RT) overnight in *methanol*, isolated by filtration or centrifugation, then dried overnight in a vacuum desiccator, will be given the additional suffix "*MeOH-RT*".
- When discussing individual crystal structures, the suffixes (*a*), (*b*), and (*c*) from our previously derived[S12] polymorph classification system will be applied.
- DFT optimised simulated structures will contain the suffix "*sim*".
- Suffixes can be combined.

Therefore, as an example, **1**-100*ace-act* would correspond to a sample of **1**, crystallised from 100% H_2O, solvent exchanged by refluxing in acetone, then isolated and activated by heating to 423 K under 10^{-4} Torr vacuum for 20 h.

S2.2. Crystallisation of 1-100 and Characterisation of 1-100*as*

MIL-88A(Sc) (approximately 0.08 g) isolated as the as-synthesised DMF solvate was added to a small scintillation vial and deionised H_2O (10 mL) was added. The mixture was left sealed and undisturbed for 10 days to generate single crystals used for the crystal structure determination of **1**-100, which were used without further purification.

In the crystal structure of **1**-100, the data collection was carried out at the UK National Crystallography Service,[S1] as the sample was highly prone to solvent loss, making it extremely difficult to handle and analyse Only one crystal diffracted suitably to obtain a structure, which showed disorder. The major component of the data by far (~95%) corresponds to the MIL-53(Sc)-fum*(a)* isomer as described in the manuscript. There is an additional component for which a disorder model that satisfies the data somewhat could be produced, but is not chemically sensible, resulting in a minor sub-component that is not a fully connected framework (μ_2-OH units could not be found and the connectivity of Sc ions by fumarate is not correct) and with Sc positions that are too close to one another (Figure S1).

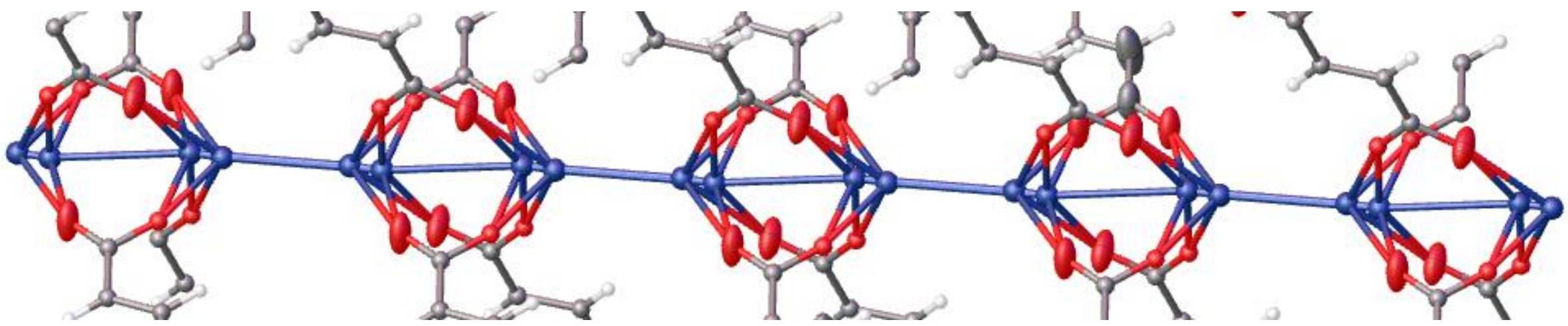

Figure S1. Representation of disconnected Sc units modelled from the data collected for **1**-100, which comprise ~5% of the total intensity. This component was not modelled in the final structure due to it not making chemical sense.

We believe these issues are a consequence of trying to apply a disorder model to an issue that does not originate from disorder, but from the fact that the dataset comes from multiple crystals, or cracked crystals. Given that this very minor component represents ~5% of the total data for the framework, and could not be modelled with any chemical sensibility, it was left as unmodelled in the final refinements of **1**-100, which has resulted in a slightly higher than desirable R_1 and residual electron density peaks (full crystallographic data for all structures are provided for comparison in Section S2.7).

Given that non-linear linkers like fumarate lead to the possibility of correlated disorder, as seen in DUT-8(Ni) where the linker is naphthalene-2,6-dicarboxylate,[S13] precession images were synthesised. Some diffuse scattering in the *0kl* plane is evident (Figure S2), which is likely due to the heavily disordered and mobile water molecules found in the diamondoid channel which runs down the crystallographic *a* axis.

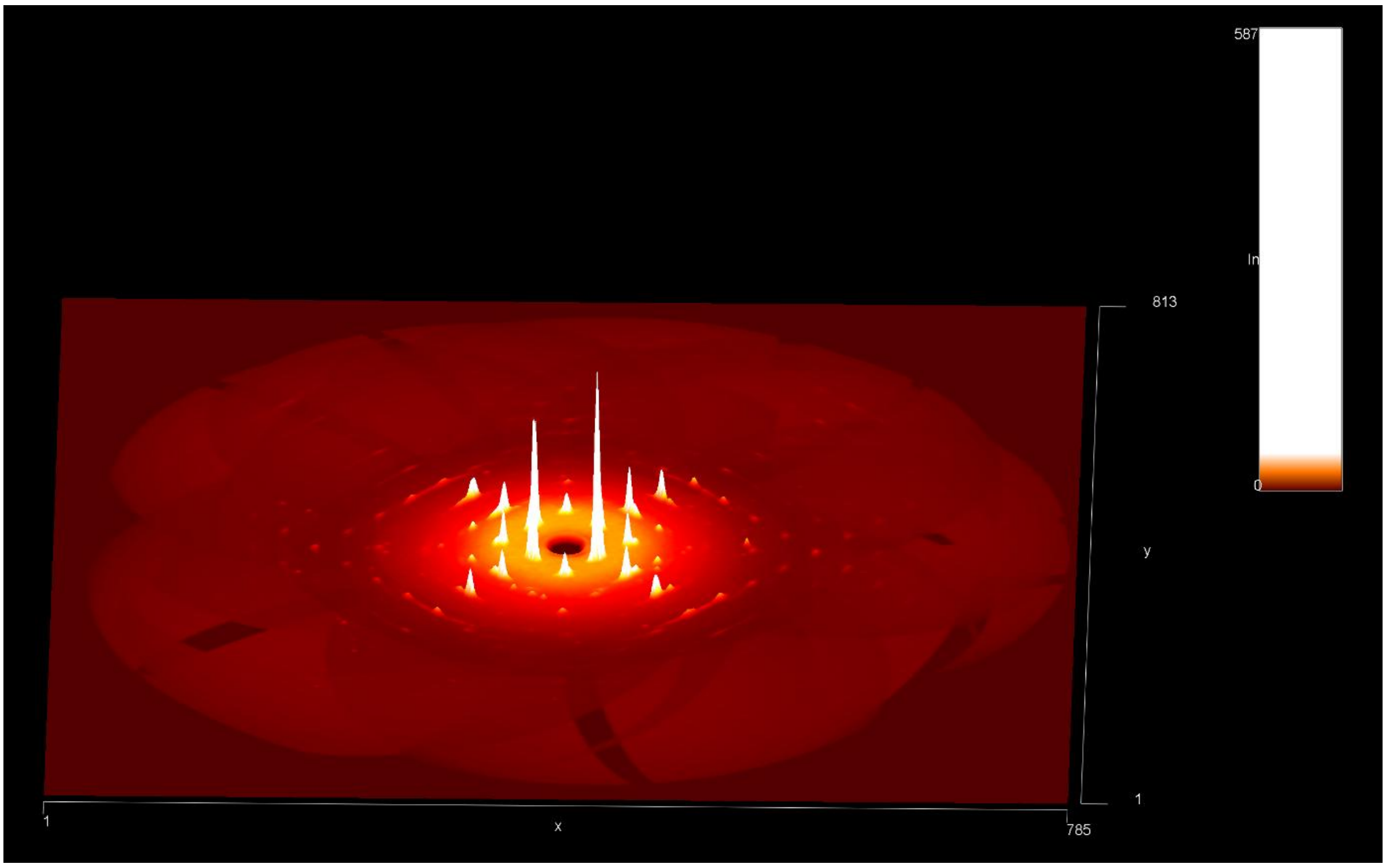

Figure S2. Precession image in the *0kl* plane for **1**-100.

Despite their heavy disorder, it was possible to model some partially occupied water molecules in the pores of **1**-100. Two CIFs are, therefore, provided for **1**-100: one

contains modelled water where the occupancies are substantial (**1**-100; R_1 = 0.140; wR_2 = 0.372, 3 H_2O per Sc), and one has a solvent mask applied (**1**-100 mask; R_1 = 0.139; wR_2 = 0.385, 24 electrons, ~2.4 H_2O per Sc). Full crystallographic data are provided in Section S2.7.

Bulk samples of **1**-100 were prepared in the same way as for isolation of the single crystal sample, and small experiments combined to yield scaled up samples. For time dependent studies, repeat experiments were carried out, and the solids collected by filtration or centrifugation from the mother solution at varying time points. The resultant products were analysed directly by powder X-ray diffraction without further drying (Figure S3).

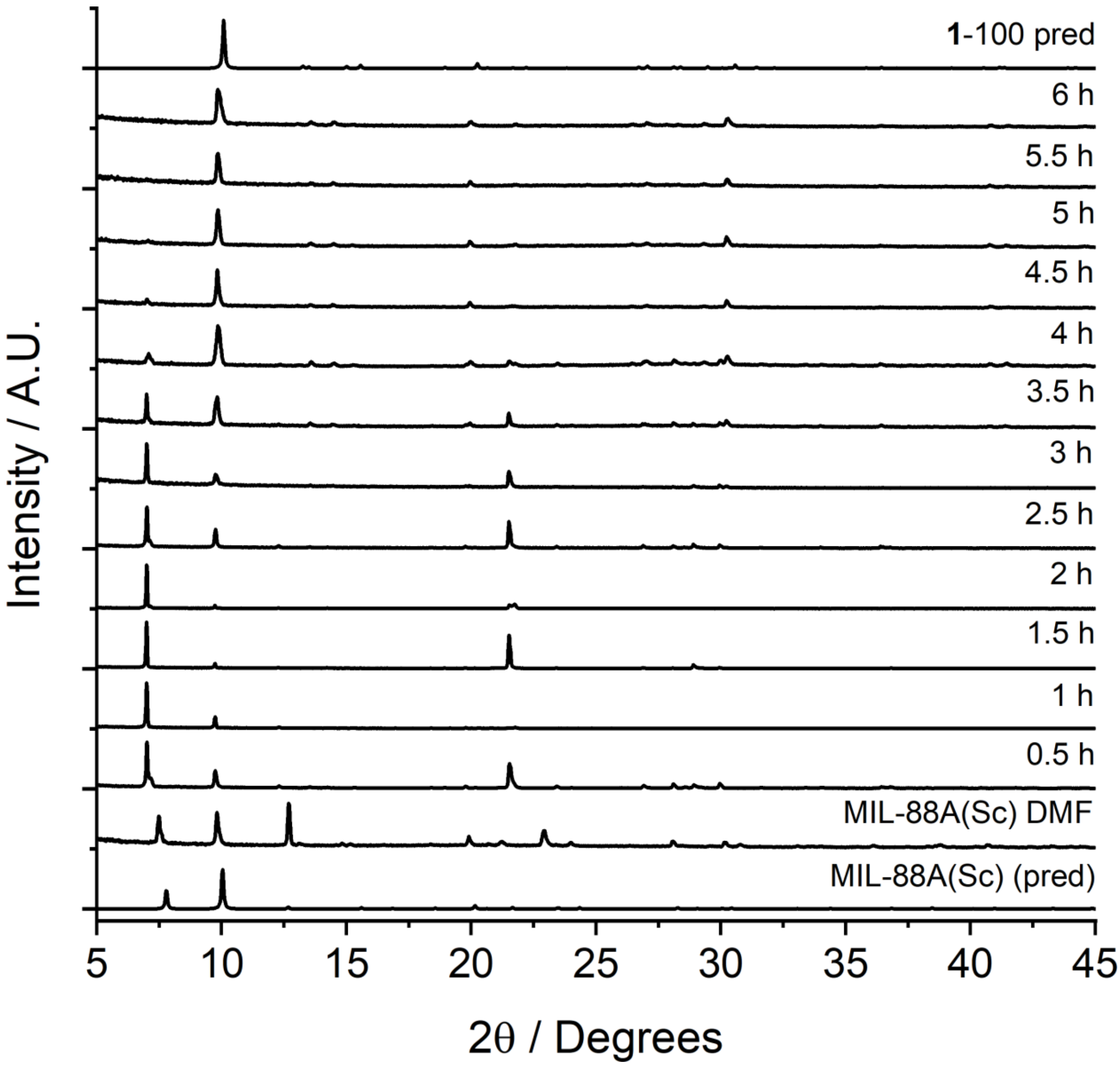

Figure S3. Stacked partial powder X-ray diffractograms showing the transformation of MIL-88A(Sc) as the DMF solvate, by immersion in 100% H_2O, to **1**-100 over a time period of 6 hours. Additional comparison to patterns predicted from the crystal structures of MIL-88A(Sc) and for **1**-100 is provided.

Samples of **1**-100*as* were generated by the above procedure, being kept in solvent for 10 days to ensure complete transformation and dried overnight in a vacuum desiccator. Powder X-ray diffraction analysis of pristine **1**-100 compared to **1**-100*as* (Figure S4) shows that (i) the synthesised material corresponds to phase pure MIL-53(Sc)-fum, and (ii) the sample exhibits some flexibility: the shift of major peaks to higher 2θ angles suggests the framework is closing slightly upon solvent loss.

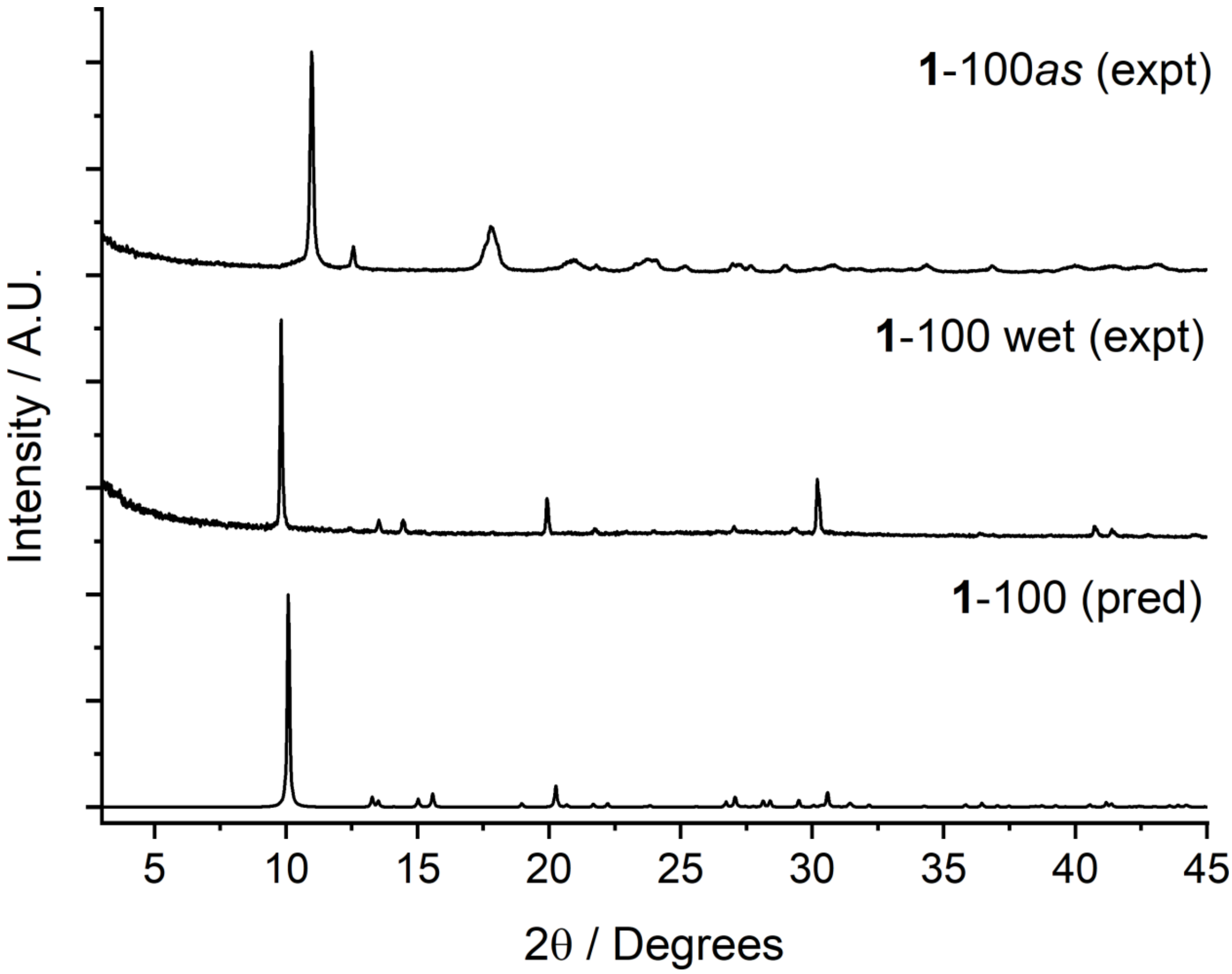

Figure S4. Stacked partial powder X-ray diffractograms of **1**-100 (collected wet from water), and **1**-100*as* (dried directly from water), compared with the diffractogram predicted from the single crystal structure of **1**-100.

^{1}H NMR spectroscopic analysis of acid-digested (DMSO-d_6/D_2SO_4) **1**-100*as* suggests a formula of $[Sc(OH)(C_4H_2O_4)]\cdot(C_3H_7NO)_{0.5}$ based on the integration of resonances assigned to protons of DMF at δ = 7.9, 2.8 and 2.7 ppm compared to the resonance for the fumarate protons at δ = 6.6 ppm (Figure S5).

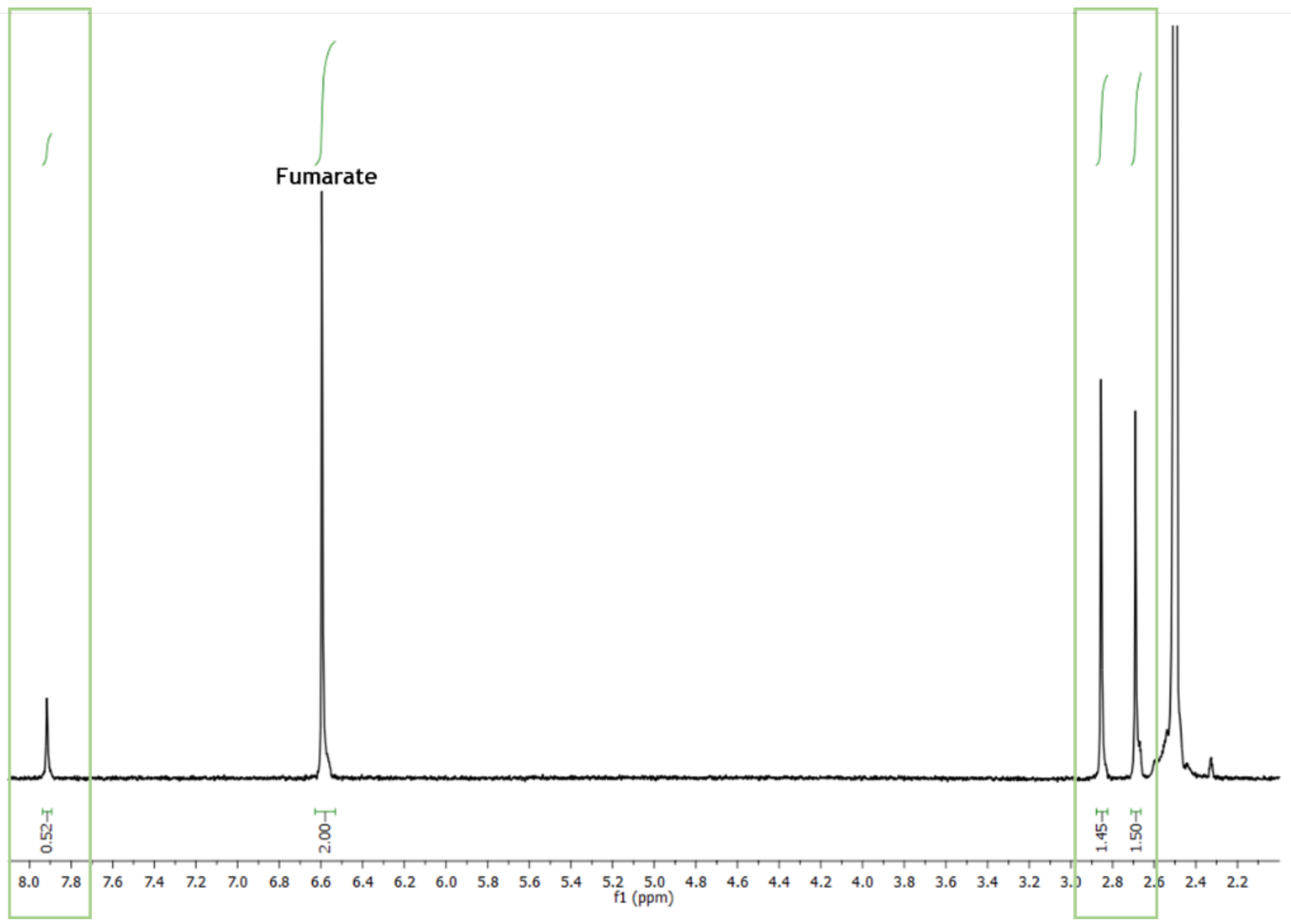

Figure S5. Partial ^{1}H NMR spectrum of an acid-digested (DMSO-d_6/D_2SO_4) sample of **1**-100*as*. Resonances assigned to protons of DMF are highlighted in green boxes; the resonance assigned to fumarate protons is labelled. Comparison of the integral intensities gives a fumarate:DMF ratio of 1:0.5.

Elemental analysis supports the ^{1}H NMR spectroscopy data and confirms the presence of residual DMF in the bulk sample; a nitrogen content of 3.08% represents approximately 0.5 DMF molecules remaining in the framework per Sc unit in the formula $[Sc(OH)(C_4H_2O_4)]\cdot(C_3H_7NO)_{0.5}$.

Experimental results: C, 31.92%; H, 3.00%; N, 3.08%.
Calculated results: C, 31.08%; H, 3.08%; N, 3.30%.

The DMF content in **1**-100*as*, which is likely to have come from residual DMF in MIL-88A(Sc), is also accounted for in the TGA profile. An initial mass loss of 14% between 120-180 °C is indicative of this solvent loss (expected = 15.5% based on 0.5 DMF),

followed by a large mass loss at 400 °C representing the degradation of the linker and subsequently, the entire framework (Figure S6). The resultant residual mass is 32.0% wt and this matches closely with an expected residual mass of 32.4% wt, assuming complete conversion to Sc_2O_3, based on the above formula. In previously reported MIL-53 frameworks, mass loss below 100 °C is assigned to pore-bound water molecules,[S14] however, this is not evident in the profile for **1**-100*as* and would result in extreme discrepancies in both the residual weight as shown by TGA and the carbon content, as determined by microanalysis.

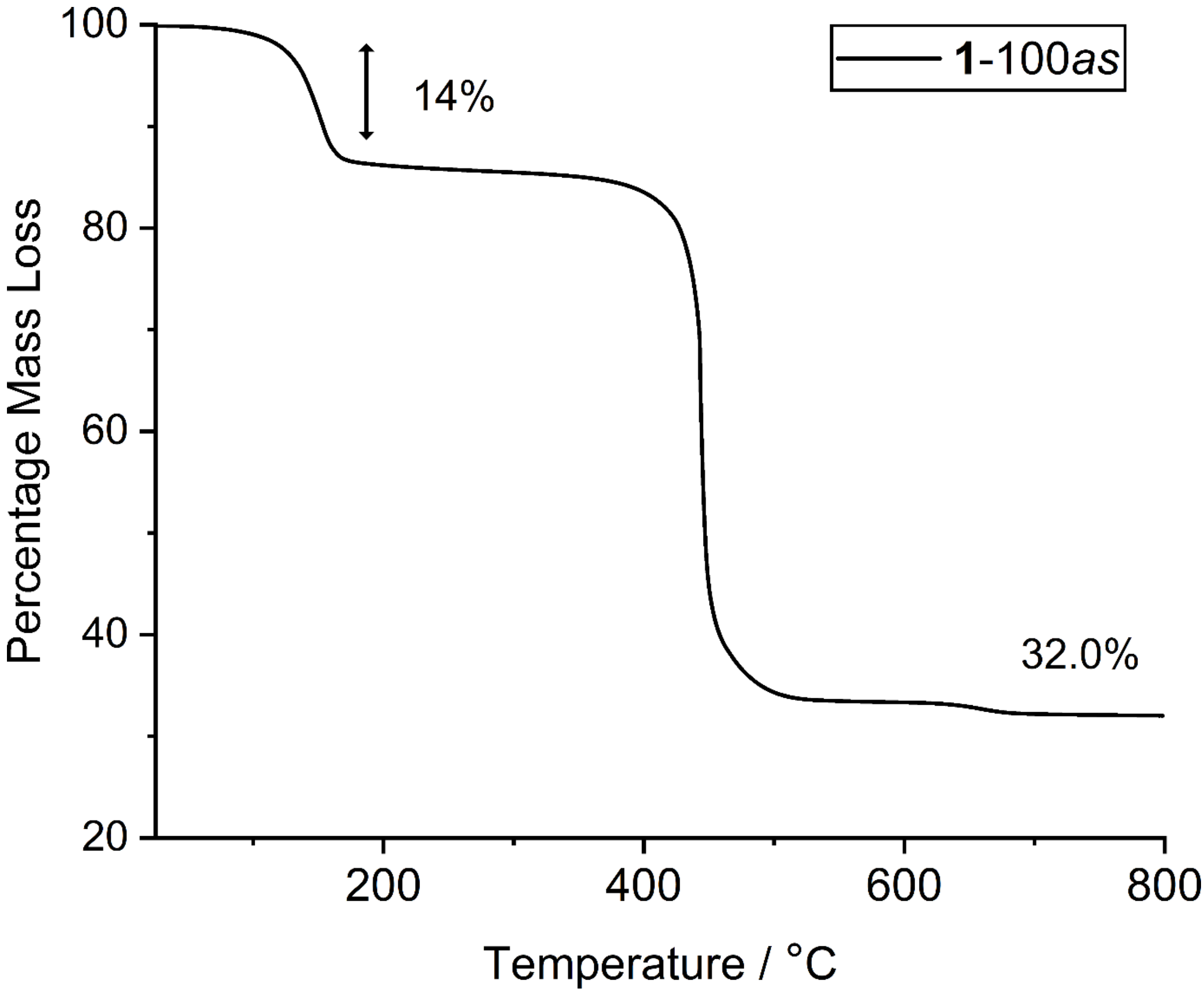

Figure S6. TGA profile of **1**-100*as* collected between 25-800 °C in air.

S2.3. Crystallisation of 1-75 and Characterisation of 1-75*as*

MIL-88A(Sc) (approximately 0.08 g) isolated as the as-synthesised DMF solvate was added to a small scintillation vial and a mixture of deionised H_2O (7.5 mL) and DMF (2.5 mL), corresponding to 75% *v/v* H_2O in DMF, was added. The mixture was left sealed and undisturbed for 10 days to generate single crystals used for the crystal structure determination of **1**-75, which were used without further purification.

As described in the main manuscript, the crystal structure of **1**-75 includes three disordered components of the fumarate linker and two orientationally disordered sites of the Sc SBU, which are visualised in Figure S7.

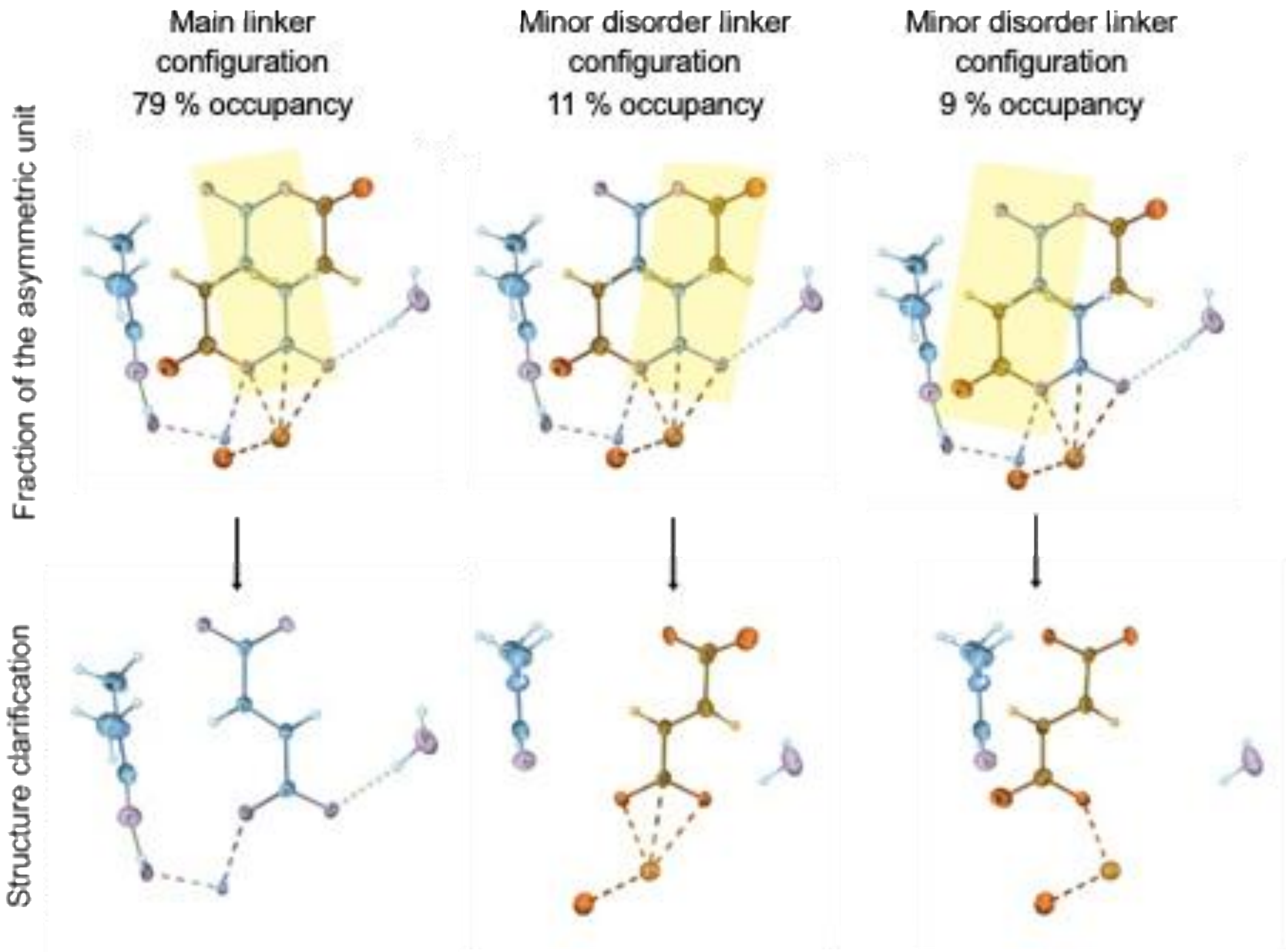

Figure S7. Images of the different disorder components in the crystal structure of **1**-75.

Whilst it has not been possible to definitively determine the origin of the disorder in the structure, we have been able to ascertain that correlated disorder is not present due to the lack of diffuse scattering in procession images. Full crystallographic data are provided in Section S2.7.

Bulk samples of **1**-75 were prepared in the same way as for isolation of the single crystal sample, and small experiments combined to yield scaled up samples. For time dependent studies, repeat experiments were carried out, and the solids collected by filtration or centrifugation from the mother solution at varying time points. The resultant products were analysed directly by powder X-ray diffraction without further drying.

PXRD was used to confirm both the phase-purity and crystallinity of the framework. A timed experiment was carried out, taking samples out of the transformation solution at intervals over a period of 10 days. Where **1**-100 was formed within 5 hours, the deliberate addition of 25% (*v/v*) DMF to the transformation mixture drastically slows down the reaction. A new phase is observed after 24 hours, however, with a very low relative intensity. After 48 hours, the initial peak corresponding to MIL-88A(Sc) disappears and there appears to be a majority of **1**-75 formed (Figure S8).

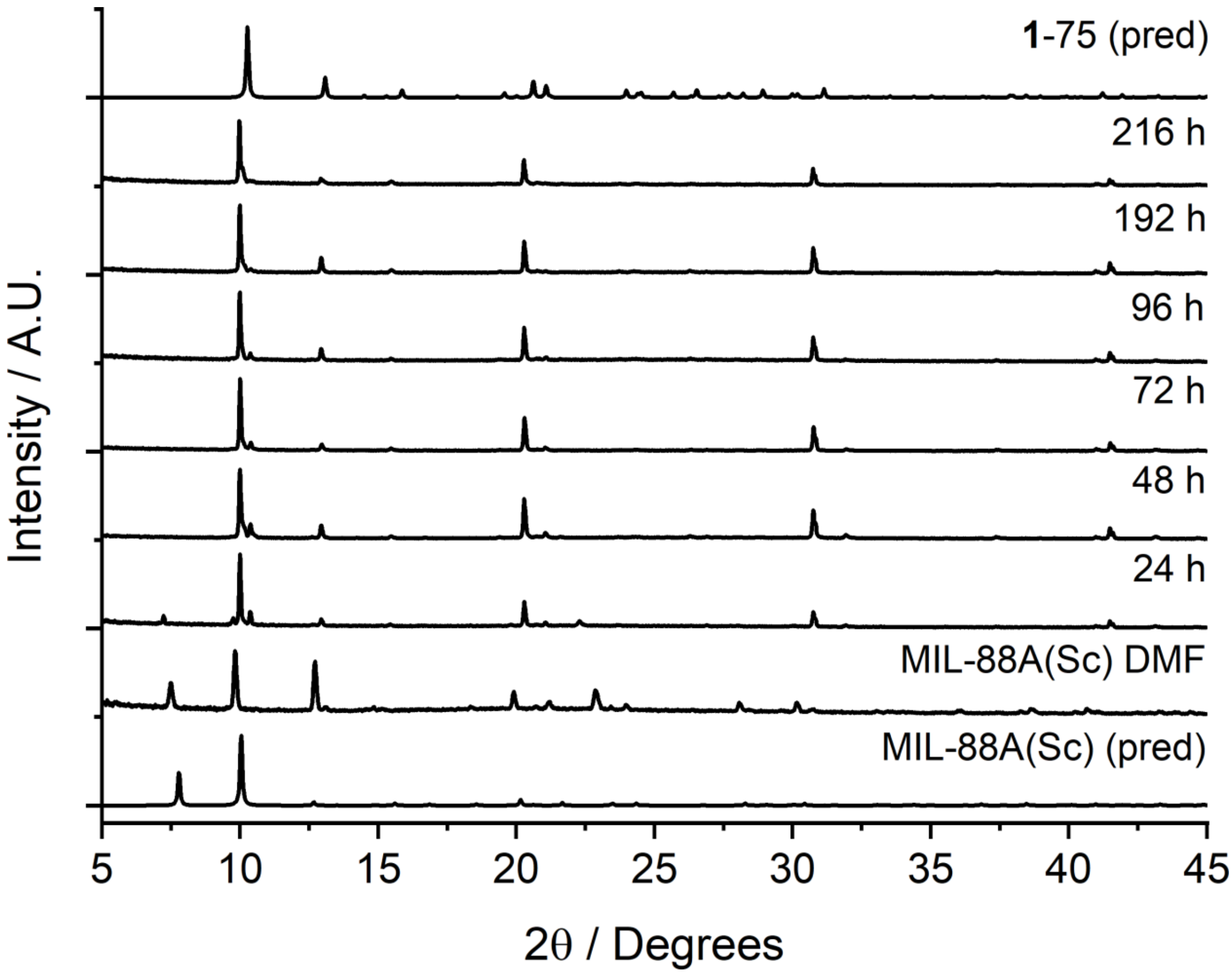

Figure S8. Stacked partial powder X-ray diffractograms showing the transformation of MIL-88A(Sc), by immersion in 75% (*v/v*) H_2O in DMF, to **1**-75 over a time period of 8 days. Additional comparison to patterns predicted from the crystal structures of MIL-88A(Sc) and **1**-75 is provided.

Samples of **1**-75*as* were generated by the above procedure, being immersed in the solvent mixture for 10 days, and dried overnight in a vacuum desiccator. Powder X-ray diffraction analysis of pristine **1**-75 compared to **1**-75*as* shows that (i) the synthesised material corresponds to phase pure MIL-53(Sc)-fum, and (ii) the sample exhibits minor flexibility; the shift of major peaks to higher 2θ angles suggests the framework is closing slightly upon solvent loss (Figure S9).

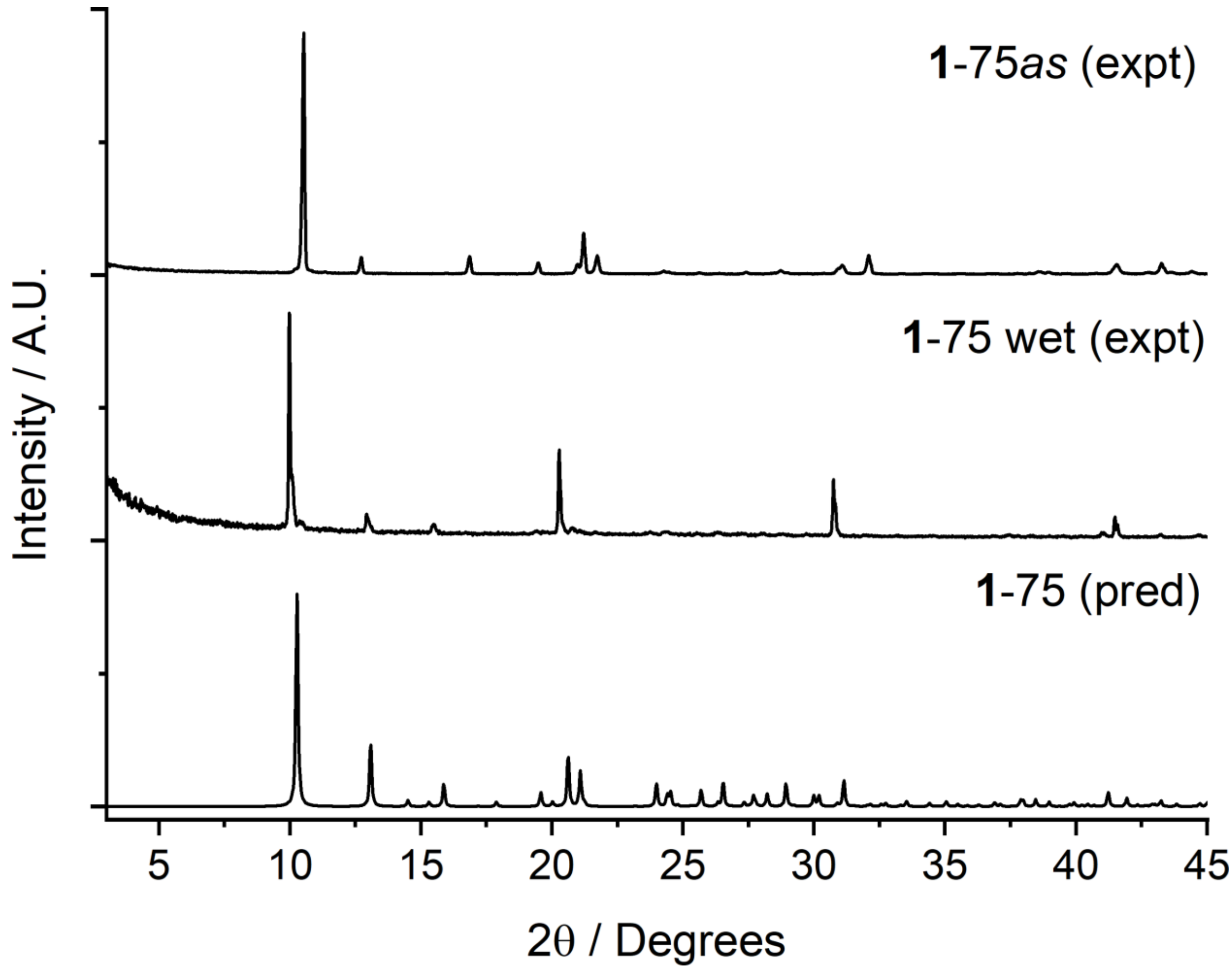

Figure S9. Stacked partial powder X-ray diffractograms of **1**-75 (collected wet from DMF/water), and **1**-75*as* (dried directly), compared with the diffractogram predicted from the single crystal structure of **1**-75.

The slight differences in peak intensity can be attributed to preferred orientation, evidenced by comparison with a diffractogram predicted from the crystal structure of **1**-75 with a March Dollase parameter of 1.5 applied in the 100 direction (Figure S10).

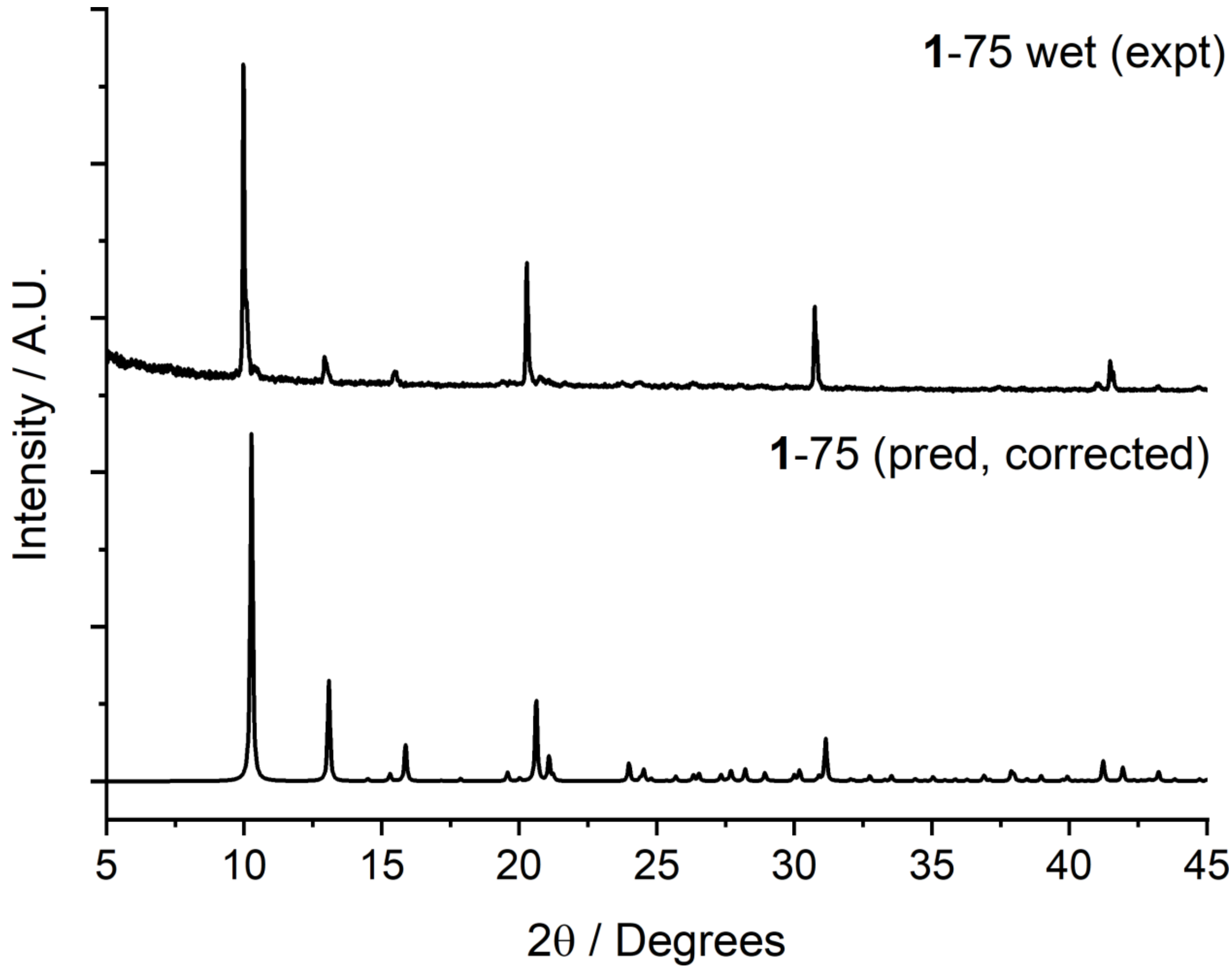

Figure S10. Stacked partial powder X-ray diffractograms of **1**-75 (collected wet from DMF/water), compared with the diffractogram predicted from the single crystal structure of **1**-75 corrected for preferred orientation with a March-Dollase parameter of 1.5 applied in the 100 direction.

^{1}H NMR spectroscopy of acid digested **1**-75*as* suggests the presence of 1 mole of DMF per fumarate ligand (Figure S11) – integration of the resonances at 2.86 and 2.70 ppm to 3H equates to one linker (2H) – leaving a proposed formula of $[Sc(OH)(C_4H_2O_4)]\cdot(C_3H_7NO)$. This would correlate with evaporation of water, but not DMF, from the material (the crystallographic formula for **1**-75 has a 1:1:1 Sc:DMF:H_2O ratio).

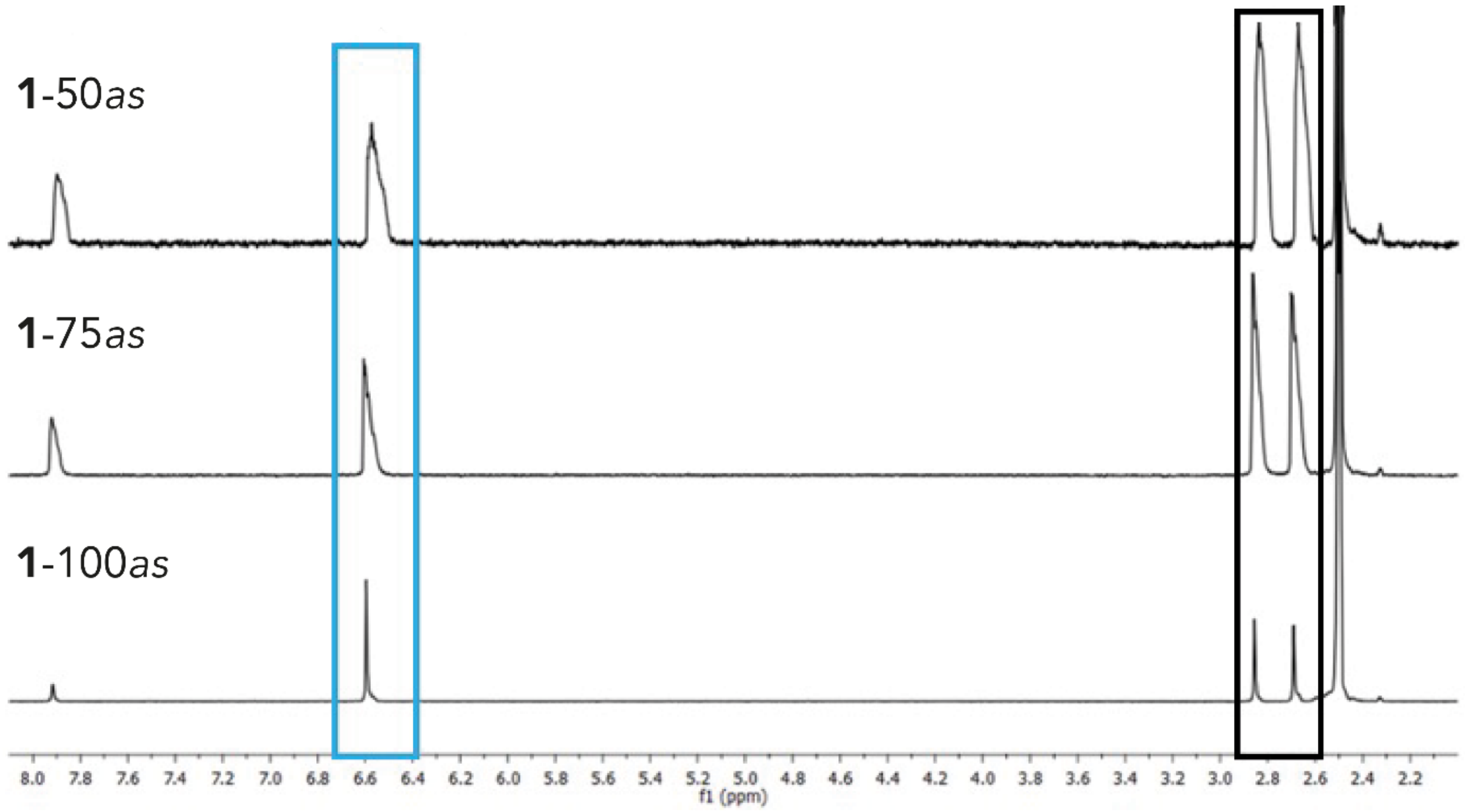

Figure S11. Stacked partial ^{1}H NMR spectra of acid-digested (DMSO-d_6/D_2SO_4) samples of the as-synthesised samples, dried at room temperature, presented for comparison. Resonances assigned to protons of DMF are highlighted in black boxes; the resonance assigned to fumarate protons is labelled. Broadening of some spectra is thought to be due to unfilterable particulates.

Elemental analysis strongly supports the NMR spectroscopic data, based on the formula $[Sc(OH)(C_4H_2O_4)]\cdot(C_3H_7NO)$.

Experimental results: C, 33.65%; H, 4.03%; N, 5.49%.

Calculated results: C, 33.75%; H, 4.05%; N, 5.62%.

TGA measurements were carried out from room temperature to 800 °C, in increments of 10°C min^{-1} in air, and the TGA profile is in good agreement with one mole of DMF per Sc unit, showing a mass loss of 28% in the region between 100°C to 170 °C (Figure S12) compared to an expected 29.3% for DMF. **1**-75*as* shows good thermal stability to 400 °C and a residual mass loss of 27.8% wt agrees with the expected residual mass (27.7% wt) based on this formula being converted entirely to Sc_2O_3.

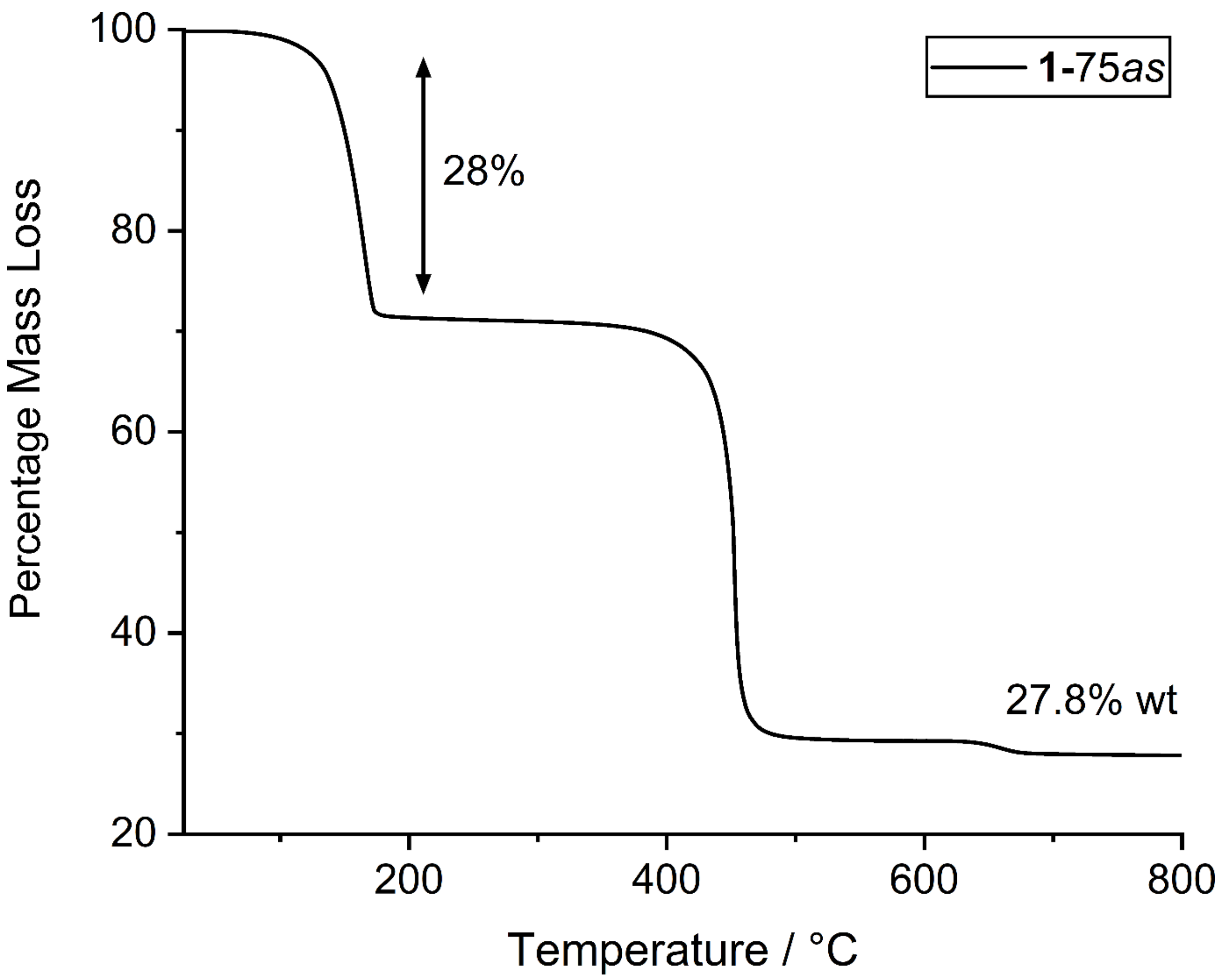

Figure S12. TGA profile of **1**-75*as* collected between 25-800 °C in air.

S2.4. Crystallisation of 1-50 and Characterisation of 1-50*as*

MIL-88A(Sc) (approximately 0.08 g) isolated as the as-synthesised DMF solvate was added to a small scintillation vial and a mixture of deionised H_2O (5 mL) and DMF (5 mL), corresponding to 50% *v/v* H_2O in DMF, was added. Single crystals suitable for analysis by X-ray diffraction could not be obtained, but bulk samples of **1**-50 were analysed directly by powder X-ray diffraction.

For time dependent studies, repeat experiments were carried out, and the solids collected by filtration or centrifugation from the mother solution at varying time points.

The resultant products were analysed directly by powder X-ray diffraction without further drying (Figure S13).

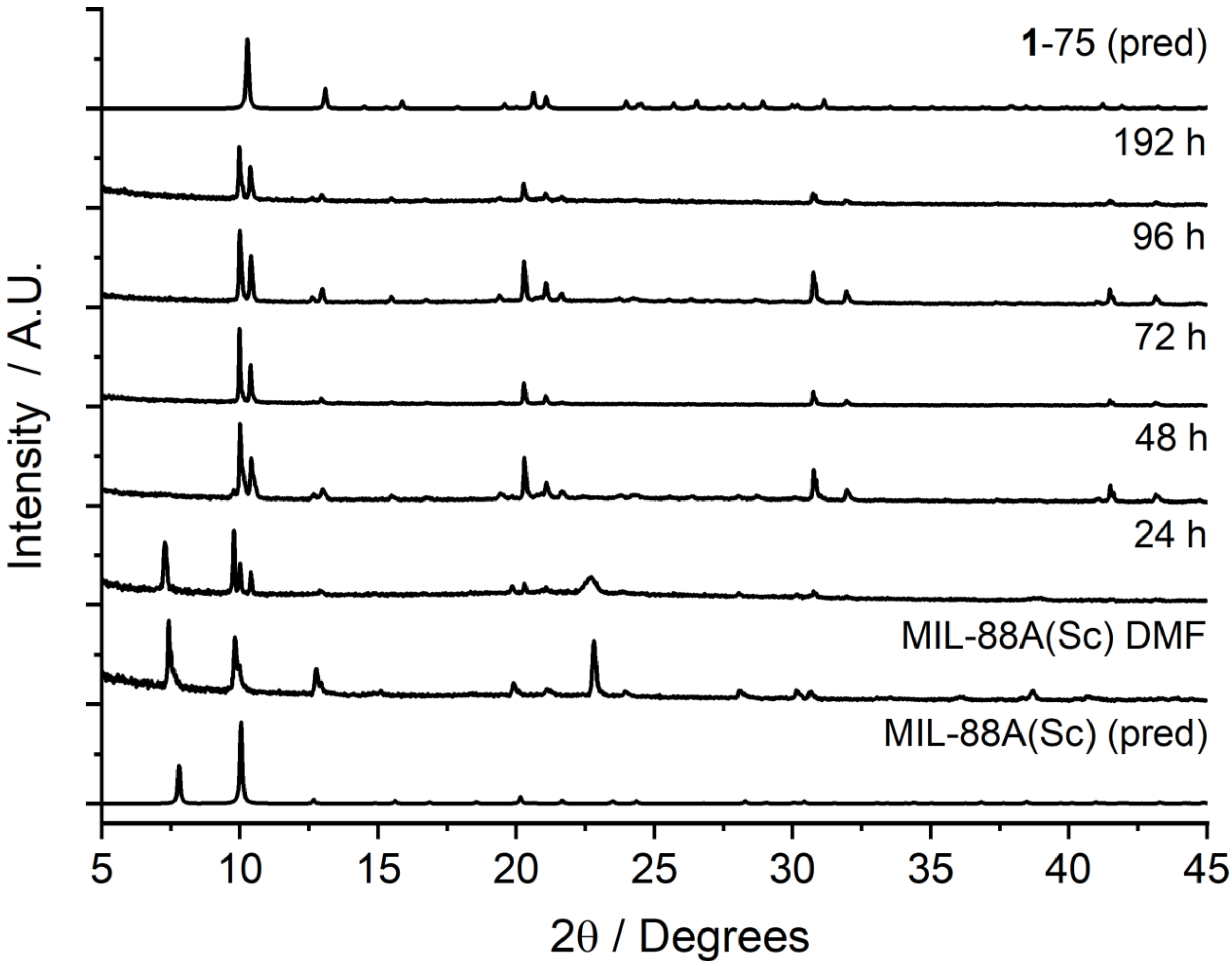

Figure S13. Stacked partial powder X-ray diffractograms showing the transformation of MIL-88A(Sc), by immersion in 50% (*v/v*) H_2O in DMF, to **1**-50 over a time period of 8 days. Additional comparison to patterns predicted from the crystal structures of MIL-88A(Sc) and for **1**-75 is provided.

Similar to **1**-75, a clear transformation is observed between 24 and 48 hours in the formation of **1**-50, however, in this case an extra Bragg peak at 2θ ~ 10.4° is prevalent in the wet samples for the duration of the timed experiment. After 10 days, when **1**-50 is dried directly from the DMF/H_2O mixture to yield **1**-50*as*, the pattern shifts slightly to a higher 2θ angle, indicating a slight closure of the framework to a more narrow-pore form. The collected diffractogram for **1**-50*as* is essentially identical to **1**-75*as*, indicating that they close to the same extent when dried at room temperature (Figure S14).

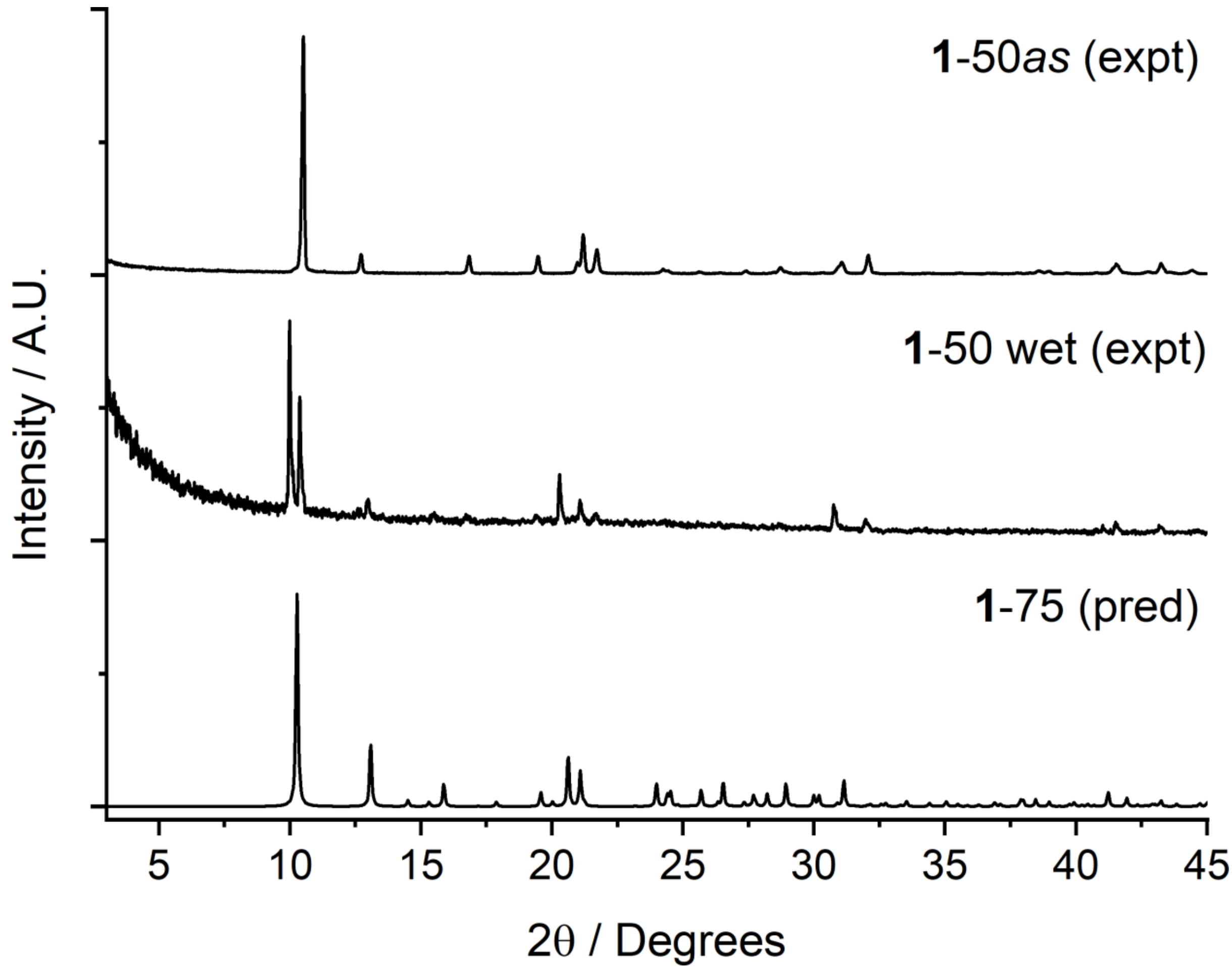

Figure S14. Stacked partial powder X-ray diffractograms of **1**-50 (collected wet from DMF/water), and **1**-50*as* (dried directly), compared with the diffractogram predicted from the single crystal structure of **1**-75.

Similar to **1**-75*as*, ^{1}H NMR spectroscopy of acid digested **1**-50*as* (Figure S11) suggests a formula of $[Sc(OH)(C_4H_2O_4)]\cdot(C_3H_7NO)$ based on the relative integral ratios.

Elemental analysis results also support the composition being $[Sc(OH)(C_4H_2O_4)]\cdot(C_3H_7NO)$:

Experimental results: C, 33.70%; H, 4.05%; N, 5.53%.

Calculated results: C, 33.75%; H, 4.05%; N, 5.62%.

Following TGA experiments in air to 800 °C, an experimental Sc_2O_3 residue of 27.7% wt perfectly matches the expected residue of 27.7% wt based on this formula. The loss of DMF is again evident in the TGA profile below 200 °C, whereby approximately 29% mass is lost, corroborating with an expected mass loss of 29.3% for one mole of DMF per Sc unit (Figure S15). Overall, both the structural and composition data suggest that **1**-75*as* and **1**-50*as* are essentially identical, but bear slight differences to **1**-100*as*. A comparison of the three samples is provided in Section S2.6.

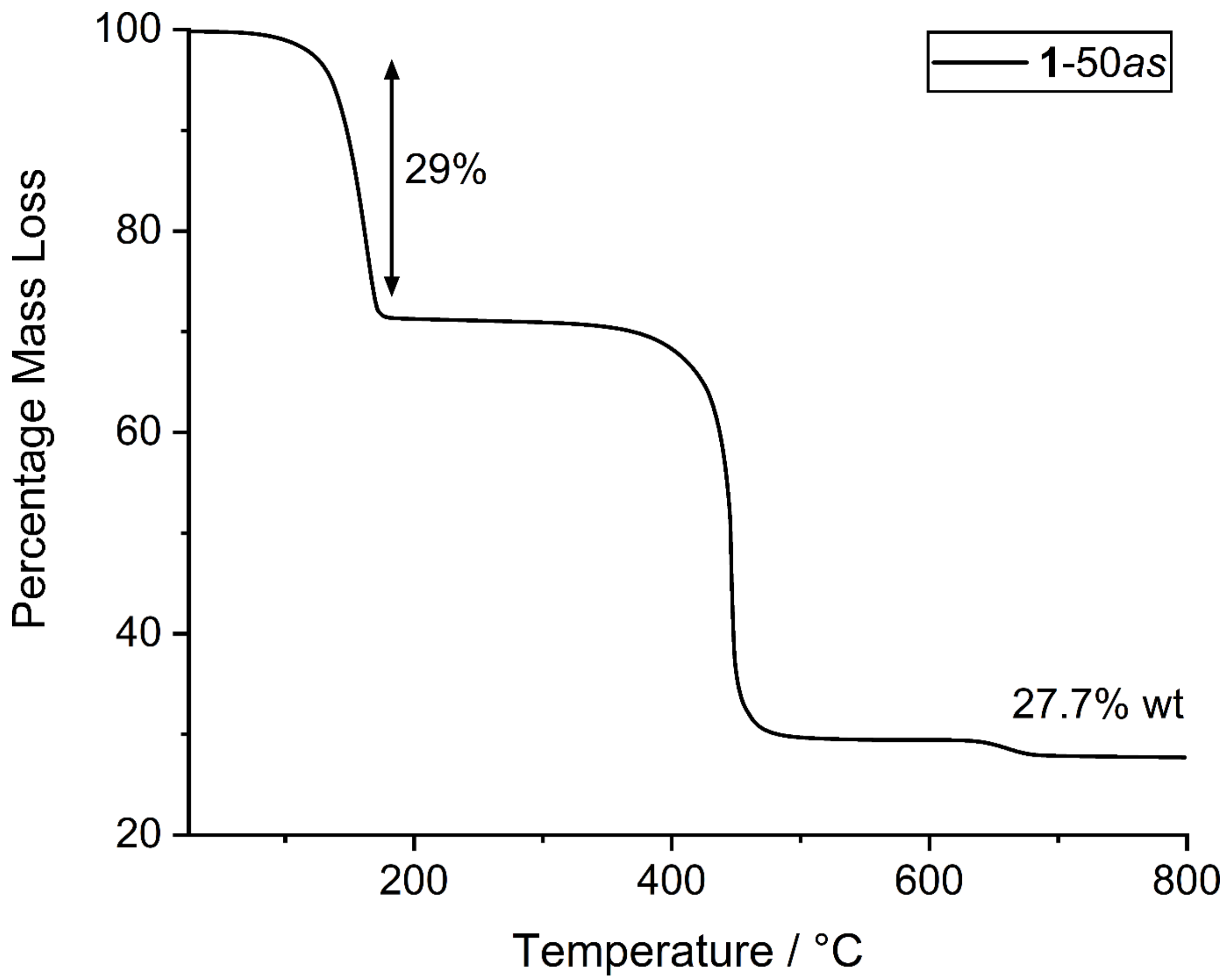

Figure S15. TGA profile of **1**-50*as* collected between 25-800 °C in air.

S2.5. Crystallisation of 1-25

MIL-88A(Sc) (approximately 0.08 g) isolated as the as-synthesised DMF solvate was added to a small scintillation vial and a mixture of deionised H_2O (2.5 mL) and DMF (7.5 mL), corresponding to 25% *v/v* H_2O in DMF, was added. Single crystals suitable for analysis by X-ray diffraction could not be obtained.

For time dependent studies, repeat experiments were carried out, and the solids collected by filtration or centrifugation from the mother solution at varying time points. The resultant products were analysed directly by powder X-ray diffraction without further drying.

Results were inconsistent across different samples. The diffractograms obtained after 9 days suggested incomplete conversion, both in the wet and dried (as-synthesised) states, with additional peaks that may correspond to MIL-88A(Sc). As such, these conditions were not pursued further (Figure S16).

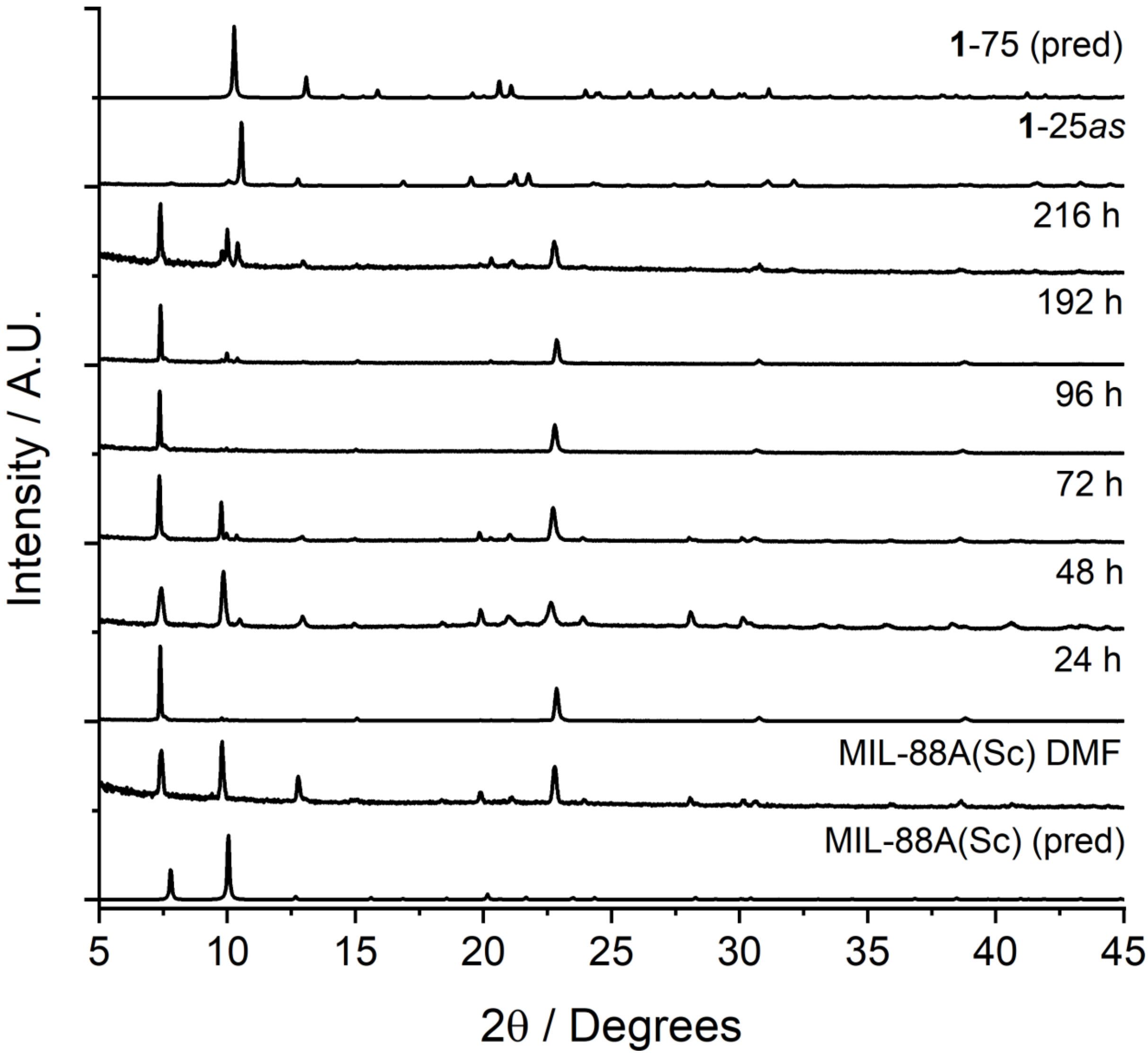

Figure S16. Stacked partial powder X-ray diffractograms showing the partial transformation of MIL-88A(Sc), by immersion in 25% (*v/v*) H_2O in DMF, to **1**-25 over a time period of 9 days. The diffractogram for **1**-25*as*, isolated after drying overnight in a vacuum desiccator, predominantly shows peaks for the MIL-53(Sc)-fum phase but also additional peaks corresponding to MIL-88A(Sc). Additional comparison to patterns predicted from the crystal structures of MIL-88A(Sc) and **1**-75 is provided.

S2.6. Comparison of As-Synthesised Samples

Powder X-ray diffractograms of freshly crystallised samples (**1**-100, **1**-75, and **1**-50; Figure S17) were compared with those that had been isolated and dried in a vacuum

desiccator overnight (**1**-100*as*, **1**-75*as*, and **1**-50*as*; Figure S18). The data show that **1**-100*as* adopts a more closed pore form than **1**-75*as* and **1**-50*as*, likely due to **1**-100*as* containing less DMF than the other samples.

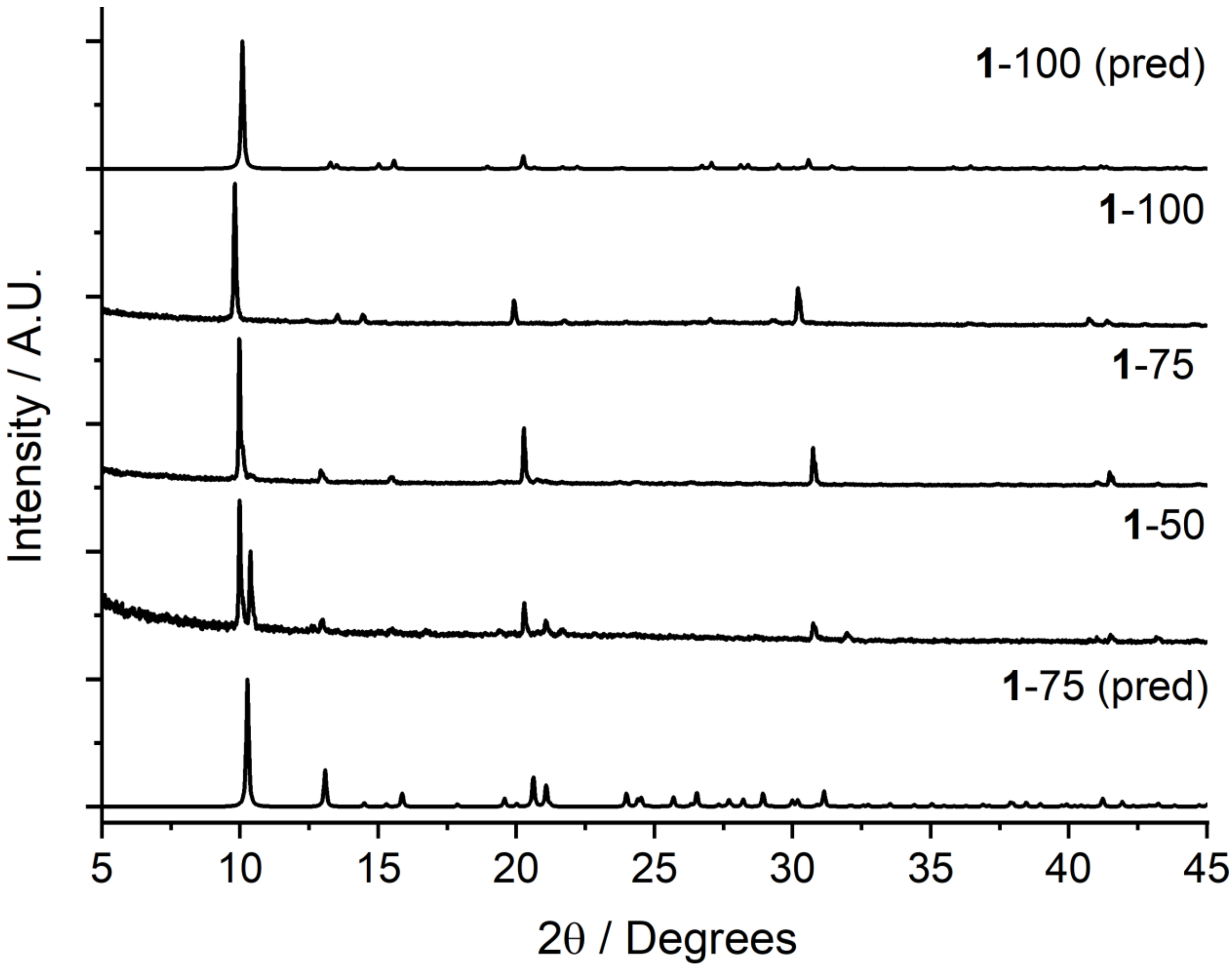

Figure S17. Stacked partial powder X-ray diffractograms of freshly prepared **1**-100, **1**-75, and **1**-50 in comparison to those predicted from the crystal structures of **1**-100 and **1**-75.

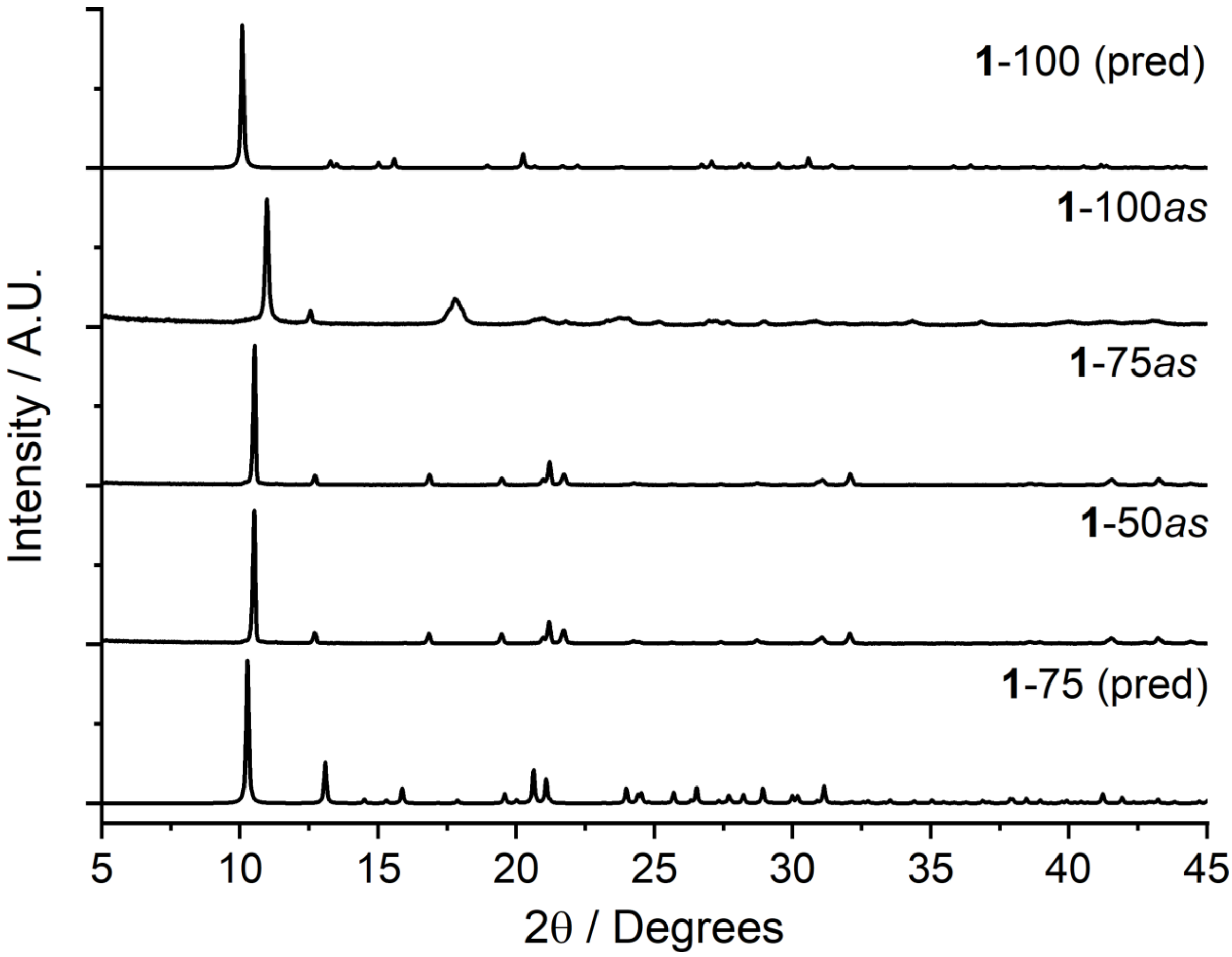

Figure S18. Stacked partial powder X-ray diffractograms of as-synthesised **1**-100*as*, **1**-75*as*, and **1**-50*as* in comparison to those predicted from the crystal structures of **1**-100 and **1**-75.

Comparison of the TGA traces of the as synthesised samples visualises the lower residual solvent content in **1**-100*as* compared to **1-**75*as*, and **1-**50*as* (Figure S19).

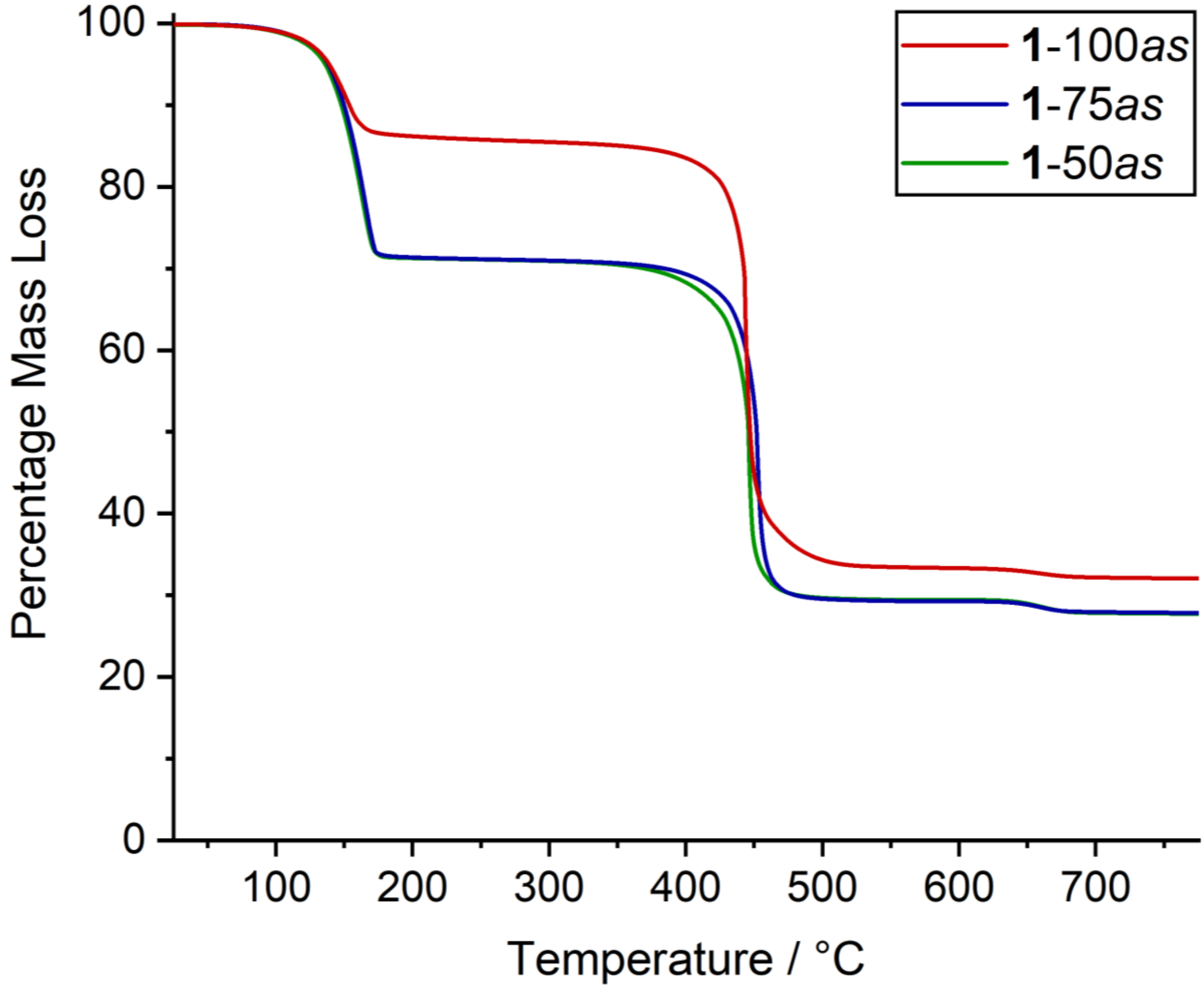

Figure S19. Thermogravimetric analysis traces of as-synthesised **1**-100*as*, **1**-75*as*, and **1**-50*as*, showing the greater retention of solvent in as-synthesised **1**-75*as*, and **1**-50*as*.

The proposed formulae of **1-**100*as*, **1-**75*as*, and **1-**50*as*, alongside data for elemental analysis and TGA, are given in Table S1. These data clearly indicate the similarities between **1-**75*as* and **1-**50*as*.

Table S1. Table to summarise the proposed formulae for each of the samples of **1** based on ^{1}H NMR spectroscopy, TGA and elemental analysis data. Data calculated from the proposed formulae are provided in brackets below the experimental values.

	Proposed Formula	Elemental Analysis			TGA Residue
		C	H	N	
**1-**100*as*	$[Sc(OH)(C_4H_2O_4)]\cdot(C_3H_7NO)_{0.5}$	31.92% (31.08%)	3.00% (3.08%)	3.08% (3.30%)	32.0% (32.4%)
**1-**75*as*	$[Sc(OH)(C_4H_2O_4)]\cdot(C_3H_7NO)$	33.65% (33.75%)	4.03% (4.05%)	5.49% (5.62%)	27.8% (27.7%)
**1-**50*as*	$[Sc(OH)(C_4H_2O_4)]\cdot(C_3H_7NO)$	33.70% (33.75%)	4.05% (4.05%)	5.53% (5.62%)	27.7% (27.7%)

S2.7. Comparison of Crystallographic Data

A comparison of pertinent crystallographic data for all known MIL-53(M)-fum structures are provided in Table S2. Full crystallographic data for **1**-100 (as well as the CIF with solvent mask applied: **1**-100 mask) and **1**-75 are provided in Table S3.

Table S2. Comparison of pertinent crystallographic data for MIL-53(M)-fum crystal structures.

M	SG	*a* / Å	*b* / Å	*c* / Å	*β* / °	*V* / Å^3	Isomer	REF
Al[a]	$P2_1/c$	6.842(3)	12.088(2)	14.21(1)	122.55(6)	990.432	*(a)*	S15
Ga[a]	$P2_1/c$	7.159(1)	12.283(1)	14.461(2)	122.87(1)	1068.0(2)	*(a)*	S16
Ga[a]	*I*2/a	6.840(1)	12.133(2)	12.315(2)	94.855(17)	1018.41	*(b)*	S17
In	*Pnma*	7.307(2)	10.626(3)	13.915(5)	90	1080.42	*(c)*	S12
Sc	*I*2/*m*	7.2742(9)	13.1041(19)	11.979(2)	100.245(13)	1123.7(3)	*(a)*	herein
Sc	$P2_12_12_1$	7.2905(2)	11.1622(3)	13.5287(4)	90	1100.94(5)	*(c)*	herein

[a]Derived from powder X-ray diffraction data.

Table S3. Crystallographic data tables for **1**-100, **1**-100 mask, and **1**-75.

Compound	**1**-100	**1**-100 mask	**1**-75
Empirical formula	$Sc(OH)(C_4H_2O_4)\cdot 3H2O$	$Sc(OH)(C_4H_2O_4)$	$Sc(OH)(C_4H_2O_4)\cdot 0.85C_3H_7NO\cdot 0.841H_2O$
Formula weight	230.07	176.02	253.12
Temperature (K)	100(2)		100(2)
Wavelength (Å)	1.54178		1.54178
Crystal system	*monoclinic*		*orthorhombic*
Space group	*I2/m*		$P2_12_12_1$
Unit cell dimensions (Å)	*a* = 7.2742(9)<br>*b* = 13.1041(19)<br>*c* = 11.979(2)		*a* = 7.2905(2)<br>*b* = 11.1622(3)<br>*c* = 13.5287(4)
Volume ($Å^3$)	1123.7(3)		1100.94(5)
Z	4		4
Density (g cm^{-1})	1.489	1.035	1.527
Absorption coefficient (mm^{-1})	6.691	5.475	5.914
F(000)	472	352	521
Crystal size (mm)	0.12 × 0.02 × 0.015		0.1×0.07×0.07
θ range (°)	5.046 to 67.679		5.137 to 67.679
Index ranges	$-8 \leq h \leq 8, -14 \leq k \leq 15, -13 \leq l \leq 14$		$-8 \leq h \leq 9, -13 \leq k \leq 11, -17 \leq l \leq 16$
Reflections collected	7204		10029
Independent reflections	1069 [R_{int}= 0.1418]		2138 [R_{int} = 0.0526]
Completeness	0.996		0.996 (θ = 67.679°)
Data / restraints / parameters	1069 / 43 / 86	1069 / 43 / 51	2138 / 45 / 220
S (GOF)	1.500	1.543	1.052
R_1, wR_2 [$I > 2\sigma(I)$]	0.1403, 0.3613	0.1390, 0.3720	0.0471, 0.1229
R_1, wR_2 (all data)	0.1542, 0.3720	0.1495, 0.3853	0.0565, 0.1307
Largest diff. peak and hole ($e\cdot Å^{-3}$)	2.536 and –0.922	2.553 and –1.063	0.606 and –0.605
CCDC number	2539014	2559244	2539011

S2.8. Transformation by Humidity

Although the transformation from MIL-88A(Sc) to the different forms of MIL-53(Sc)-fum occurs quickly when immersed in water, the presence of water in the atmosphere alone is not enough to initiate the reaction. Samples of MIL-88A(Sc) were dried from MeOH and MeCN to the narrow pore phase, in accordance with our previous work,[S11] and left open on the bench for 4 weeks, exposed to air. No change in powder X-ray diffractograms were observed following exposure to air. Therefore, samples were placed in a humidity chamber consisting of a desiccator with water in the base. After two weeks, both samples clearly converted to **1**-100, as determined by PXRD (Figure S20).

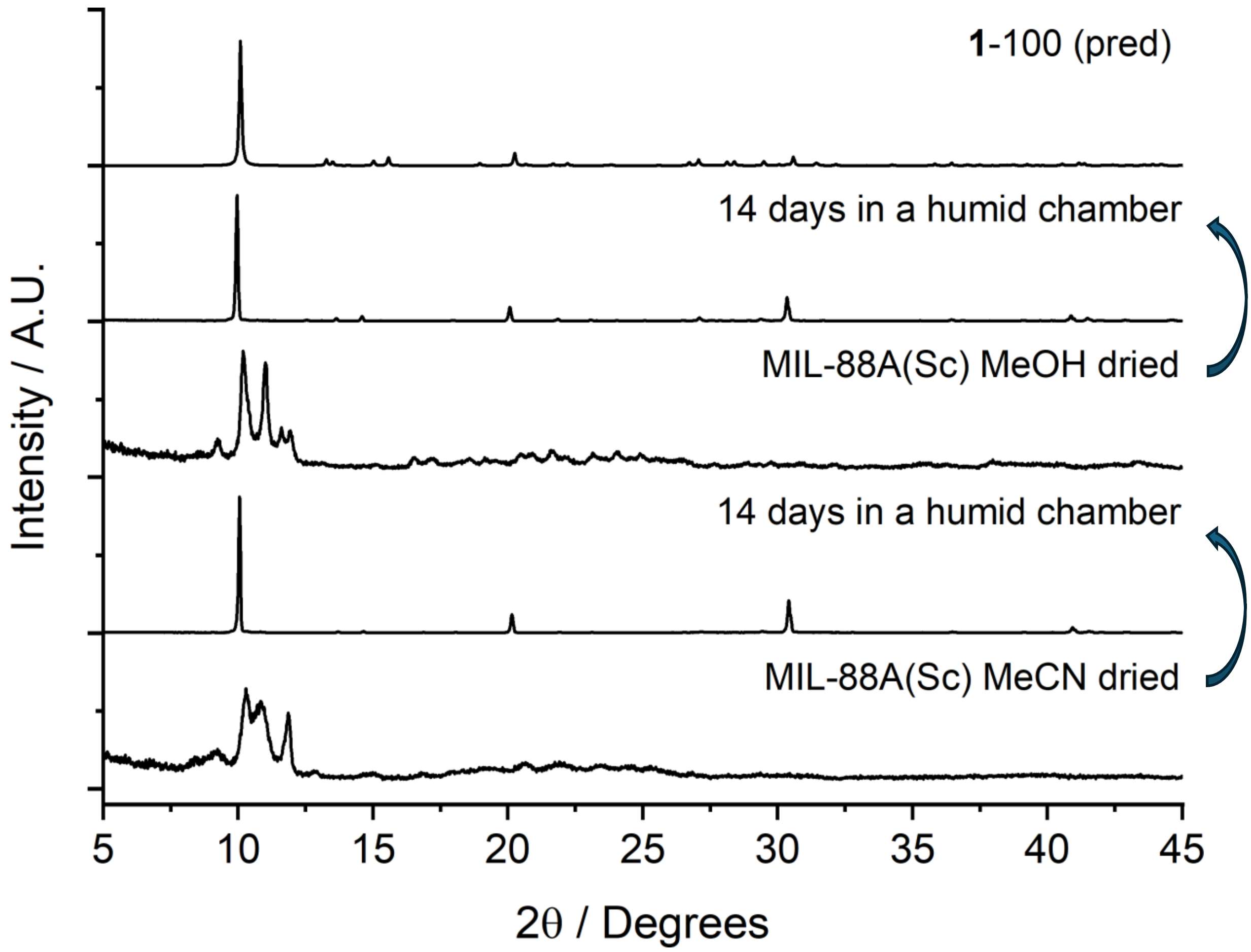

Figure S20. Stacked partial powder X-ray diffractograms of MIL-88A(Sc) dried from MeCN and MeOH into narrow pore forms, which were then placed in a humid chamber for 14 days, resulting in complete conversion to **1**-100, as judged by comparison with the pattern predicted form the crystal structure of **1**-100.

S2.9. Morphological Analysis

As-synthesised samples of **1**-100, **1**-75, **1**-50, and **1**-25 were imaged by scanning electron microscopy (SEM) and compared to SEM images of the precursor MIL-88A(Sc)[S11] (Figure S21). The images show a clear morphological change, from hexagonal prismatic needles (20-50 μm in length) that are characteristic of the MIL-88 topology, to irregular rectangular rods representative of the MIL-53 topology. The size of the rods seems to be dependent on the exchange solvent: **1**-100 presents as larger (up to 70 μm in length) rods than **1**-75 and **1**-50 (varying size from 1-30 μm in length). **1**-50 is narrower in diameter, which is consistent with our inability to obtain a single crystal structure of this sample. **1**-25 has even smaller particle size, predominantly composed of much thinner rods up to 10 μm in length. It is also possible to view some larger crystals of unreacted MIL-88A(Sc), which is consistent with the PXRD analysis in Figure S16.

Taken together, these observations highlight the critical role of solvent in the MOF-to-MOF transformation process, which is consistent with the solvent induced formation of different conformational isomers of MIL-53(Sc)-fum. It is also suggestive of a dissolution-recrystallisation mechanism, particularly with the micrograph of **1**-25 (Figure S21e) showing the presence of both MIL-88A(Sc) and MIL-53(Sc)-fum, and no intermediate materials (which is also consistent with the PXRD analyses of the transformations of all samples, when only both phases are present).

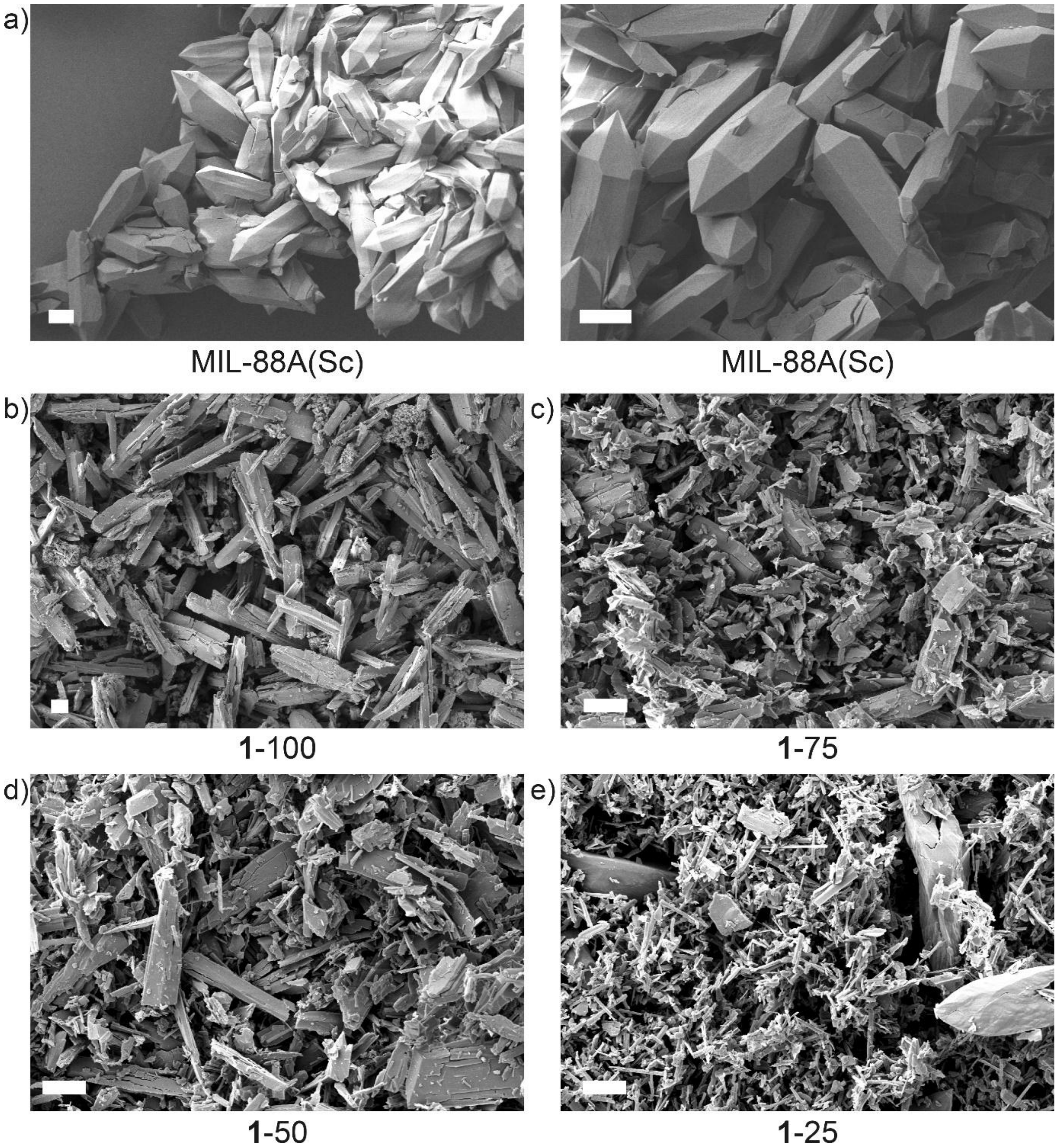

Figure S21. SEM images of as-synthesised a) MIL-88A(Sc),[S11] b) **1**-100, c) **1**-75, d) **1**-50, and e) **1**-25. Scale bars in all images are 10 µm.

S3. Activation and Flexibility

Samples of **1** were subjected to different activation conditions in order to assess structural flexibility. As homologues in the MIL-53(M)-fum have been found to be rigid, DFT was used to generate likely narrow pore structures of both the *(a)* and *(c)* confirmational isomers, as well as open pore models to complement the crystal structures of **1**-100 and **1**-75.

S3.1. DFT Modelling

We considered both type (*a*) and type (*c*) conformational isomers in our DFT simulations and, for each isomer, we considered both the open pore and narrow pore structures. The crystal structures of **1**-100 and **1**-75 were used as starting points to generate structural models of the open pore (*op*) forms of the (*a*) and (*c*) conformational isomers, respectively. The DFT simulations (*sim*) were named 1-*(a)-sim-op* and 1-*(c)-sim-op*, respectively, for the open pore forms of the two conformational isomers. To generate narrow pore (*np*) structures the DFT optimised open pore structure were used as the initial starting points, and further DFT geometry optimisations of the structures under a hydrostatic pressure of 1 GPa were run, which promoted the open-to-narrow pore phase transition. Subsequently, the hydrostatic pressure was removed and the narrow pore structures reoptimised using DFT under ambient pressure, where the narrow pore structures remained energetically stable.

Comparison of the predicted diffractograms from the DFT models of the two crystallographically observed isomers of **1**, (*a*) and (*c*), in the open pore (*op*) and narrow pore (*np*) forms, are given in Figure S22. The open pore models predict closely related diffractograms across the two conformational isomers. The narrow pore models predict similar diffractograms but with subtle differences in the position and intensity of certain Bragg peaks, suggesting the flexibility of the different conformational isomers may differ. This is in line with our previous work modelling MIL-53(Ga)-fum conformational isomers.[S12]

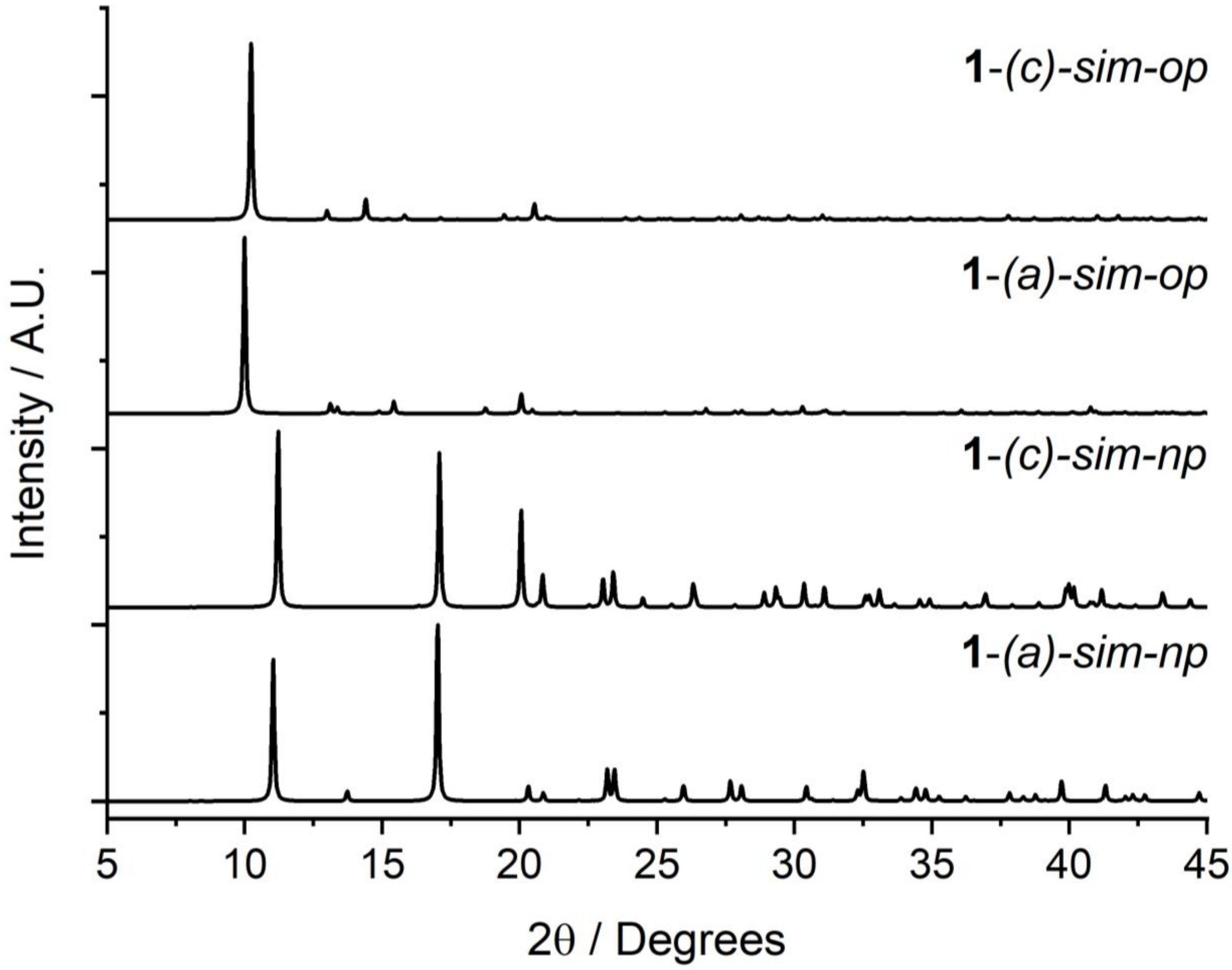

Figure S22. Stacked partial powder X-ray diffractograms predicted from the DFT models of the two conformational isomers identified in this study, in the open and narrow pore forms.

S3.2. Activation by Temperature and Vacuum

Variable temperature powder X-ray diffractograms (Figures S23-S25) also showed polymorph dependent behaviour. On heating to 250 °C, **1**-100*as* showed no further sign of closing, likely due to it already being in a more closed pore form than **1**-75*as* and **1**-50*as*.

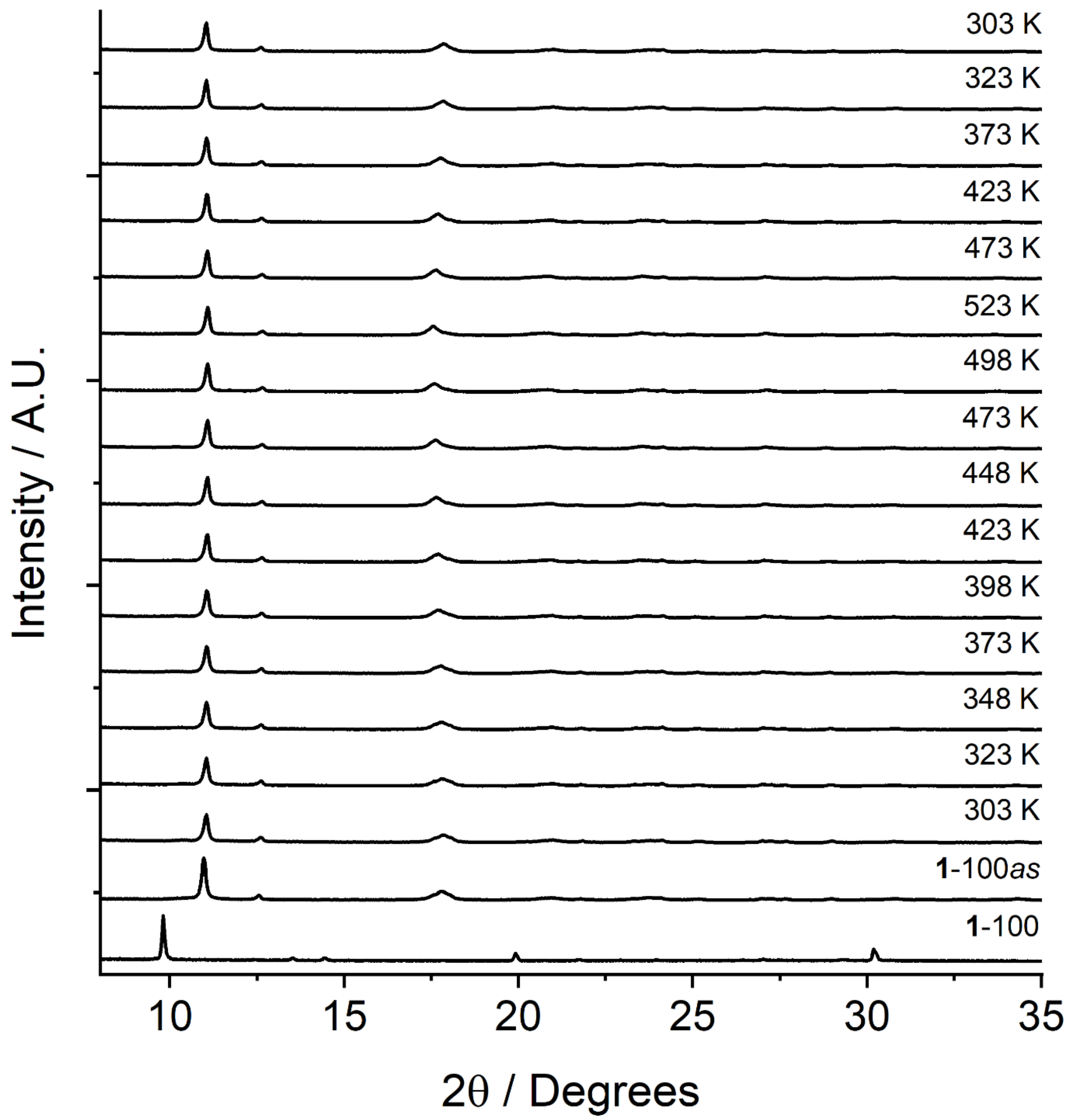

Figure S23. Stacked partial powder X-ray diffractograms (intensities normalised) of **1**-100*as* collected across a range of temperatures, compared to the diffractogram of **1**-100.

In contrast, both **1**-75*as* and **1**-50*as* showed a phase change that occurred between 75 and 125 °C, with the shifting of peaks to higher 2θ values suggesting significant structural flexibility and "closing" of the pores, likely due to loss of some of the DMF solvent. Some minor flexibility is also evident as peaks gradually move with varying temperatures.

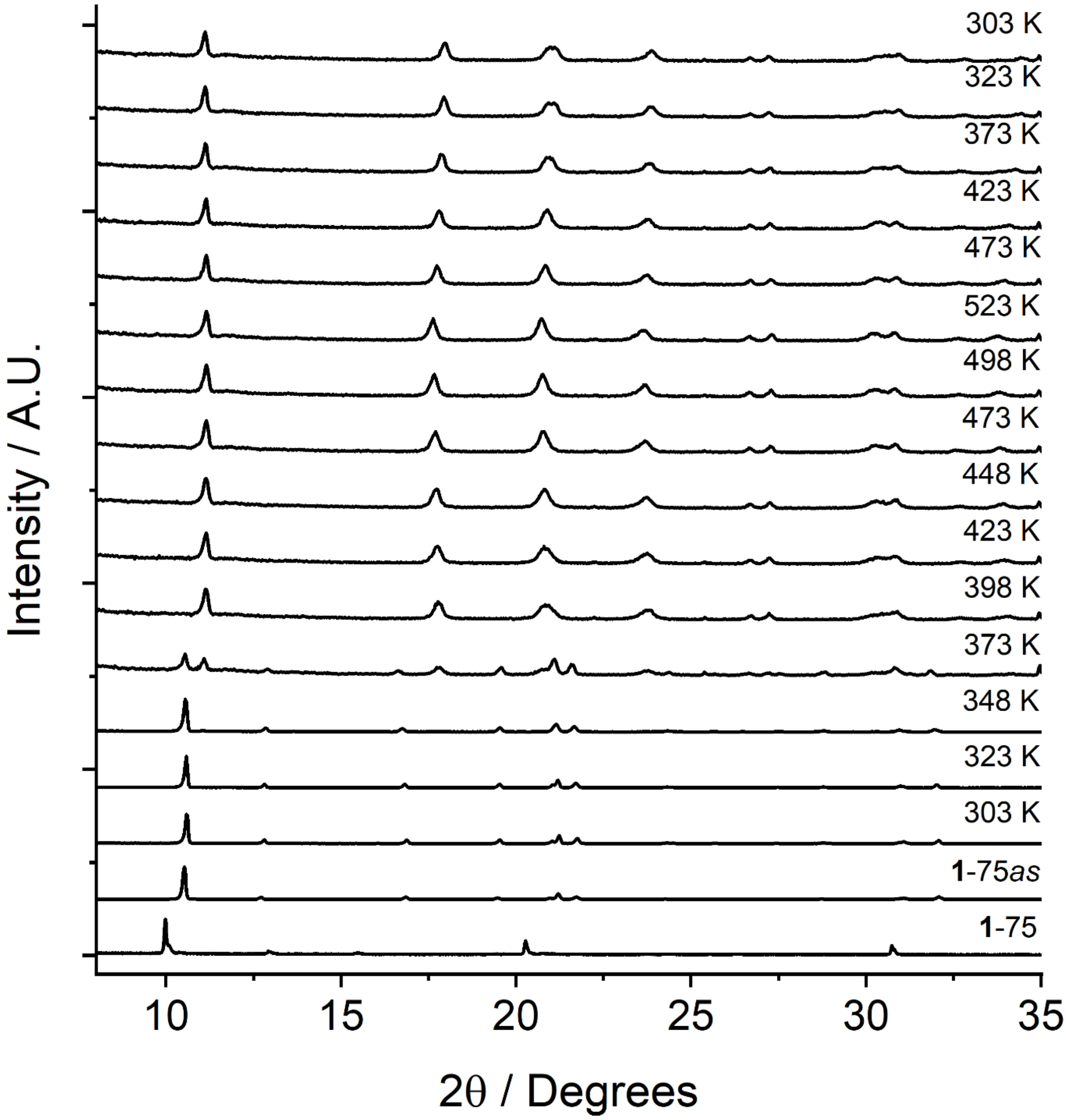

Figure S24. Stacked partial powder X-ray diffractograms (intensities normalised) of **1**-75*as* collected across a range of temperatures, compared to the diffractogram of **1**-75. The peaks emerging at 2θ ~ 25.4 and 35 ° come from the corundum holder.

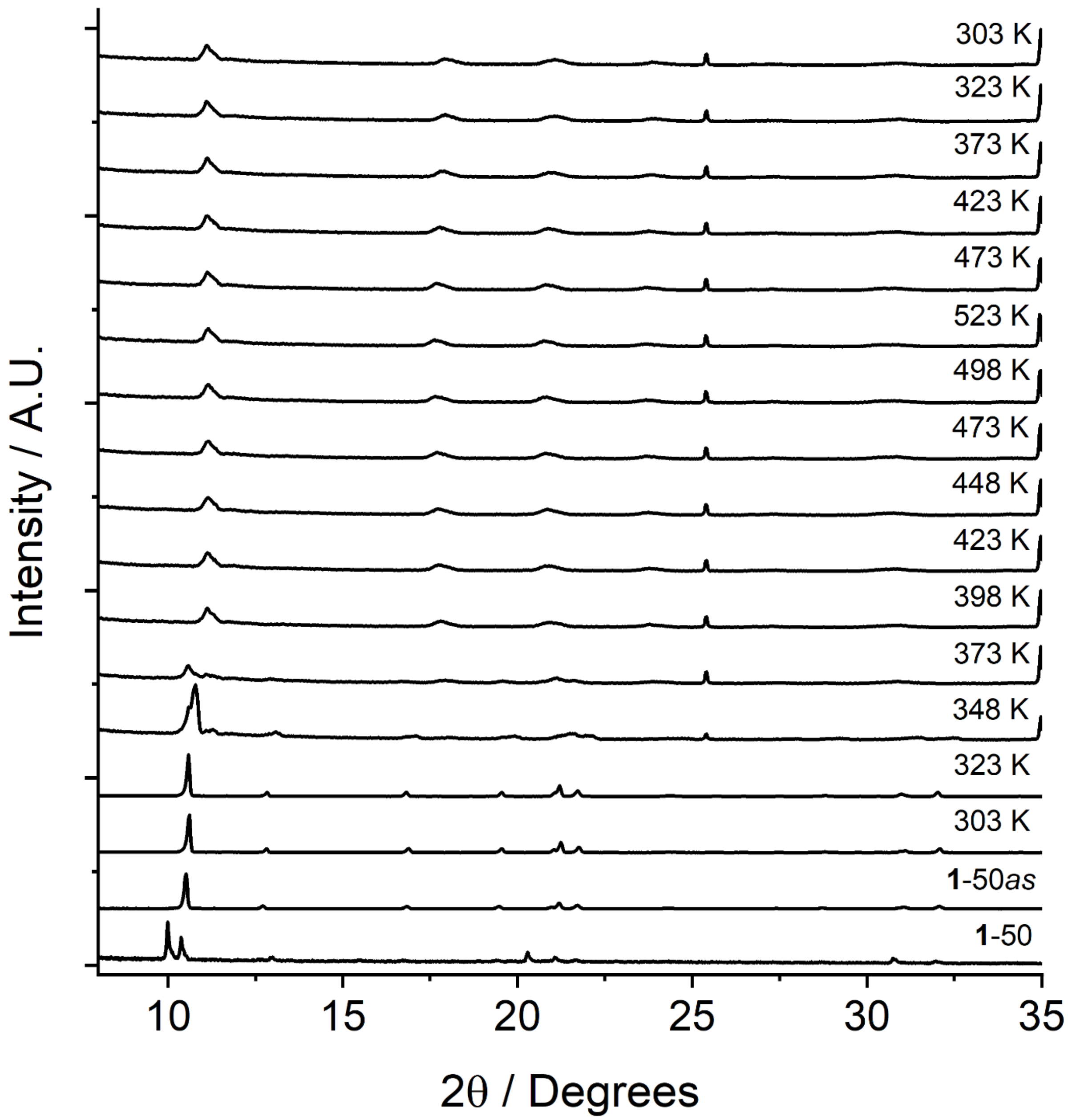

Figure S25. Stacked partial powder X-ray diffractograms (intensities normalised) of **1**-50*as* collected across a range of temperatures, compared to the diffractogram of **1**-50. The peaks emerging at 2θ ~ 25.4 and 35 ° come from the corundum holder.

By comparing the diffractograms of **1**-100*as*, **1**-75*as*, and **1**-50*as* collected at 498 K (Figure S26), it is clear that all three samples have closed to approximately the same narrow pore form. The difference in behaviour in the variable temperature patterns can

be explained by **1**-100*as* spontaneously closing when dried at room temperature, whereas **1**-75*as* and **1**-50*as* require additional heating.

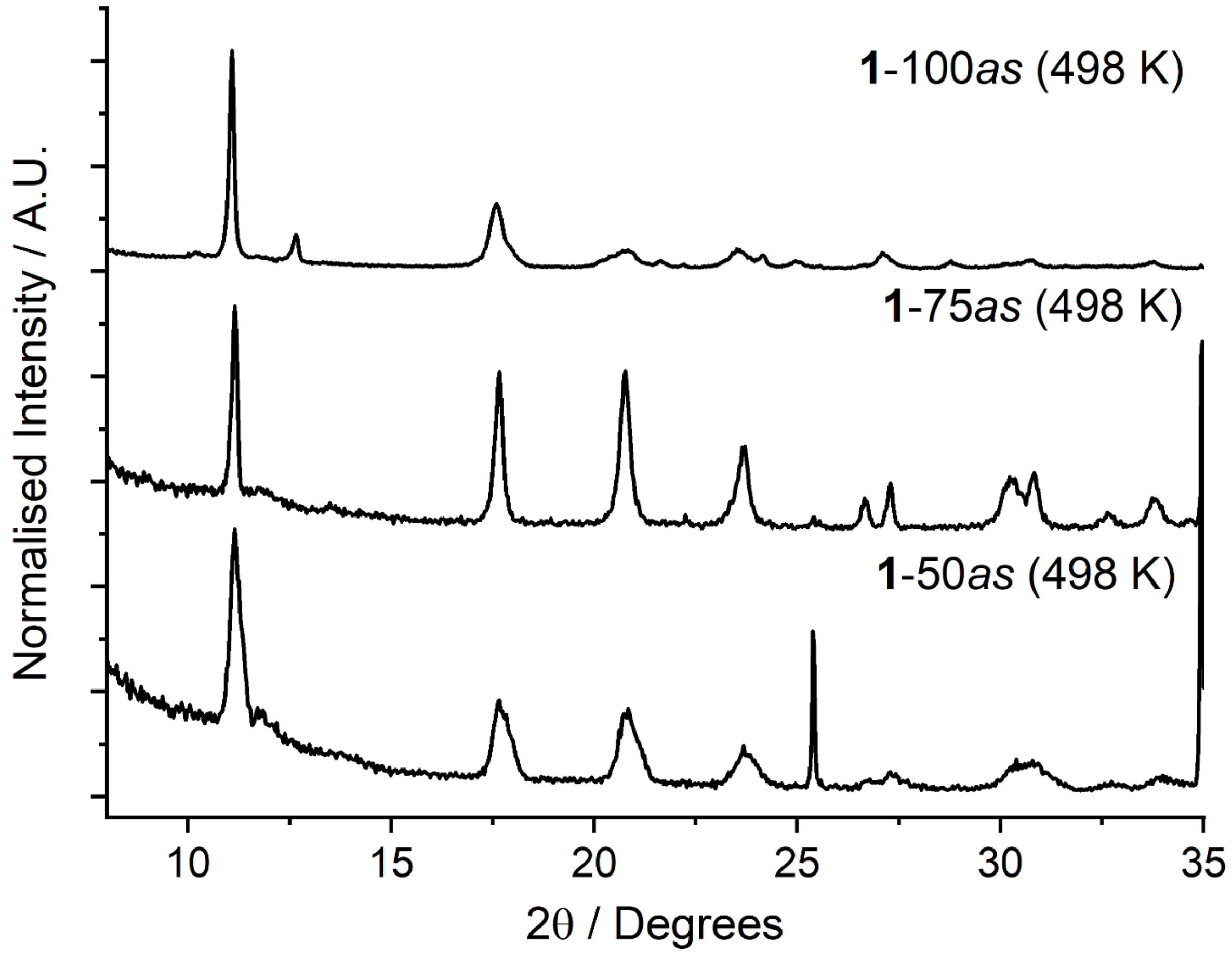

Figure S26. Stacked partial powder X-ray diffractograms (intensities normalised) of **1**-100*as*, **1**-75*as*, and **1**-50*as* collected at 498 K. The peaks emerging at 2θ ~ 25.4 and 35 ° come from the corundum holder.

Complete removal of pore solvent was confirmed by ^{1}H NMR spectroscopy of samples that had been acid digested (Figure S27).

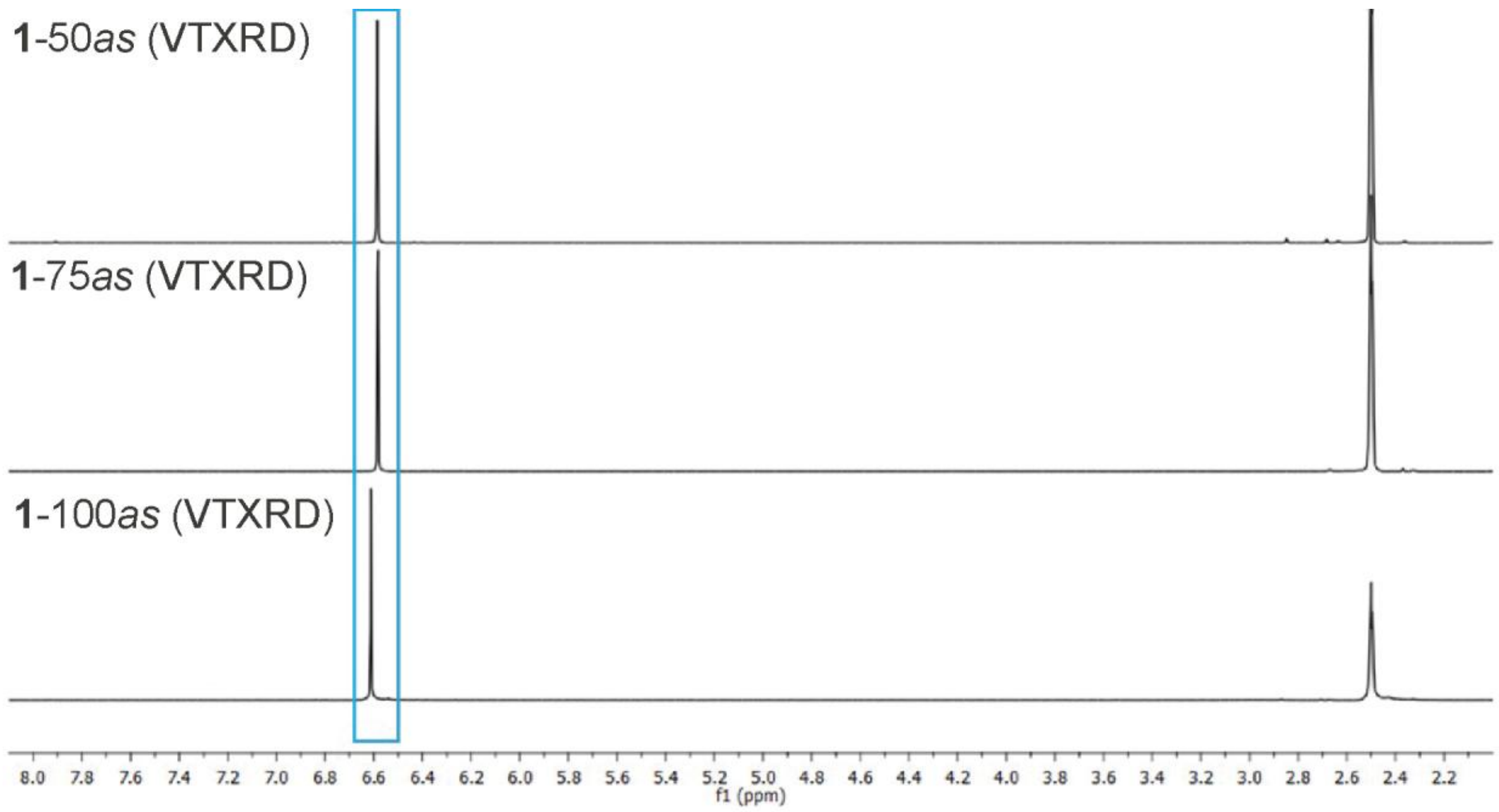

Figure S27. Stacked partial ^{1}H NMR spectra of acid-digested (DMSO-d_6/D_2SO_4) samples of **1**-100*as*, **1**-75*as*, and **1**-50*as* after they had been analysed by variable temperature PXRD (heated to 523 K under ambient conditions). The resonances assigned to the fumarate protons are in boxed in blue. The resonances around 2.5 ppm correspond to residual DMSO.

As the solvent can be removed by heating, the samples were activated for gas adsorption analysis by heating to 423 K under 10^{-4} Torr vacuum for 20 h and analysed by PXRD (Figure S28).

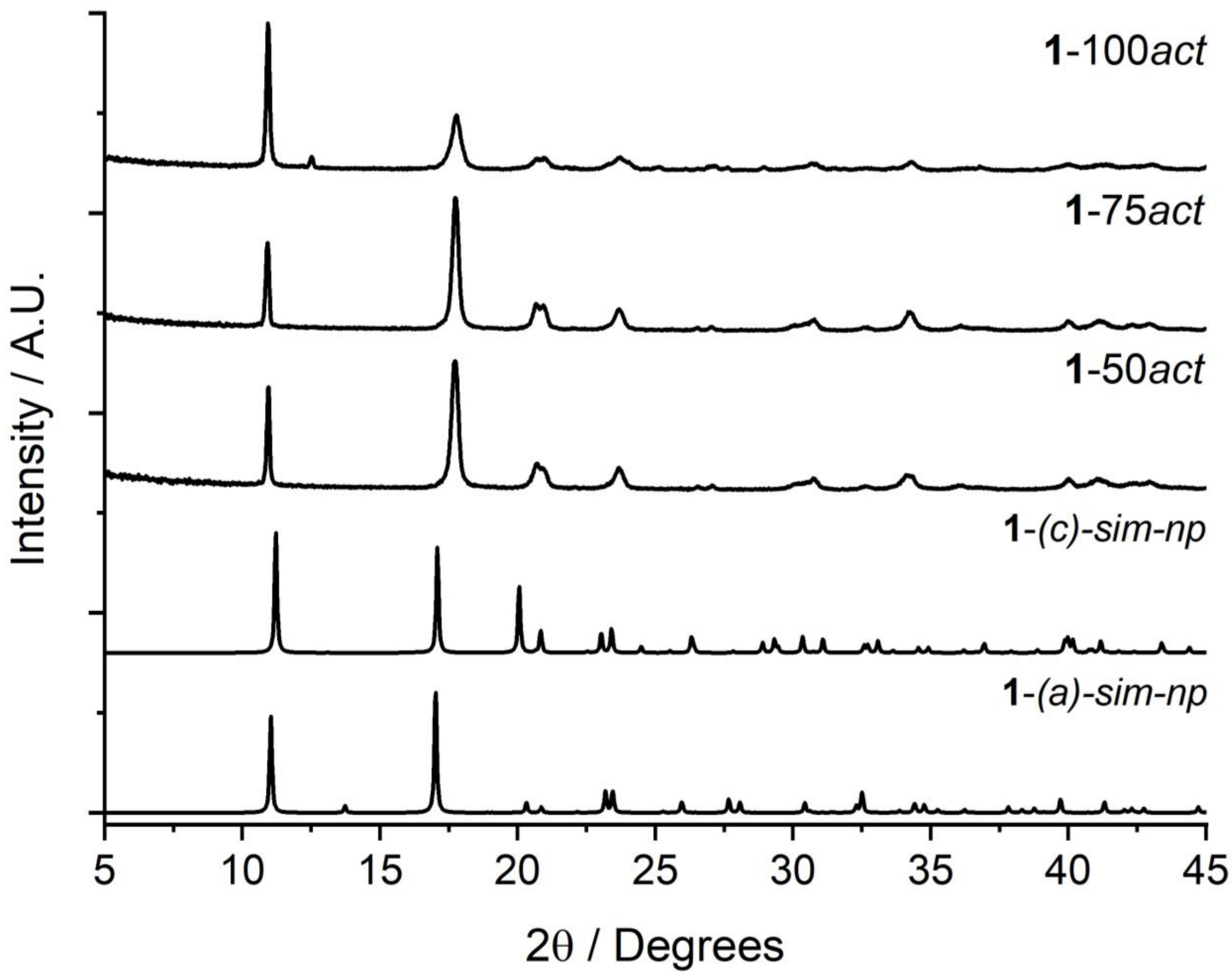

Figure S28. Stacked partial powder X-ray diffractograms of activated **1**-100*act*, **1**-75*act*, and **1**-50*act* in comparison to those predicted from the DFT models of the narrow pore forms of the *(a)* and *(c)* polymorphs, **1**-*(a)-sim-np* and **1**-*(c)-sim-np*.

It was noted that the diffractograms for **1**-100*as* and **1**-100*act* are very similar, suggesting that the final activation step did not change the overall structure. **1**-100*act* has a similar but subtly different diffractogram compared to **1**-75*act* and **1**-50*act*; peak positions are similar but intensities and broadness vary. Portions of these diffractograms are also presented in the main manuscript, in Figure 4a.

As a representative sample, the diffractogram of **1**-50*act* was subjected to Pawley fitting (Figure 4c of the main manuscript) which confirmed formation of a closed pore material with a significantly decreased unit cell volume.

N_2 adsorption/desorption isotherms (77 K) up to 1 bar (Figure S29) showed no microporosity, also indicating the samples adopted a closed pore form on activation.

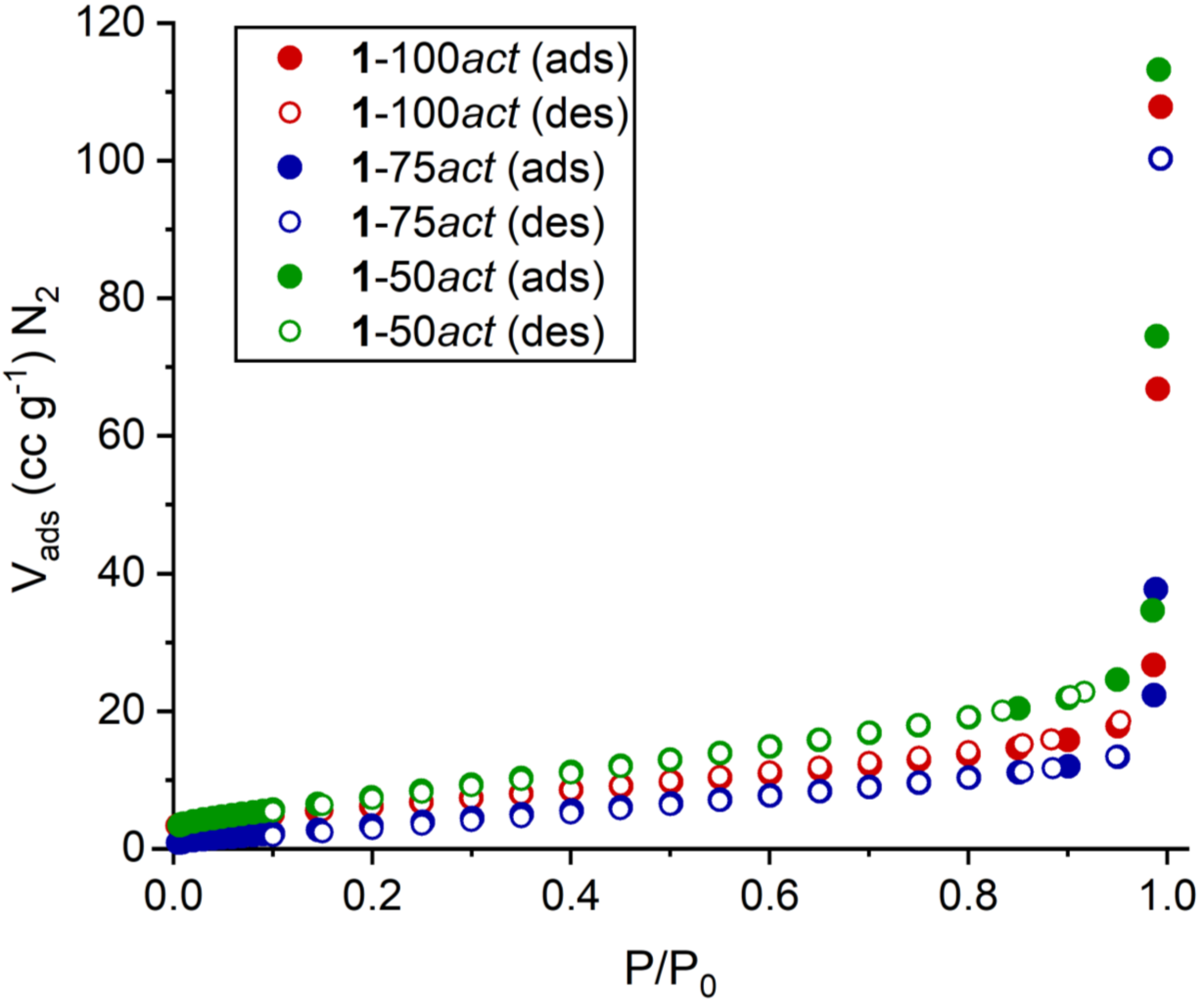

Figure S29. N_2 adsorption/desorption isotherms (77 K) of activated samples, **1**-100*act*, **1**-75*act*, and **1**-50*act*, showing the samples to be essentially non-porous to N_2 under these conditions.

The same samples of **1**-100*act*, **1**-75*act* and **1**-50*act* were reanalysed by PXRD after 49 months in storage under an ambient atmosphere. The diffractograms (Figure S30) are closely related to those collected at the time of synthesis and activation (Figure S28), with only minor differences in relative peak intensities that can be attributed to differing extents of preferred orientation across the different experiments. This indicates the long-term stability of MIL-53(Sc)-fum under storage.

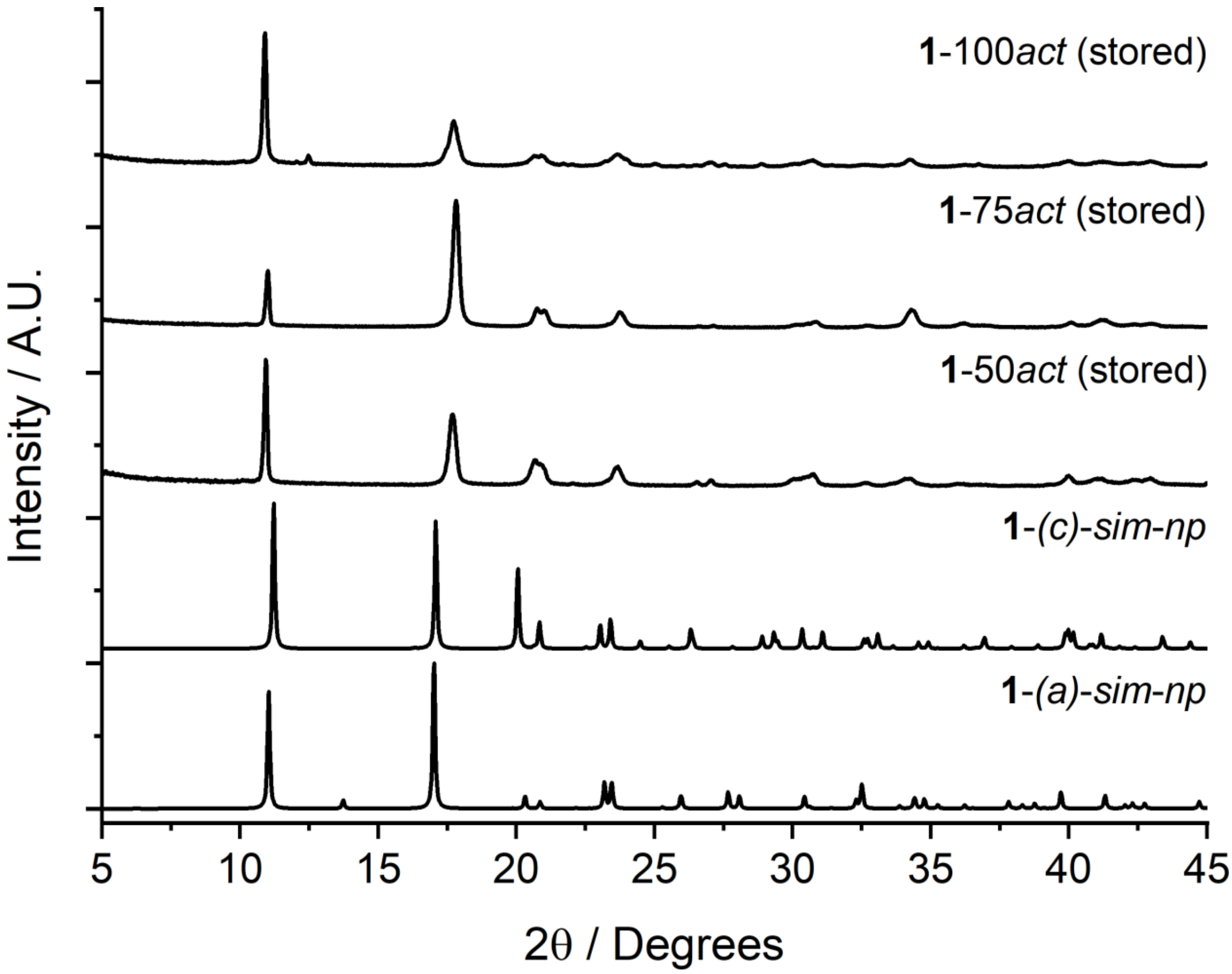

Figure S30. Stacked partial powder X-ray diffractograms of activated **1**-100*act*, **1**-75*act*, and **1**-50*act* collected after 49 months in storage under an ambient atmosphere, in comparison to those predicted from the DFT models of the narrow pore forms of the *(a)* and *(c)* polymorphs, **1**-*(a)-sim-np* and **1**-*(c)-sim-np*. The samples are the same as those measured in Figure S28.

3.3. Acetone Solvent Exchange and Activation

As-synthesised samples were attempted to be solvent exchanged with acetone by the following general procedure. Around 50 mg of **1**-100*as*, **1**-75*as*, or **1**-50*as* were placed in a round-bottom flask and acetone was added. The mixture was refluxed overnight, without stirring, cooled to room temperature and transferred to a centrifuge tube, where it was centrifuged, and the mother solvent was decanted. The solids were collected and dried at room temperature in a vacuum desiccator prior to analysis (as-synthesised). The materials were activated at 150 °C for 20 hours under 10^{-4} Torr vacuum (activated).

Powder X-ray diffraction analyses of **1**-100*ace-as*, **1**-75*ace-as* and **1**-50*ace-as* showed the samples remained in an open pore form (Figure S31), whilst NMR spectroscopy of acid digests (Figure S32) and TGA (Figure S33) confirm the presence of significant residual solvents.

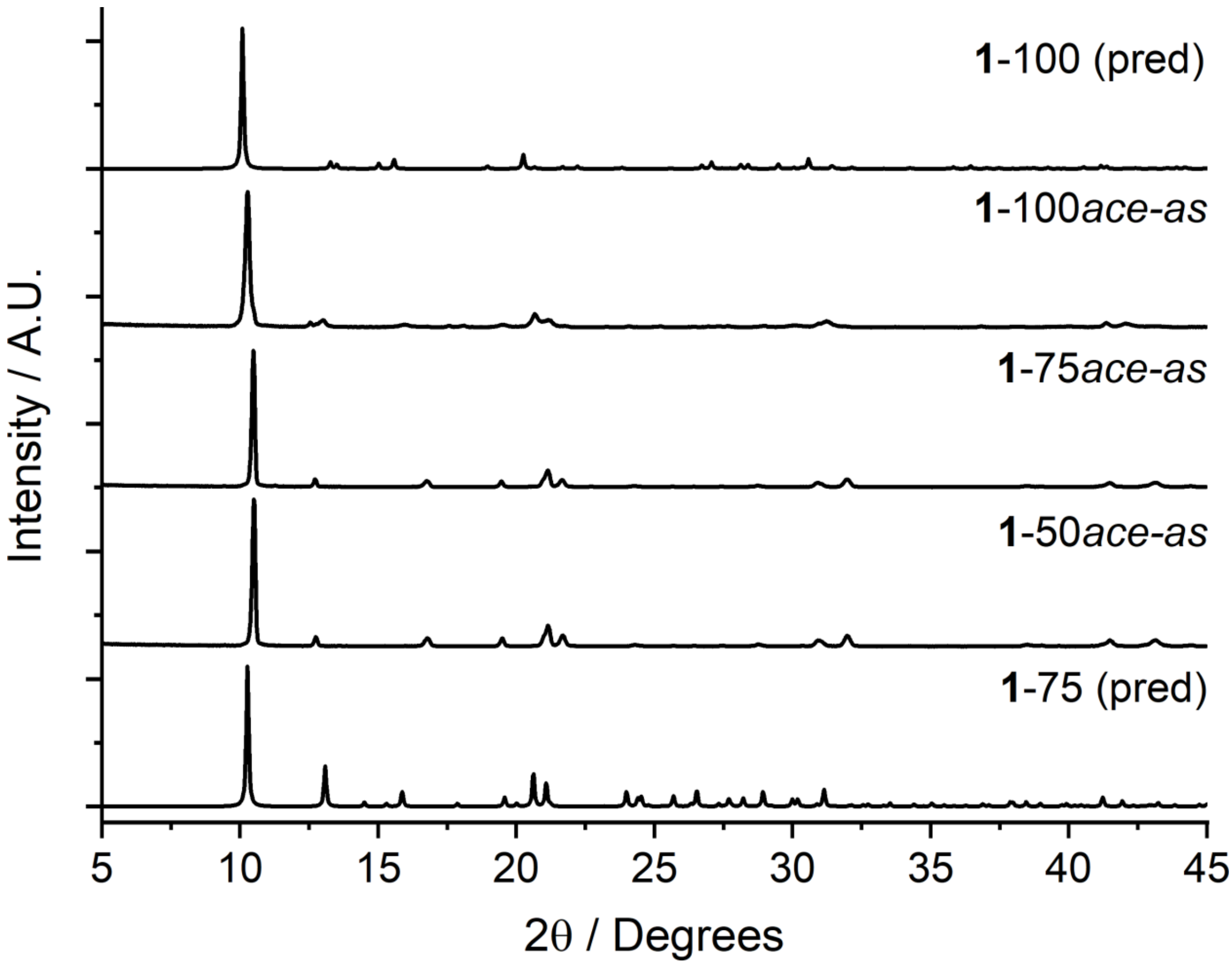

Figure S31. Stacked partial powder X-ray diffractograms of acetone exchanged samples, **1**-100*ace-as*, **1**-75*ace-as*, and **1**-50*ace-as* in comparison to those predicted from the crystal structures of **1**-100 and **1**-75.

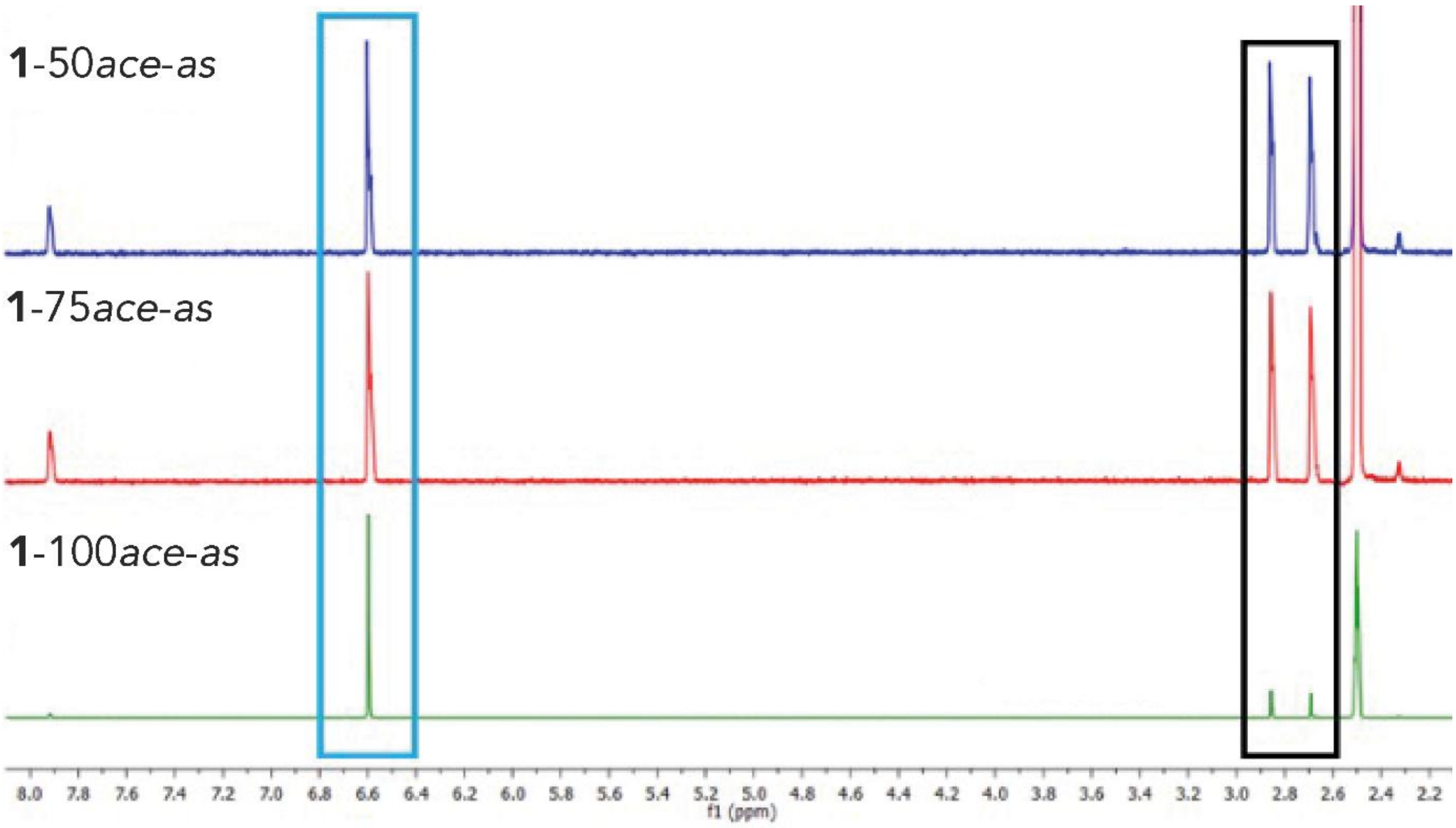

Figure S32. Stacked partial ^{1}H NMR spectra of acid-digested (DMSO-d_6/D_2SO_4) samples of acetone exchanged, **1**-100*ace-as*, **1**-75*ace-as*, and **1**-50*ace-as*. Resonances assigned to fumarate protons boxed in blue, resonances assigned to methyl protons of DMF protons boxed in black.

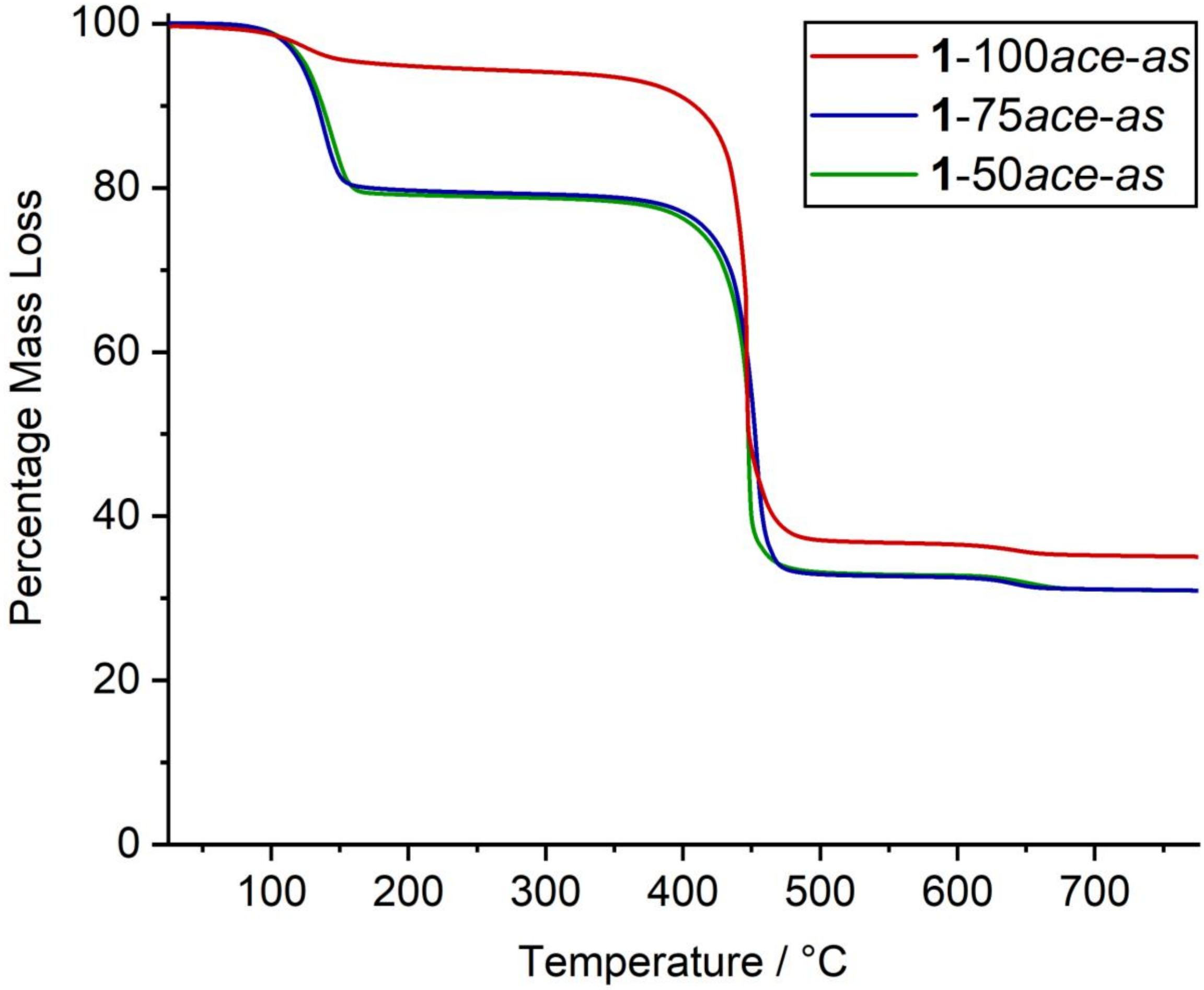

Figure S33. Thermogravimetric analysis traces of acetone exchanged samples, **1**-100*ace-as*, **1**-75*ace-as*, and **1**-50*ace-as*. The lower solvent retention of **1**-100*ace-as* is also evident in the acid digestion ^{1}H NMR spectrum (Figure S32).

Based on the NMR spectra of acid digested samples and the residues remaining from TGA analyses, the proposed compositions of the samples are summarised in Table S4.

Table S4. Proposed formulae for the acetone exchanged, as synthesised samples, based on ^{1}H NMR spectroscopy and TGA.

Sample	Proposed Formula	Predicted Residue	Actual Residue
**1**-100*ace-as*	$[Sc(OH)(C_4H_2O_4)]\cdot(C_3H_7NO)_{0.28}$	35.1% wt	35.0% wt
**1**-75*ace-as*	$[Sc(OH)(C_4H_2O_4)]\cdot(C_3H_7NO)_{0.67}$	30.6% wt	30.9% wt
**1**-50*ace-as*	$[Sc(OH)(C_4H_2O_4)]\cdot(C_3H_7NO)_{0.70}$	30.4% wt	30.9% wt

As such, the samples were activated under standard conditions. PXRD (Figure S34 showed all adopt a closed pore form, which was assess by Pawley fitting the diffractogram of **1**-50*ace-act* (Figure S35) as a representative of the acetone exchanged, activated samples. The fit is very close to that of the directly activated analogue, **1**-50*act* (Figure 4c, main manuscript), confirming the closed pore formation. Acid digestion NMR spectroscopy (Figure S36) and TGA (Figure S37) indicate removal of pore solvents, summarised in Table S5, but N_2 adsorption isotherms (Figure S38) showed no notable porosity as with the directly activated samples. As such, acetone activation was not pursued further.

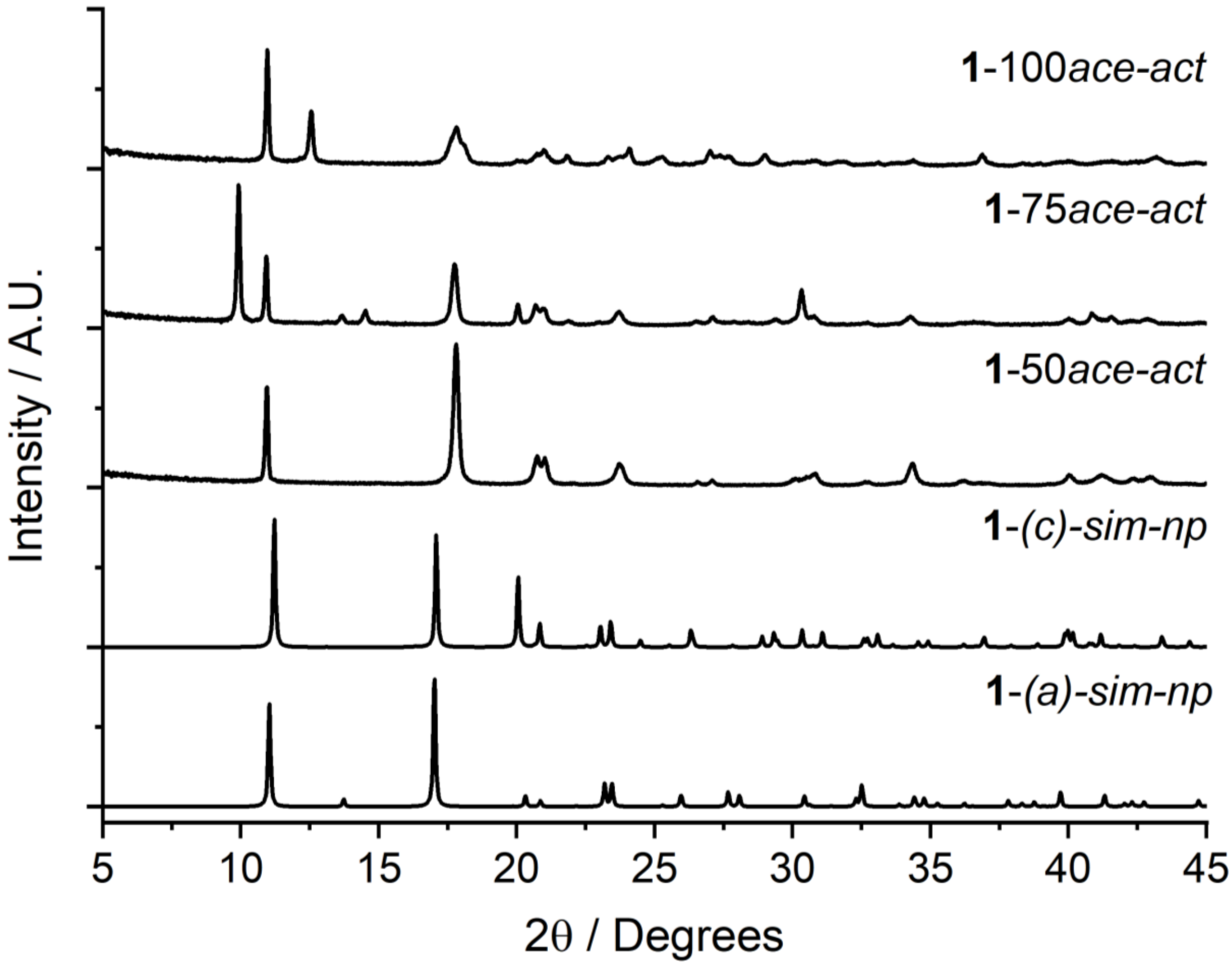

Figure S34. Stacked partial powder X-ray diffractograms of acetone exchanged and activated samples, **1**-100*ace-act*, **1**-75*ace-act*, and **1**-50*ace-act* in comparison to those predicted from the DFT models of the narrow pore forms of the *(a)* and *(c)* polymorphs, **1**-*(a)-sim-np* and **1**-*(c)-sim-np*.

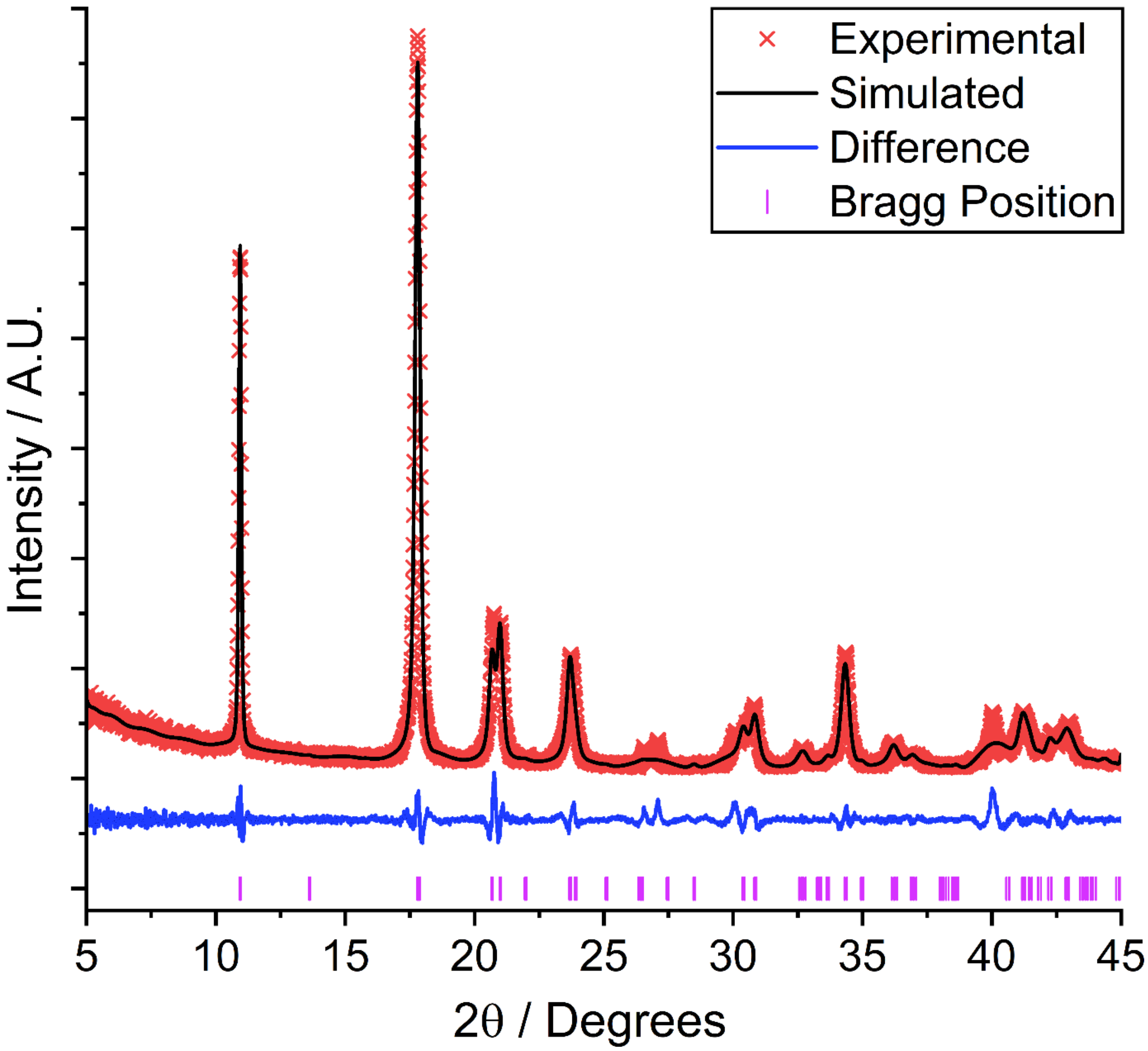

Figure S35. Pawley fitting of the powder X-ray diffractogram of **1**-50*ace-act* as a representative fit for all activated samples. Parameters: a = 7.563 Å, b = 16.484 Å, c = 5.576 Å; β = 107.05 °; V = 665 Å^3; R_p = 7.98%, wR_p = 11.04%.

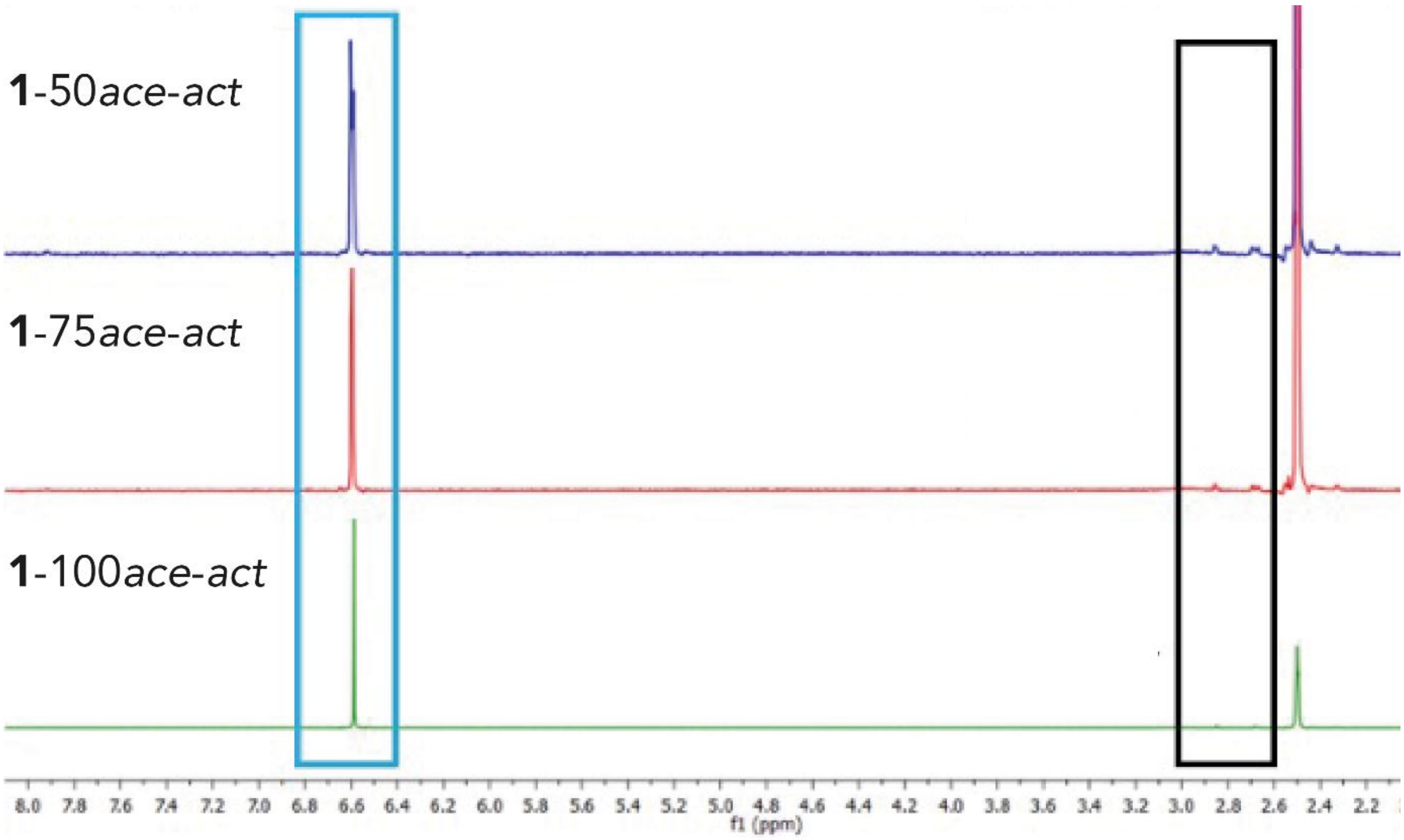

Figure S36. Stacked partial ^{1}H NMR spectra of acid-digested (DMSO-d_6/D_2SO_4) samples of acetone exchanged and activated samples, **1**-100*ace-act*, **1**-75*ace-act*, and **1**-50*ace-act*. Resonances assigned to fumarate protons boxed in blue, resonances assigned to methyl protons of DMF protons boxed in black.

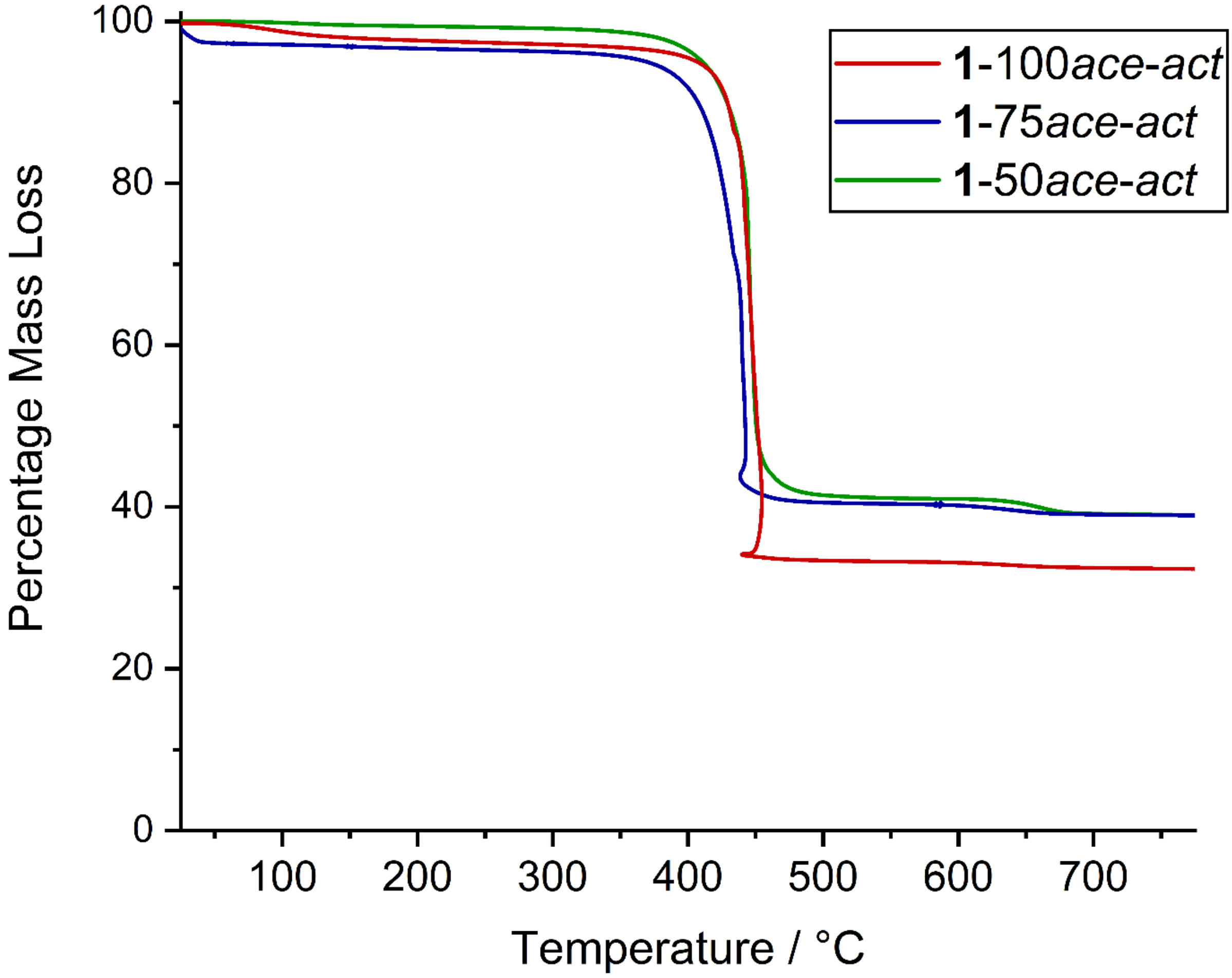

Figure S37. Thermogravimetric analysis traces of acetone exchanged and activated samples, **1**-100*ace-act*, **1**-75*ace-act*, and **1**-50*ace-act*.

Table S5. Proposed formulae for the acetone exchanged, activated samples, based on ^{1}H NMR spectroscopy and TGA.

**Sample**	**Proposed Formula**	**Predicted Residue**	**Actual Residue**
**1**-100*ace-act*	$[Sc(OH)(C_4H_2O_4)]\cdot(C_3H_7NO)_{0.01}$	39.0% wt	32.3% wt
**1**-75*ace-act*	$[Sc(OH)(C_4H_2O_4)]\cdot(C_3H_7NO)_{0.02}$	38.9% wt	38.9% wt
**1**-50*ace-act*	$[Sc(OH)(C_4H_2O_4)]\cdot(C_3H_7NO)_{0.03}$	38.7% wt	39.0% wt

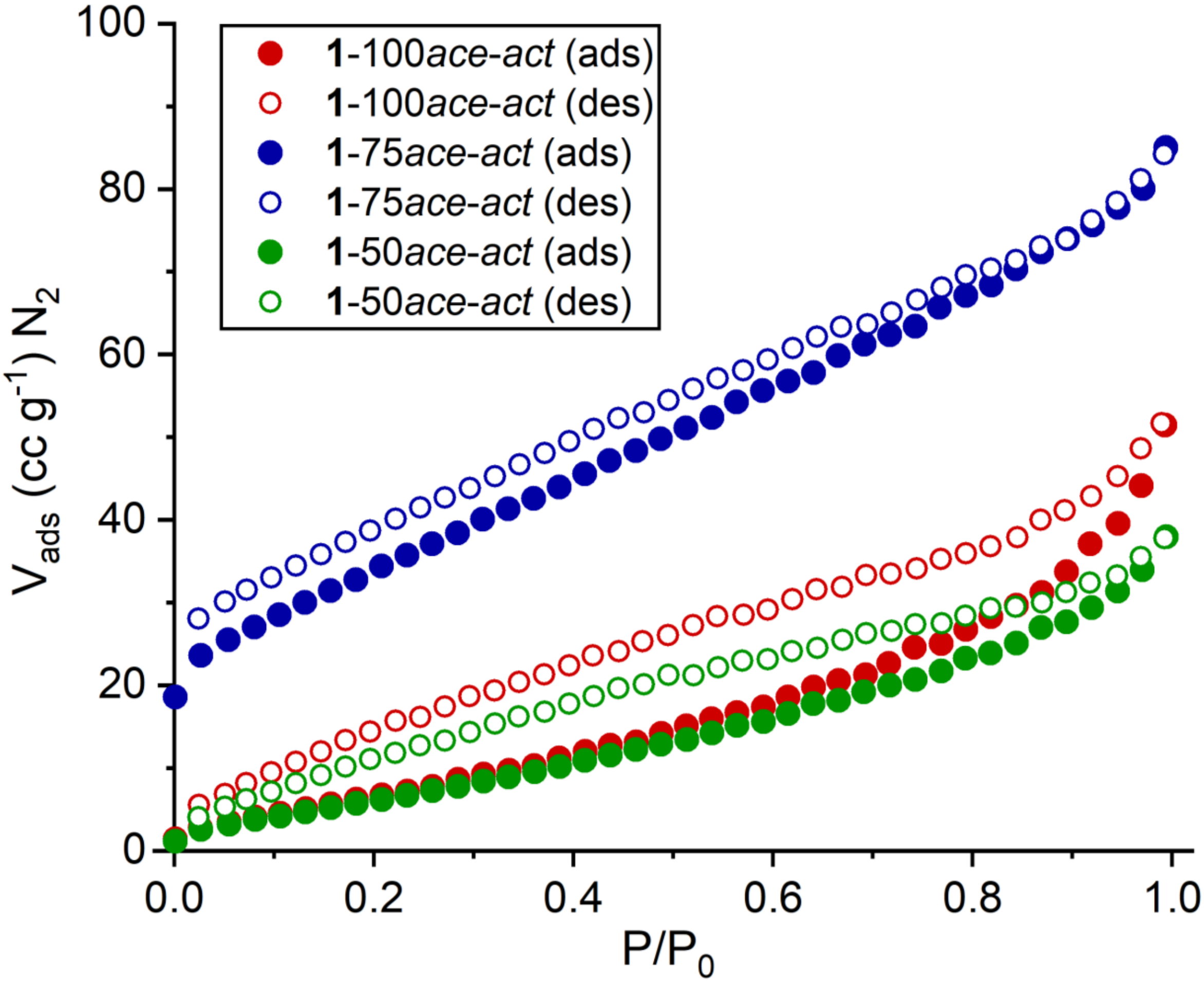

Figure S38. N_2 adsorption/desorption isotherms (77 K) of acetone-exchanged, activated samples, **1**-100*ace-act*, **1**-75*ace-act*, and **1**-50*ace-act*.

3.4. Methanol Solvent Exchange and Activation

3.4.1. DFT Modelling

DFT optimised structures of the two conformational isomers of **1** with varying degrees of methoxylation – 50:50 mixing of OH and OCH_3, and 100% OCH_3 – were generated by exchanging either half of the OH moieties (projecting along one side of the SBU) or all of the OH units with OCH_3 groups in the crystal structures of **1**-100 and **1**-75. These initial structures were optimised by DFT as per the general method to generate simulated open pore forms of the (*a*) and (*c*) conformational isomers: **1**-*(a)*-*sim*-$(OH)_{0.5}(OCH_3)_{0.5}$-*op* and

1-*(c)-sim*-(OH)$_{0.5}$(OCH$_3$)$_{0.5}$-*op*, 50% methoxylation; **1**-*(a)-sim*-(OCH$_3$)-*op* and **1**-*(c)-sim*-(OCH$_3$)-*op*, 100% methoxylation (Figure S39).

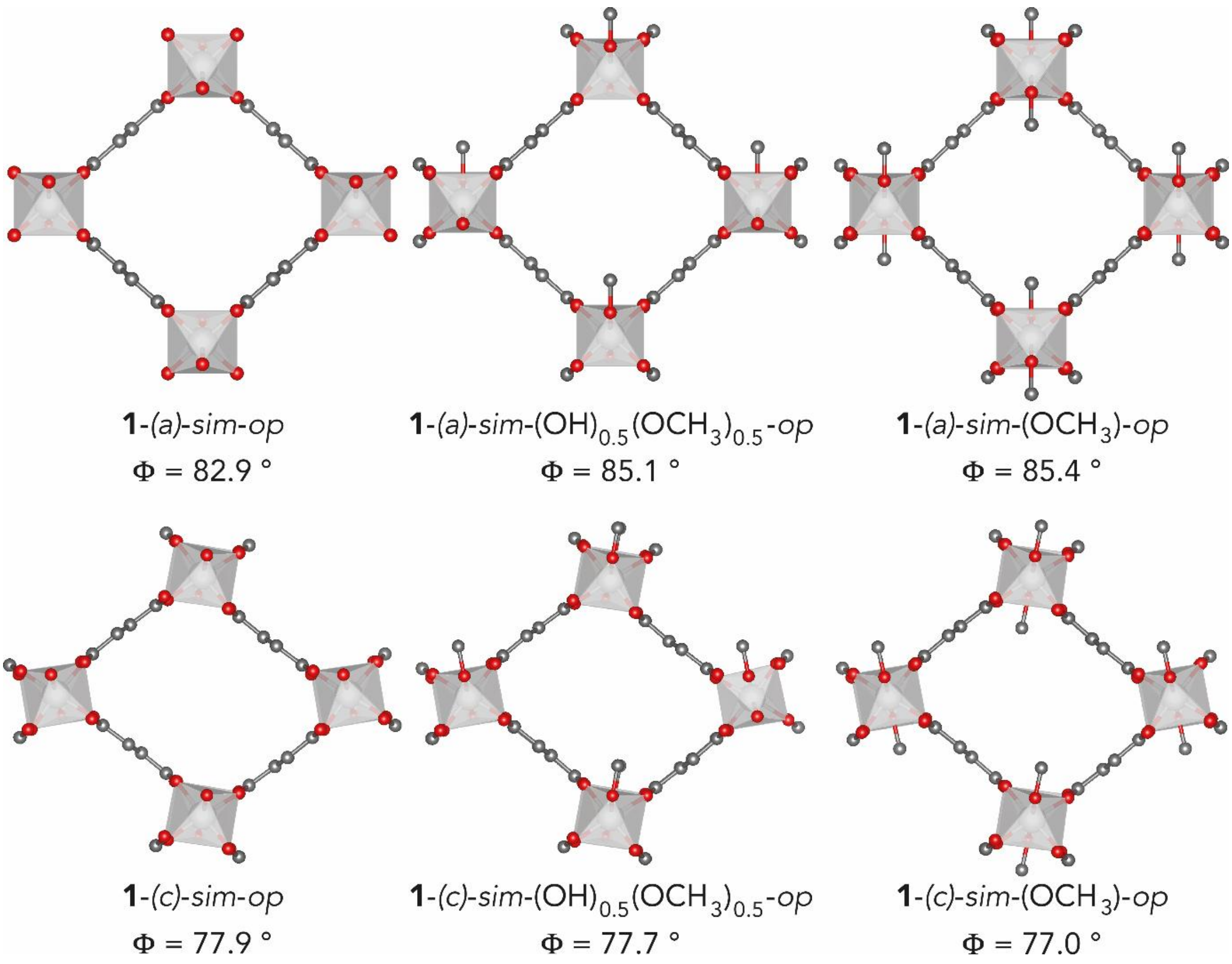

Figure S39. Comparison of the DFT simulated open pore structures of the *(a)* and *(c)* conformational isomers with differing amounts (0%, 50%, and 100%) of μ$_2$-OCH$_3$ substitution. The extent of "open-ness" is quantified by the pore angle Φ (values are an average due to the lowered symmetry of the simulated structures). Increasing cluster anion substitution of μ$_2$-OH by μ$_2$-OCH$_3$ has negligible effect on the Φ values.

Comparison of the predicted diffractograms (Figure S40) for all simulated open pore forms across the (*a*) and (*c*) conformational isomers showed that methoxylation does not affect the extent of opening, which would be expected as the open pore form can comfortably accommodate the additional steric bulk of the μ$_2$-OCH$_3$ unit compared to the μ$_2$-OH group of the parent structure.

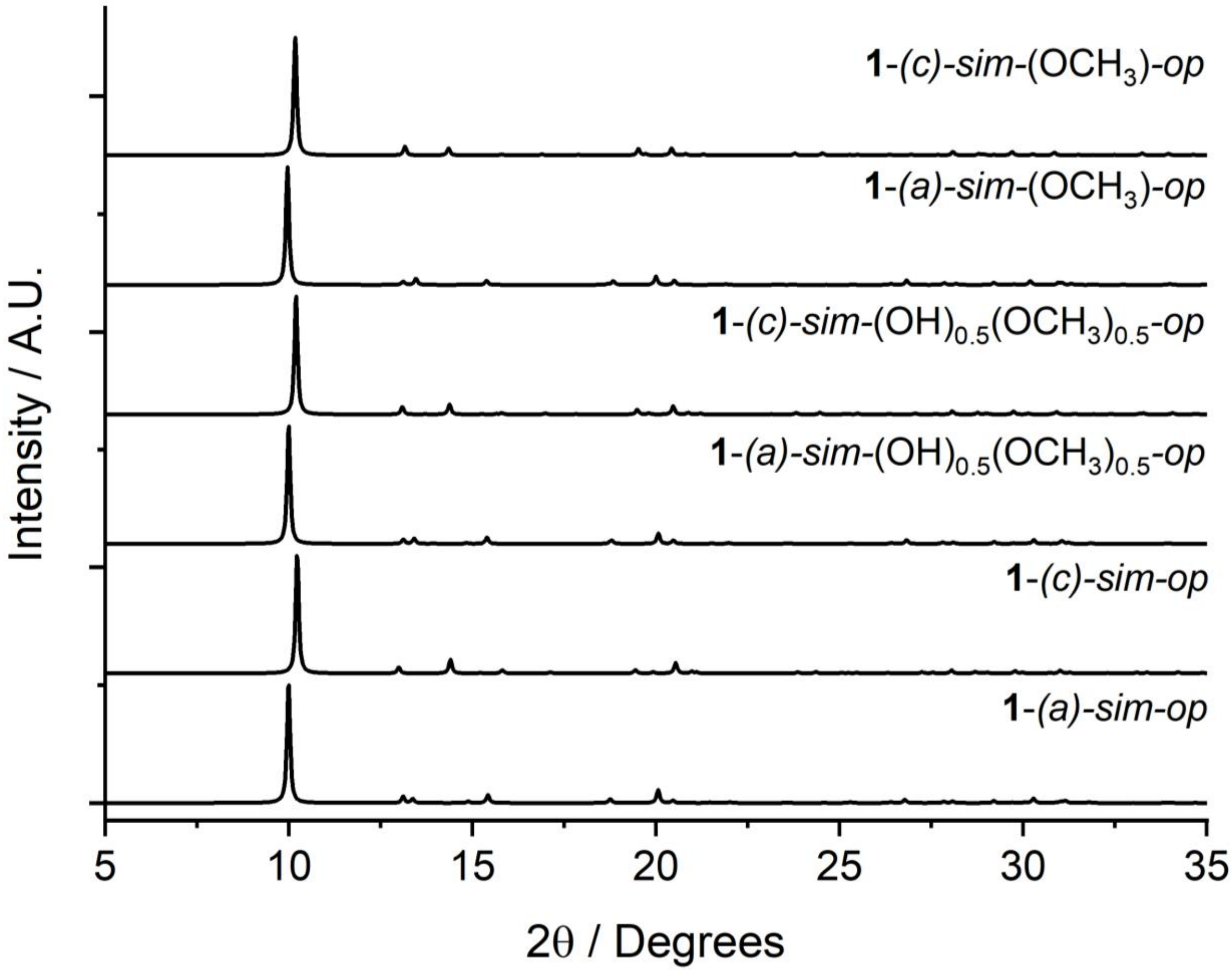

Figure S40. Stacked partial powder X-ray diffractograms predicted from the DFT models of the open arrow pore forms of the (*a*) and (*c*) polymorphs with different levels of cluster methoxylation: **1**-*(a)-sim-op* and **1**-*(c)-sim-op*, 0% methoxylation; **1**-*(a)-sim*-$(OH)_{0.5}(OCH_3)_{0.5}$-*op* and **1**-*(c)-sim*-$(OH)_{0.5}(OCH_3)_{0.5}$-*op*, 50% methoxylation; **1**-*(a)-sim*-(OCH_3)-*op* and **1**-*(c)-sim*-(OCH_3)-*op*, 100% methoxylation.

DFT simulated models of the methoxylated closed pore forms were generated by same methodology as for the unfunctionalized structure (Section S3.1), generating the models: **1**-*(a)-sim*-$(OH)_{0.5}(OCH_3)_{0.5}$-*np* and **1**-*(c)-sim*-$(OH)_{0.5}(OCH_3)_{0.5}$-*np*, 50% methoxylation; **1**-*(a)-sim*-(OCH_3)-*np* and **1**-*(c)-sim*-(OCH_3)-*np*, 100% methoxylation. These DFT simulated structures of the closed pore materials predict that increasing the extent of cluster anion substitution, from 0% methoxylation to 50% to 100%, dramatically alters the flexibility of both constitutional isomers; the greater the methoxylation, the more "open" the structure will be (as assessed by measuring pore angle ϕ, Figure S41).

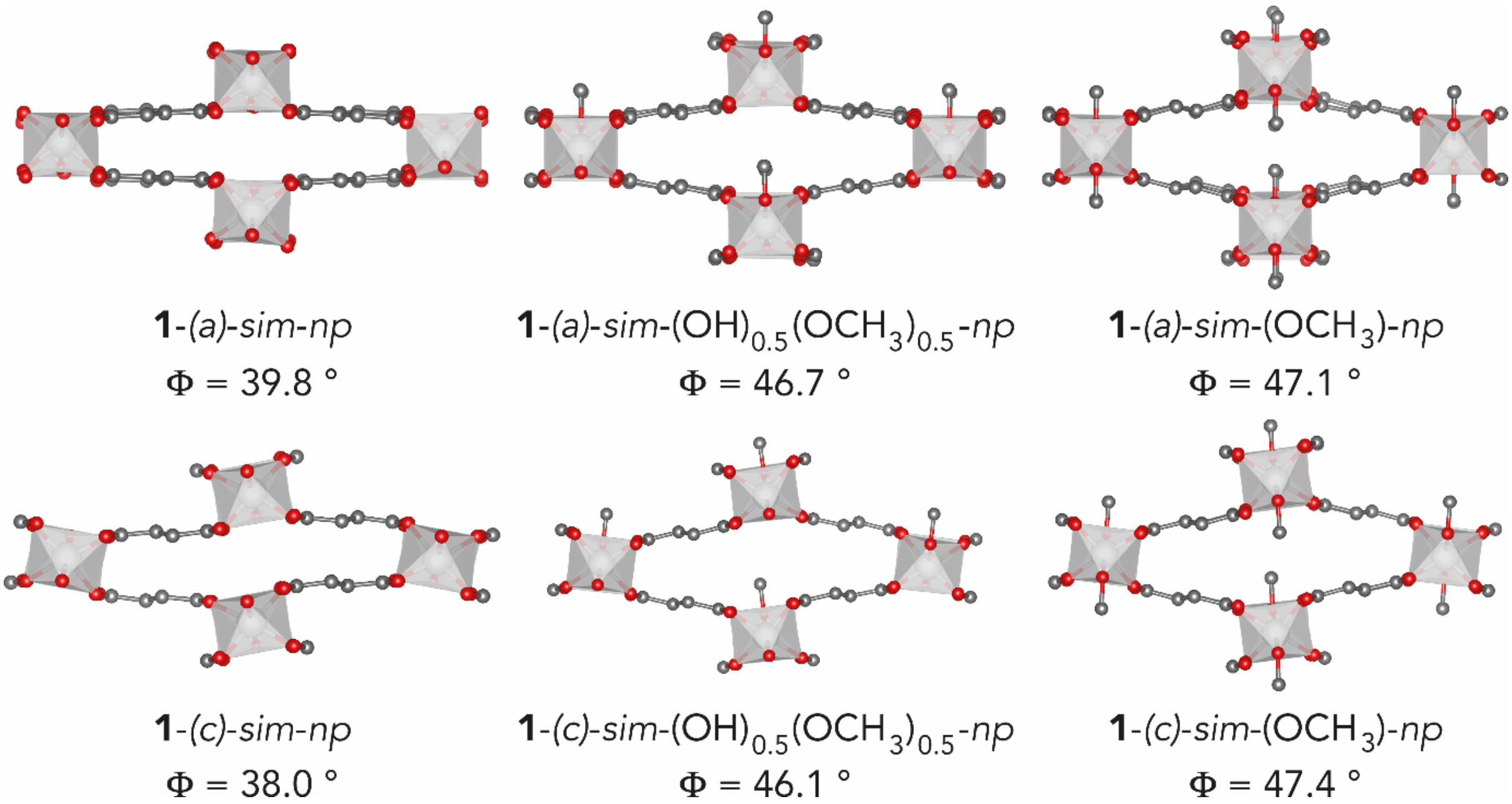

Figure S41. Comparison of the DFT simulated closed pore structures of the (*a*) and (*c*) conformational isomers with differing amounts (0%, 50%, and 100%) of μ_2-OCH_3 substitution. The extent of "open-ness" is quantified by the pore angle Φ (values are an average due to the lowered symmetry of the simulated structures. Increasing cluster anion substitution of μ_2-OH by μ_2-OCH_3 increases the Φ values.

This is evident in the predicted diffractograms for the structures (Figure S42), which show significant changes as the extent of cluster anion substitution of μ_2-OH units by μ_2-OCH_3 groups from the methanol solvent exchange.

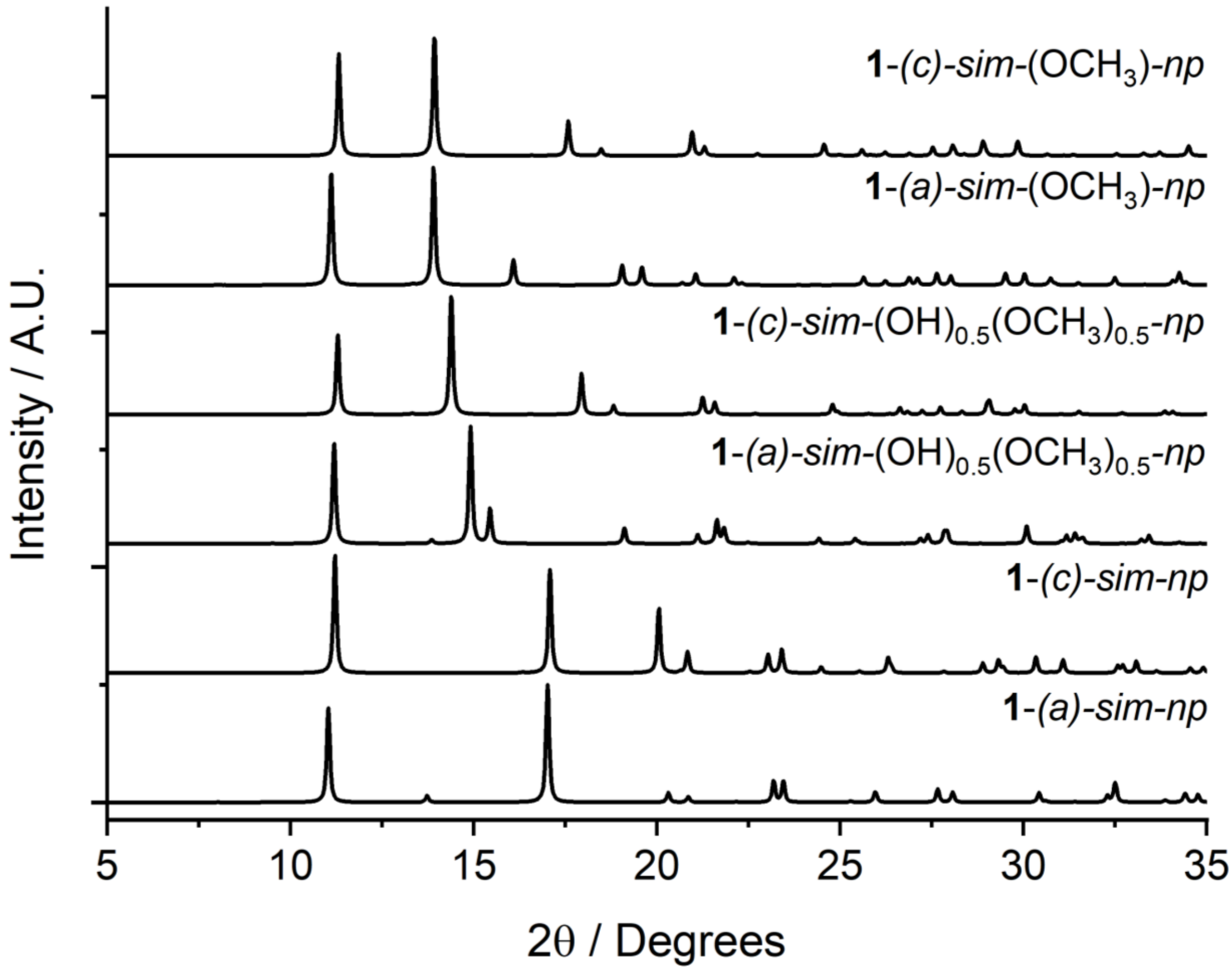

Figure S42. Stacked partial powder X-ray diffractograms predicted from the DFT models of the narrow arrow pore forms of the (*a*) and (*c*) polymorphs with different levels of cluster methoxylation: **1**-*(a)-sim-np* and **1**-*(c)-sim-np*, 0% methoxylation; **1**-*(a)-sim*-$(OH)_{0.5}(OCH_3)_{0.5}$-*np* and **1**-*(c)-sim*-$(OH)_{0.5}(OCH_3)_{0.5}$-*np*, 50% methoxylation; **1**-*(a)-sim*-(OCH_3)-*np* and **1**-*(c)-sim*-(OCH_3)-*np*, 100% methoxylation.

3.4.2. Methoxylation

As-synthesised samples were solvent exchanged with methanol by the same general procedure as for acetone. Around 50 mg of **1**-100*as*, **1**-75*as*, or **1**-50*as* was placed in a round-bottom flask and methanol was added. The mixture was refluxed overnight, without stirring, cooled to room temperature and transferred to a centrifuge tube, where it was centrifuged, and the mother solvent was decanted. The solids were collected and

dried at room temperature in a vacuum desiccator prior to analysis (as-synthesised). The materials were activated at 150 °C for 20 hours under 10^{-4} Torr vacuum (activated).

Thermogravimetric analyses of the as-synthesised samples, **1**-100*MeOH-as*, **1**-75*MeOH-as*, and **1**-50*MeOH-as*, suggested removal of all pore-bound solvents, with no notable mass loss events occurring until around 400 °C (Figure S43). Despite this, NMR spectra of acid digests (Figure S44) showed resonances attributable to methanol; the lack of solvent loss at lower temperatures in the TGA profiles would indicate the methanol is derived from degradation of OCH_3 groups attached to the MOF rather than physisorbed solvents. Integration of the resonances in the NMR spectra allowed estimation of the extent of substitution, giving values of 37%, 40%, and 38% for **1**-100*MeOH-as*, **1**-75*MeOH-as*, and **1**-50*MeOH-as,* in turn. CHN analyses were used alongside the TGA and NMR spectroscopic data to yield predicted formulae of the materials (Table S6) which matched well with the available data.

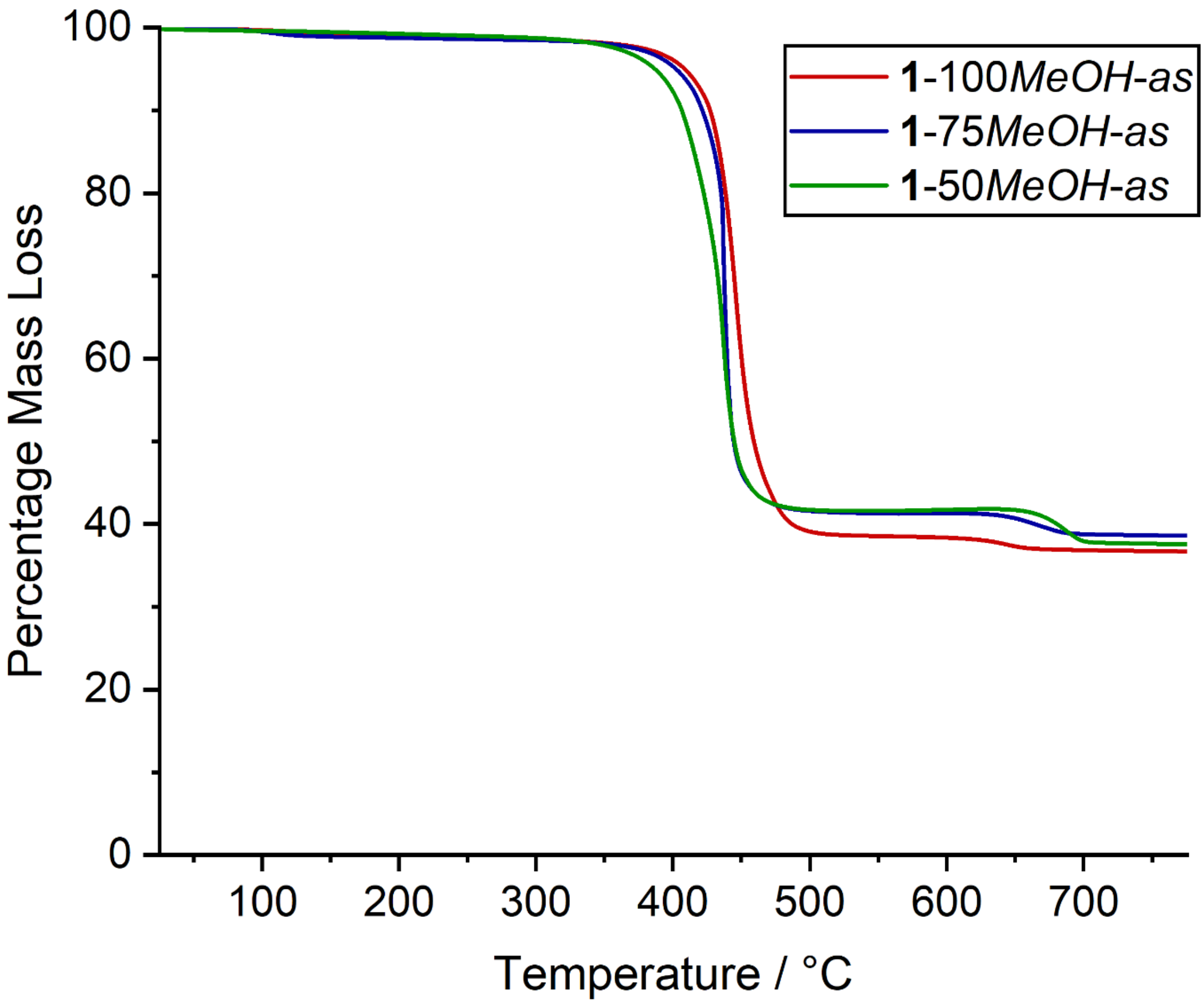

Figure S43. Thermogravimetric analysis traces of methanol exchanged samples, **1**-100*MeOH-as*, **1**-75*MeOH-as*, and **1**-50*MeOH-as*, showing no detectable solvent retention.

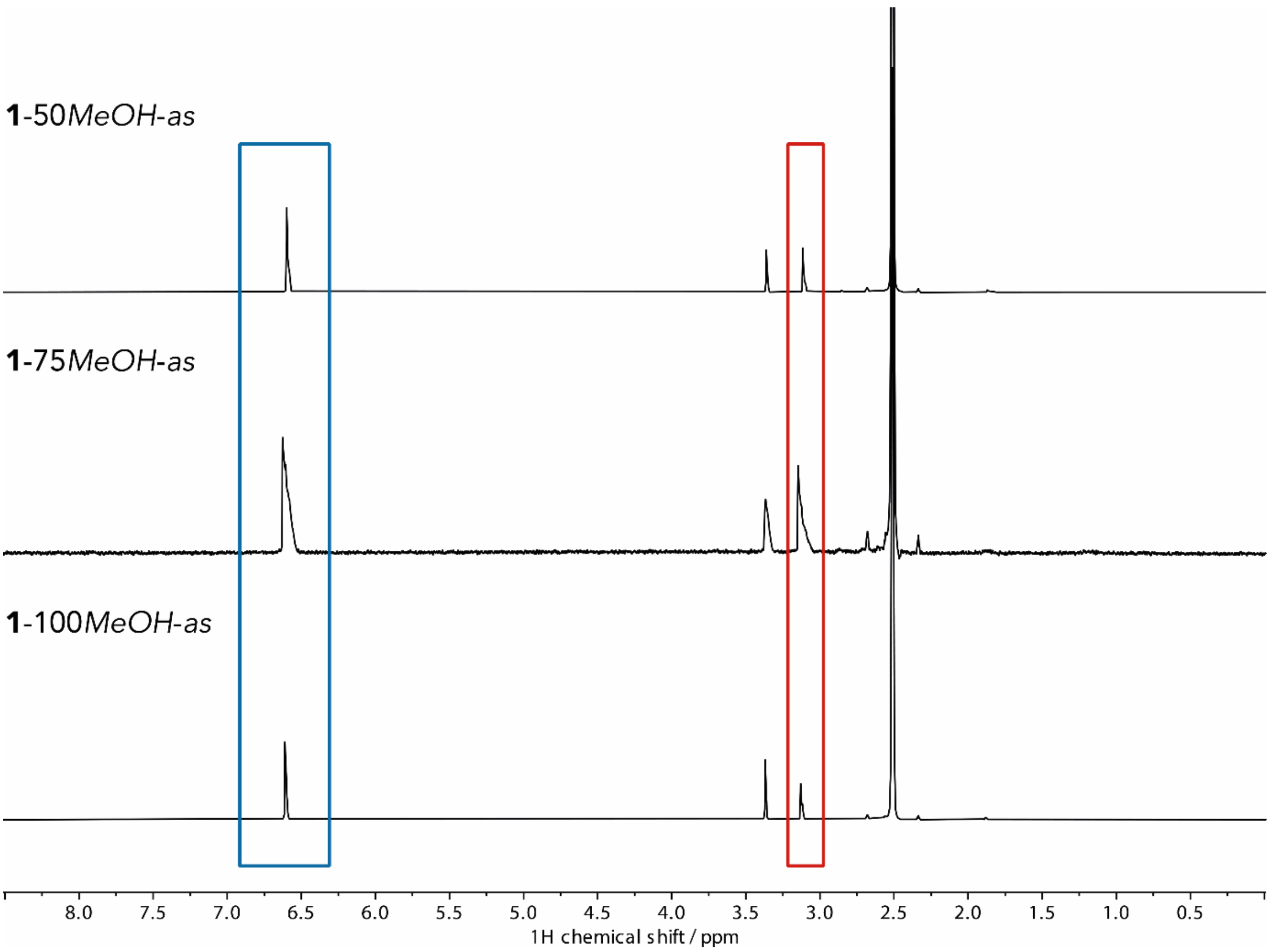

Figure S44. Stacked partial ^{1}H NMR spectra of acid-digested (DMSO-d_6/D_2SO_4) samples of methanol exchanged samples, **1**-100*MeOH-as*, **1**-75*MeOH-as*, and **1**-50*MeOH-as*. Resonances assigned to fumarate protons boxed in blue, resonances assigned to methyl protons of methanol, released by digestion of μ_2-OCH_3 units, boxed in red. Broadening of some spectra is thought to be due to unfilterable particulates.

Table S6. Table to summarise the proposed formulae for each of the samples of **1***MeOH-as* based on ^{1}H NMR spectroscopy, TGA and elemental analysis data. Data calculated from the proposed formulae are provided in brackets below the experimental values.

	Proposed Formula	Elemental Analysis			TGA Residue
		C	H	N	
**1-**100*MeOH-as*	$[Sc(OH)_{0.63}(OCH_3)_{0.37}(C_4H_2O_4)]\cdot(C_3H_7NO)_{0.02}(H_2O)_{0.1}$	30.67% (28.84%)	2.13% (2.23%)	0.12% (0.15%)	36.7% (37.3%)
**1-**75*MeOH-as*	$[Sc(OH)_{0.6}(OCH_3)_{0.4}(C_4H_2O_4)]\cdot(C_3H_7NO)_{0.02}$	29.99% (29.26%)	2.42% (2.17%)	0.18% (0.15%)	37.3% (37.6%)
**1-**50*MeOH-as*	$[Sc(OH)_{0.62}(OCH_3)_{0.38}(C_4H_2O_4)]\cdot(C_3H_7NO)_{0.05}$	29.51% (29.41%)	2.35% (2.24%)	0.37% (0.38%)	37.5% (37.3%)

The samples were activated for gas adsorption analysis by heating to 423 K under 10^{-4} Torr vacuum for 20 h to remove any residual pore-bound solvent and allow confirmation of any cluster anion substitution of OH by OCH_3.

Analysis of the activated samples by TGA (Figure S45) and NMR spectroscopy after acid digestion (Figure S46) showed little differences compared to the as-synthesised samples. The relative intensities of the integrals of the protons assigned to methanol, expected to be the decomposition product of the μ_2-OCH_3 units, decreased slightly across the samples – **1**-100: 37% to 37%; **1**-75: 40% to 38%; **1**-50: 37% to 31%. This decrease could be indicative of minor amounts of physisorbed methanol in the as-synthesised samples (although this was not observed by TGA, Figure S43) but could also stem from reversal of the cluster anion substitution process by interaction with atmospheric moisture. In any case, the presence of significant amounts of methanol in the acid digests of activated samples confirms it originates from cluster bound μ_2-OCH_3 units. The composition of the samples was assessed by a combination of the TGA and NMR spectroscopic analyses, which correlated well with elemental analyses (Table S7).

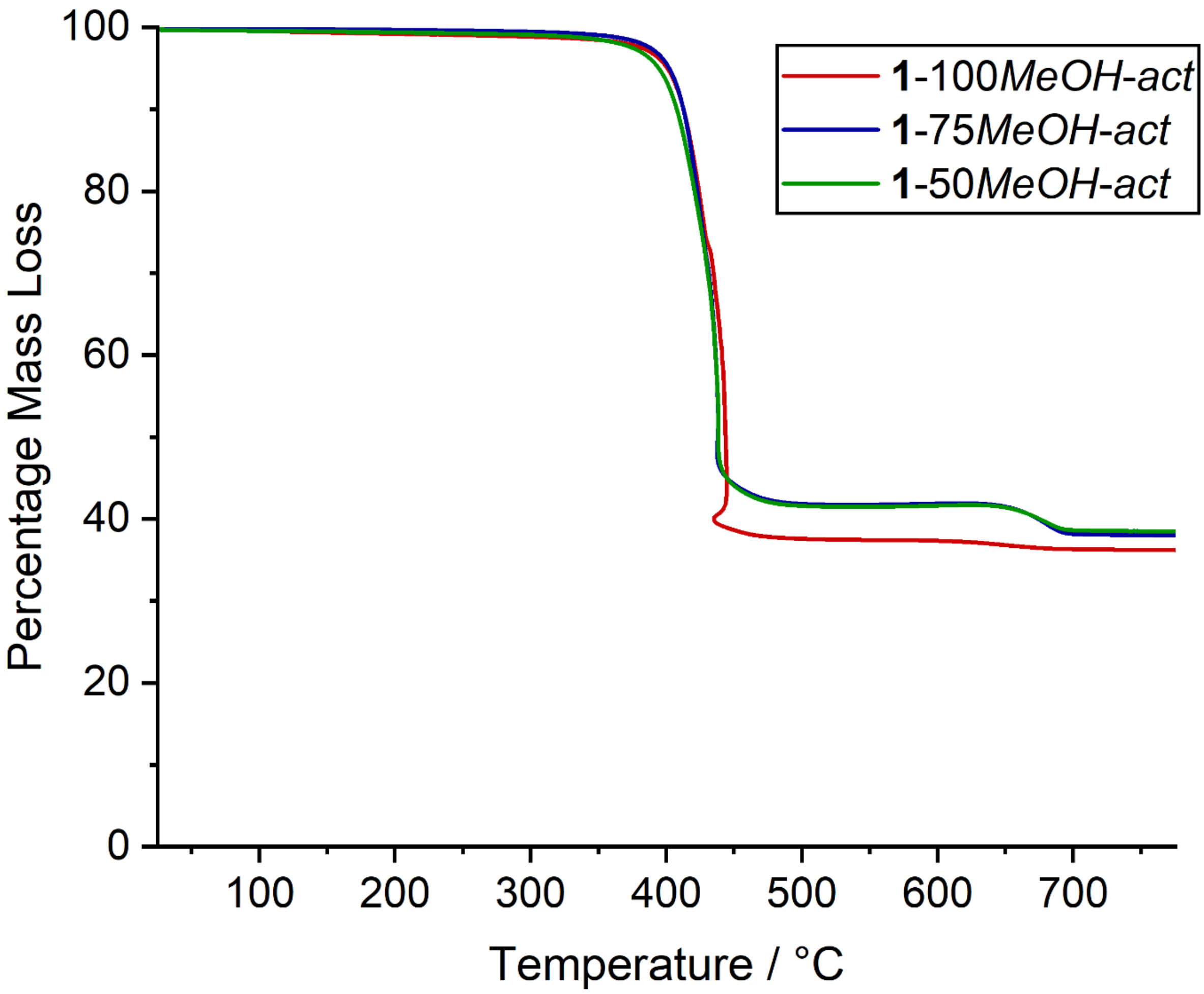

Figure S45. Thermogravimetric analysis traces of methanol exchanged and activated samples, **1**-100*MeOH-act*, **1**-75*MeOH-act*, and **1**-50*MeOH-act*, showing no detectable solvent retention in a similar manner to the as-synthesised samples in Figure S43.

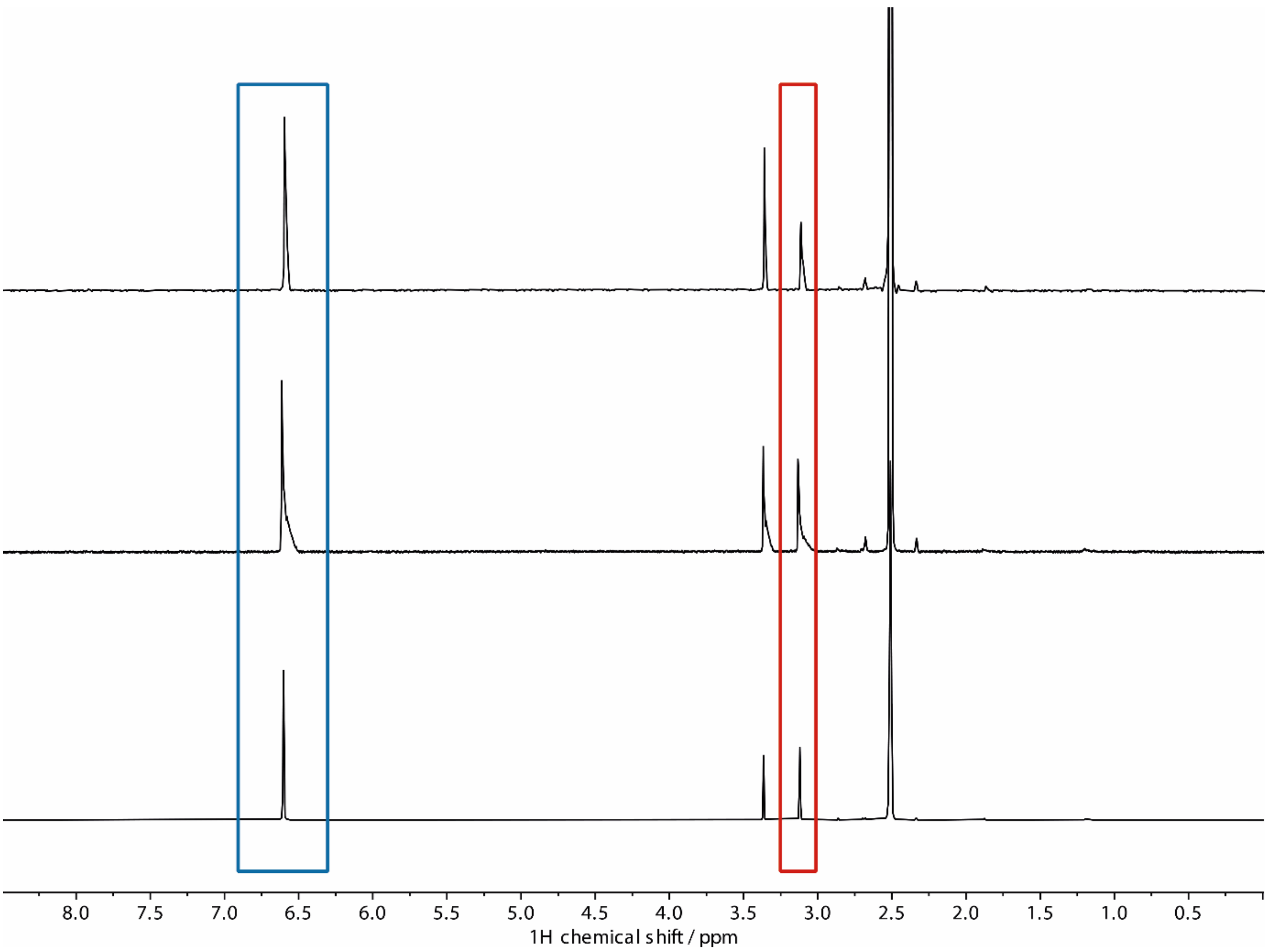

Figure S46. Stacked partial ^{1}H NMR spectra of acid-digested (DMSO-d_6/D_2SO_4) samples of methanol exchanged and activated samples, **1**-100*MeOH-act*, **1**-75*MeOH-act*, and **1**-50*MeOH-act*. Resonances assigned to fumarate protons boxed in blue, resonances assigned to methyl protons of methanol, released by digestion of μ$_2$-OCH_3 units, boxed in red. Broadening of some spectra is thought to be due to unfilterable particulates.

Table S7. Table to summarise the proposed formulae for each of the samples of **1***MeOH-act* based on ^{1}H NMR spectroscopy, TGA and elemental analysis data. Data calculated from the proposed formulae are provided in brackets below the experimental values.

	Proposed Formula	Elemental Analysis			TGA Residue
		C	H	N	
**1-**100*MeOH-act*	$[Sc(OH)_{0.63}(OCH_3)_{0.37}(C_4H_2O_4)]\cdot(C_3H_7NO)_{0.03}$	28.44% (29.12%)	1.75% (2.15%)	0.22% (0.23%)	36.7% (37.7%)
**1-**75*MeOH-act*	$[Sc(OH)_{0.62}(OCH_3)_{0.38}(C_4H_2O_4)]$	29.74% (29.01%)	2.13% (2.09%)	0.00% (0.00%)	37.3% (38.0%)
**1-**50*MeOH-act*	$[Sc(OH)_{0.69}(OCH_3)_{0.31}(C_4H_2O_4)]$	29.58% (28.70%)	2.60% (2.02%)	0.00% (0.00%)	37.5% (38.3%)

A sample of **1**-100 was solvent exchanged with methanol at room temperatureas a control experiment, and named **1**-100*MeOH-RT*. Acid-digestion NMR spectroscopy (Figure S47) showed no presence of methanol, indicative that no cluster anion substitution takes place at room temperature. No significant resonances assigned to protons of DMF were visible, suggesting complete solvent exchange but without substitution of OH by OCH_3.

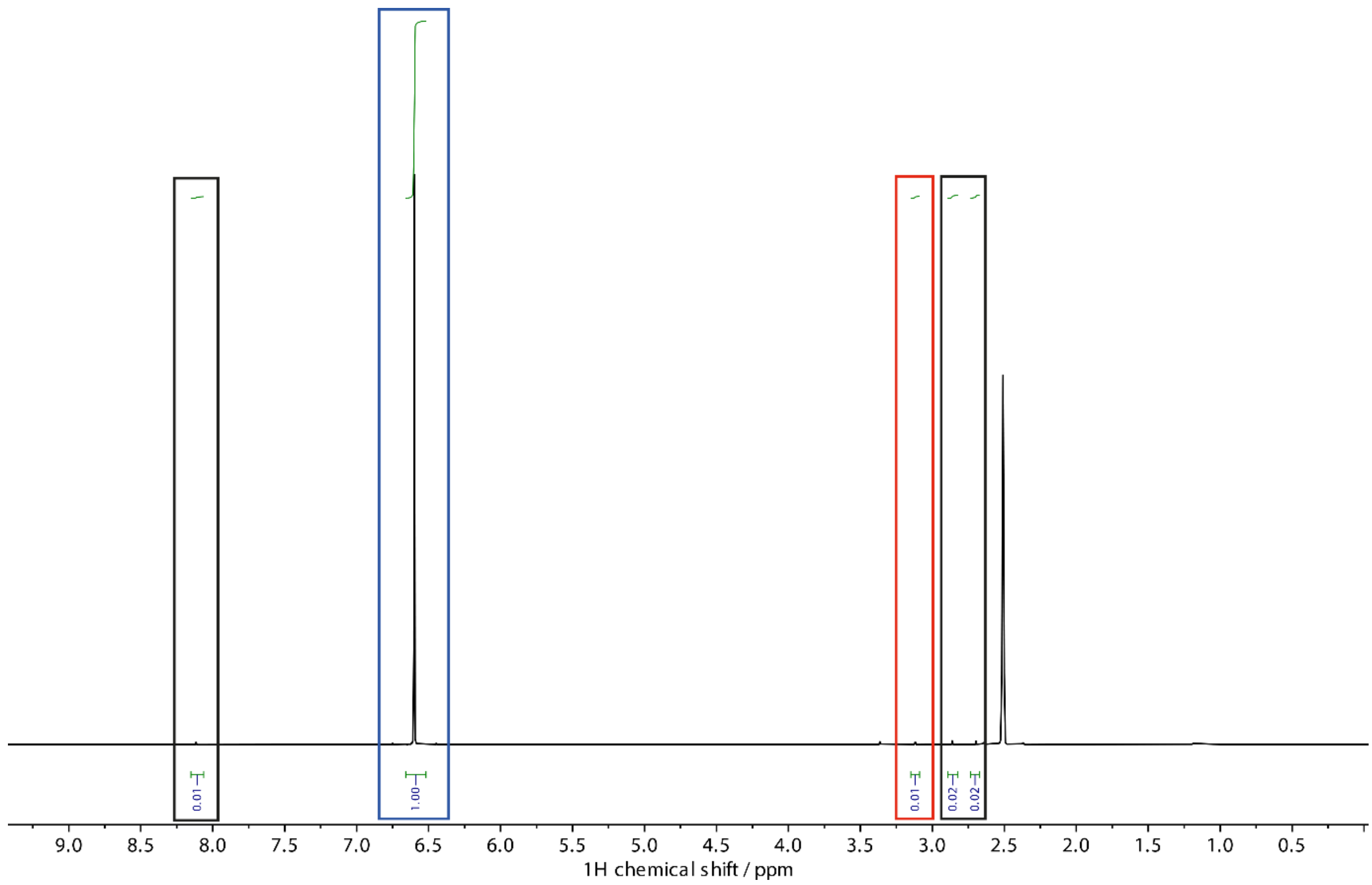

Figure S47. Partial ^{1}H NMR spectrum of acid-digested (DMSO-d_6/D_2SO_4) **1**-100*MeOH-RT-as*. Resonances assigned to fumarate protons boxed in blue, resonance tentatively assigned to trace methanol protons boxed in red, and resonances assigned to protons of residual DMF boxed in black.

A sample of **1**-75*MeOH-as* was analysed by solid-state NMR spectroscopy as described in the main manuscript (Figure 5). Additional solid-state NMR spectra collected as part of this analysis are provided in Figures S48 and S49.

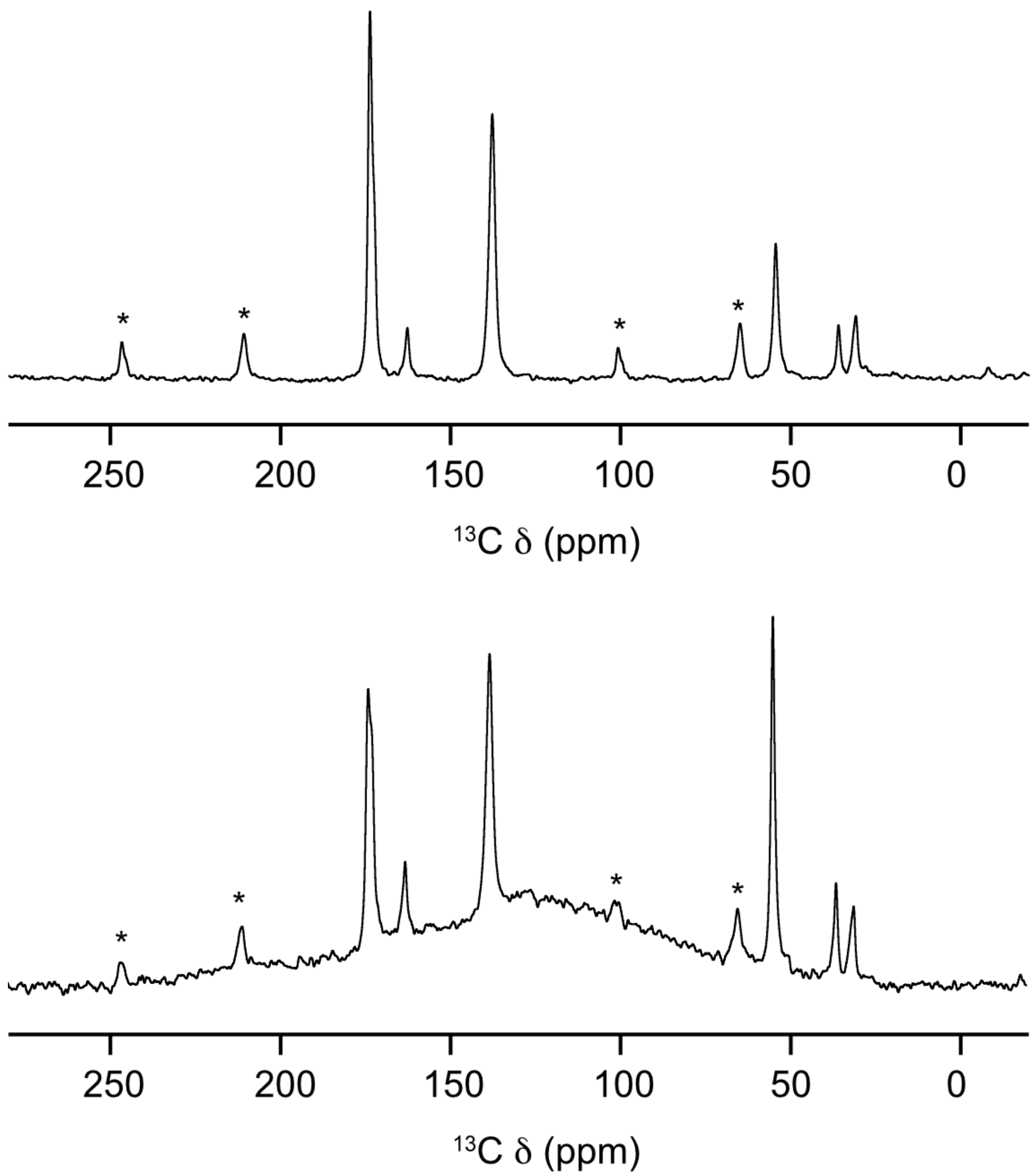

Figure S48. ^{13}C (14.1 T, 12.5 kHz) MAS NMR spectra acquired using CP (top) and direct polarisation (bottom) of **1**-75*MeOH-as* before calcination. Asterisks (*) denote spinning sidebands.

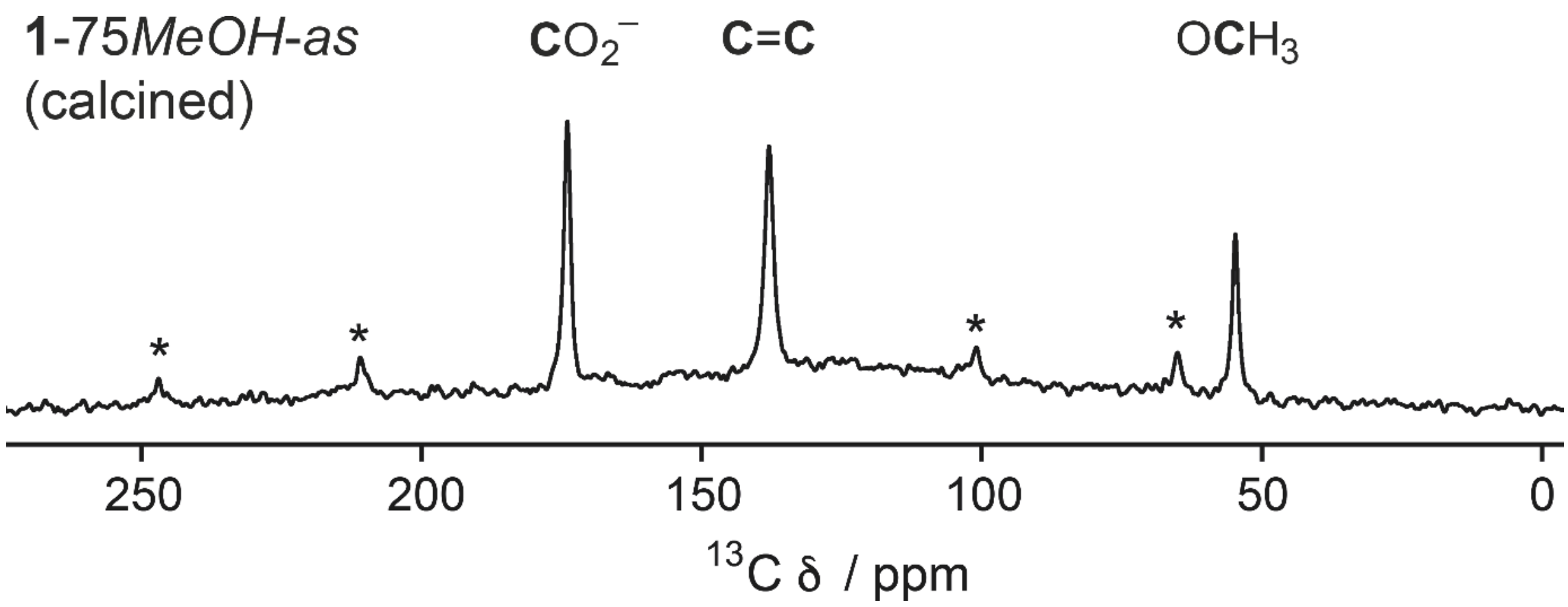

Figure S49. Direct polarisation, quantitative, ^{13}C (12.5 kHz MAS, 14.1 T) MAS NMR spectrum of **1**-75*MeOH-as* (calcined). Asterisks (*) denote spinning sidebands.

Plausible mechanisms for the methoxylation process are given in Figure S50.

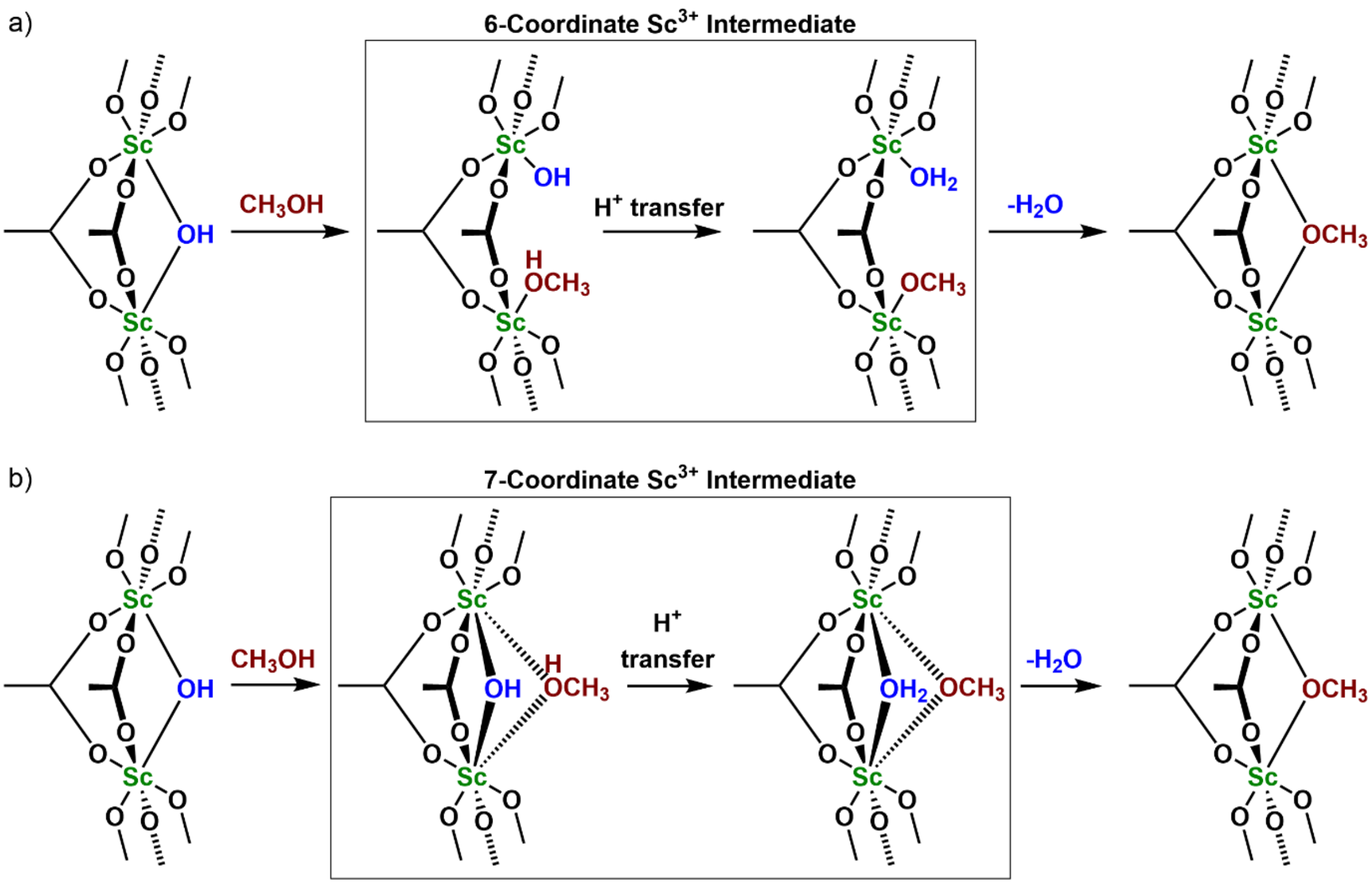

Figure S50. Two possible mechanisms for cluster anion substitution of OH units with OCH_3 through reaction with methanol at the inorganic SBU of MIL-53(Sc)-fum, proceeding through a) 6-coordinate, and b) 7-coordinate Sc^{3+} intermediates.

Powder X-ray diffraction analyses of the as-synthesised materials **1**-100*MeOH-as*, **1**-75*MeOH-as*, and **1**-50*MeOH-as* showed the samples adopt a closed pore form, even without activation, which is most reminiscent of the DFT model structure with all OH units replaced by OCH_3 (Figure S51). This aligns well with the conclusions of the solid state and acid digestion NMR spectroscopic experiments that suggest significant methoxylation. Subtle differences are seen across the samples which could be attributed to the presence of different conformational isomers, but not definitively. Interestingly, all samples gave diffractograms which correlated most closely to those predicted for the simulated models of the *(c)* conformation isomer (Figure S52).

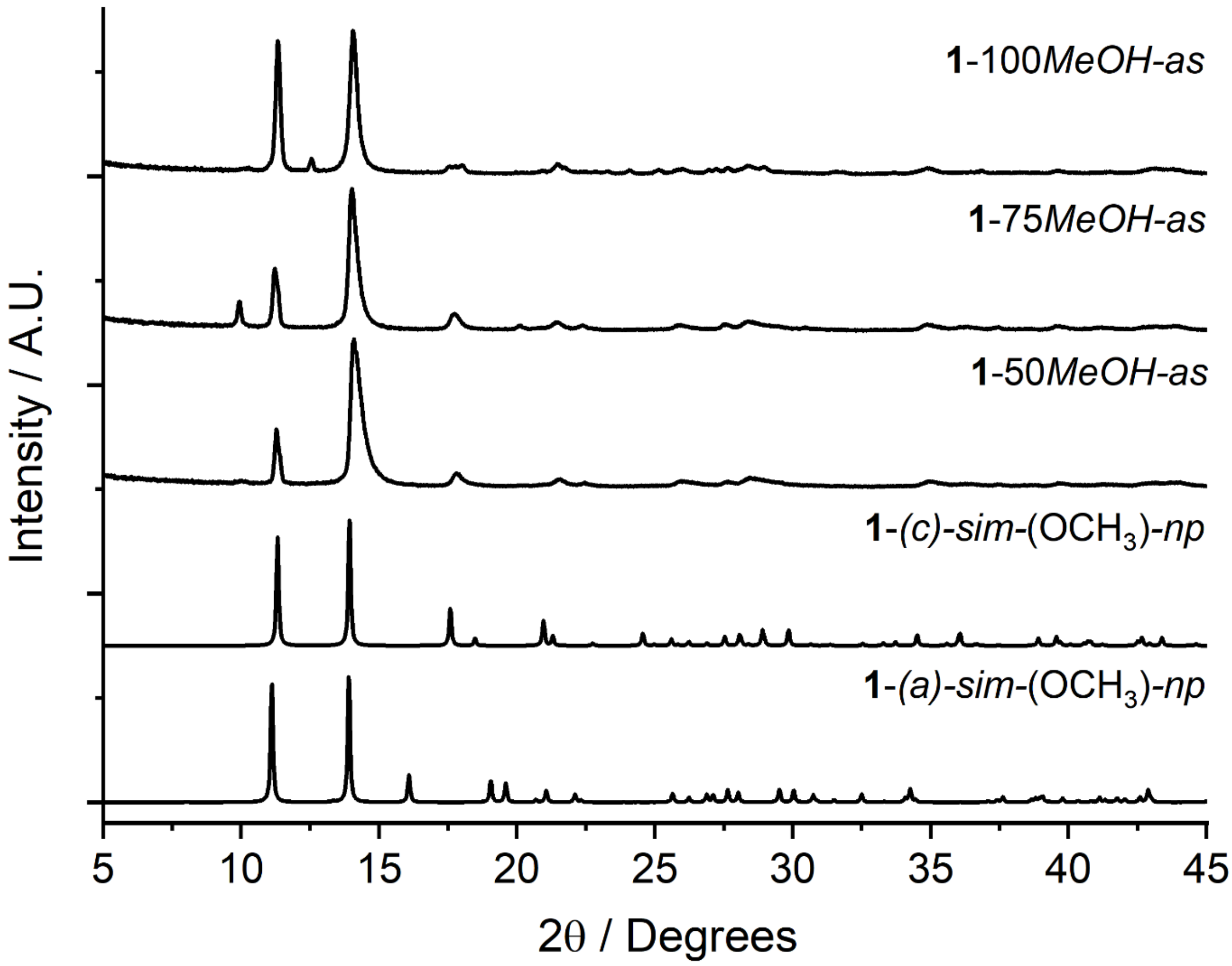

Figure S51. Stacked partial powder X-ray diffractograms of methanol exchanged samples, **1**-100*MeOH-as*, **1**-75*MeOH-as*, and **1**-50*MeOH-as* in comparison to those predicted from the DFT models of the narrow pore forms of the fully methoxylated *(a)* and *(c)* polymorphs, **1**-*(a)*-*sim*-(OCH_3)-*np* and **1**-*(c)*-*sim*-(OCH_3)-*np*.

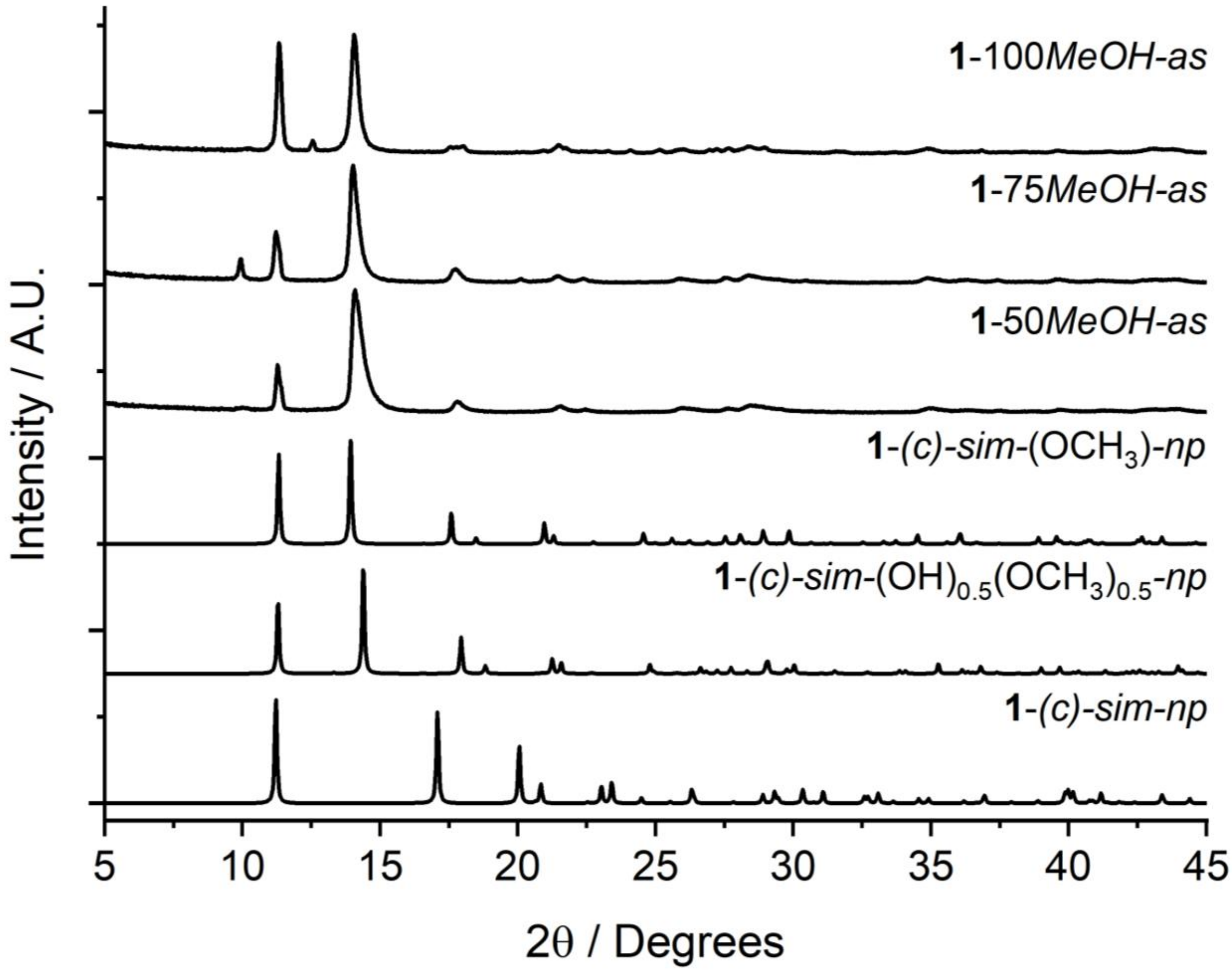

Figure S52. Stacked partial powder X-ray diffractograms of methanol exchanged samples, **1**-100*MeOH-as*, **1**-75*MeOH-as*, and **1**-50*MeOH-as* in comparison to those predicted from the DFT models of the narrow pore forms of the *(c)* polymorphs with different levels of cluster methoxylation: **1**-*(c)-sim-np*, 0% methoxylation; **1**-*(c)-sim*-$(OH)_{0.5}(OCH_3)_{0.5}$-*np*, 50% methoxylation; **1**-*(c)-sim*-(OCH_3)-*np*, 100% methoxylation.

The powder X-ray diffractograms of the activated, methanol refluxed samples (Figures S53 and S54) were very similar to the as-synthesised analogues, confirming no structural changes or formation of a more closed-pore form, which would be expected if further physisorbed solvent were removed from the pores. The diffractogram of **1**-50*MeOH-act*, as representative of the three samples, was subject to Pawley fitting (Figure S55) to give unit cell parameters indicative of a more open pore structure than the acetone-exchanged and directly activated materials.

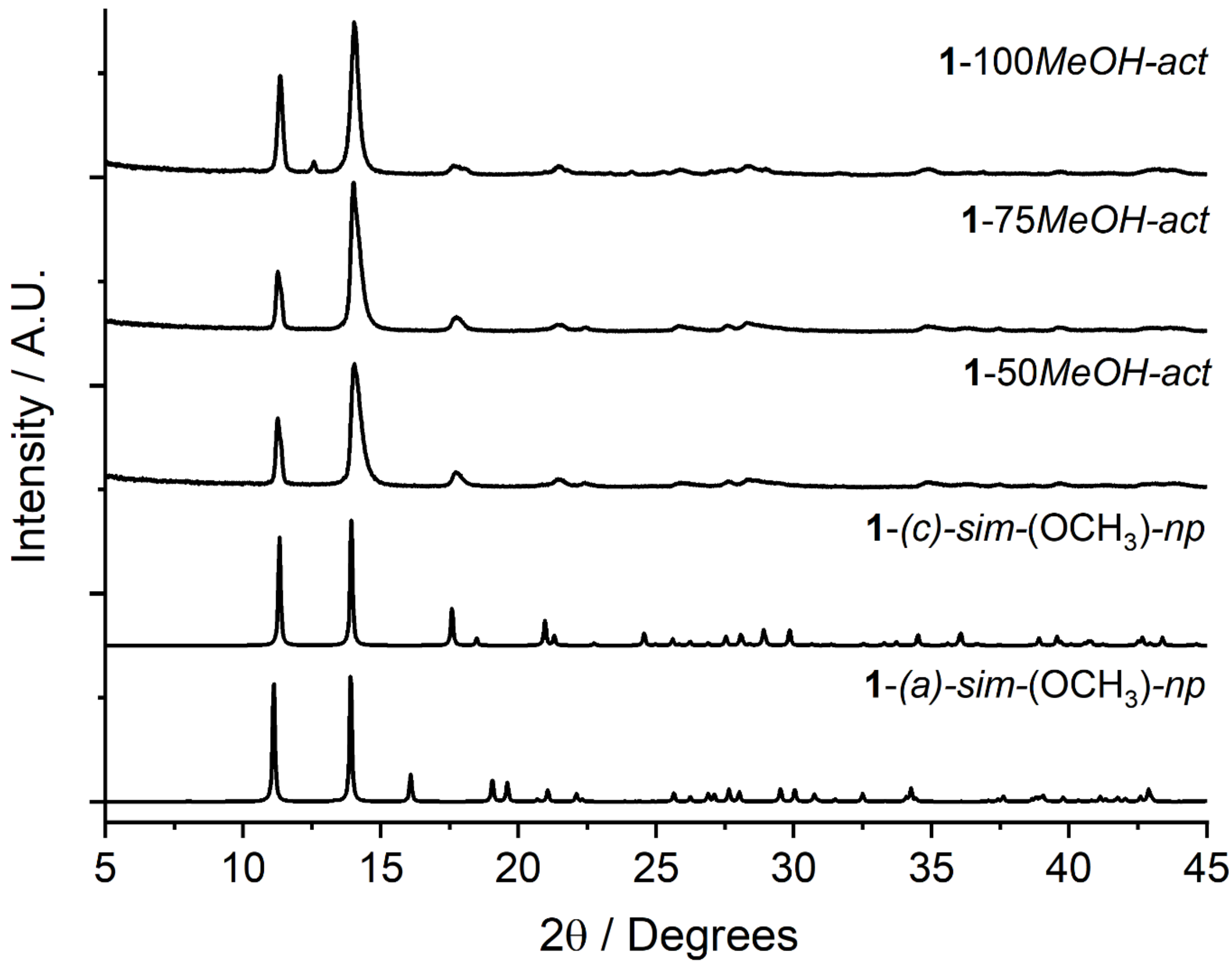

Figure S53. Stacked partial powder X-ray diffractograms of methanol exchanged and activated samples, **1**-100*MeOH-act*, **1**-75*MeOH-act*, and **1**-50*MeOH-act* in comparison to those predicted from the DFT models of the narrow pore forms of the fully methoxylated *(a)* and *(c)* polymorphs, **1**-*(a)*-*sim*-(OCH_3)-*np* and **1**-*(c)*-*sim*-(OCH_3)-*np*. Only minor changes are evident on activation, as the diffractograms closely resemble those of the as-synthesised materials (Figure S51).

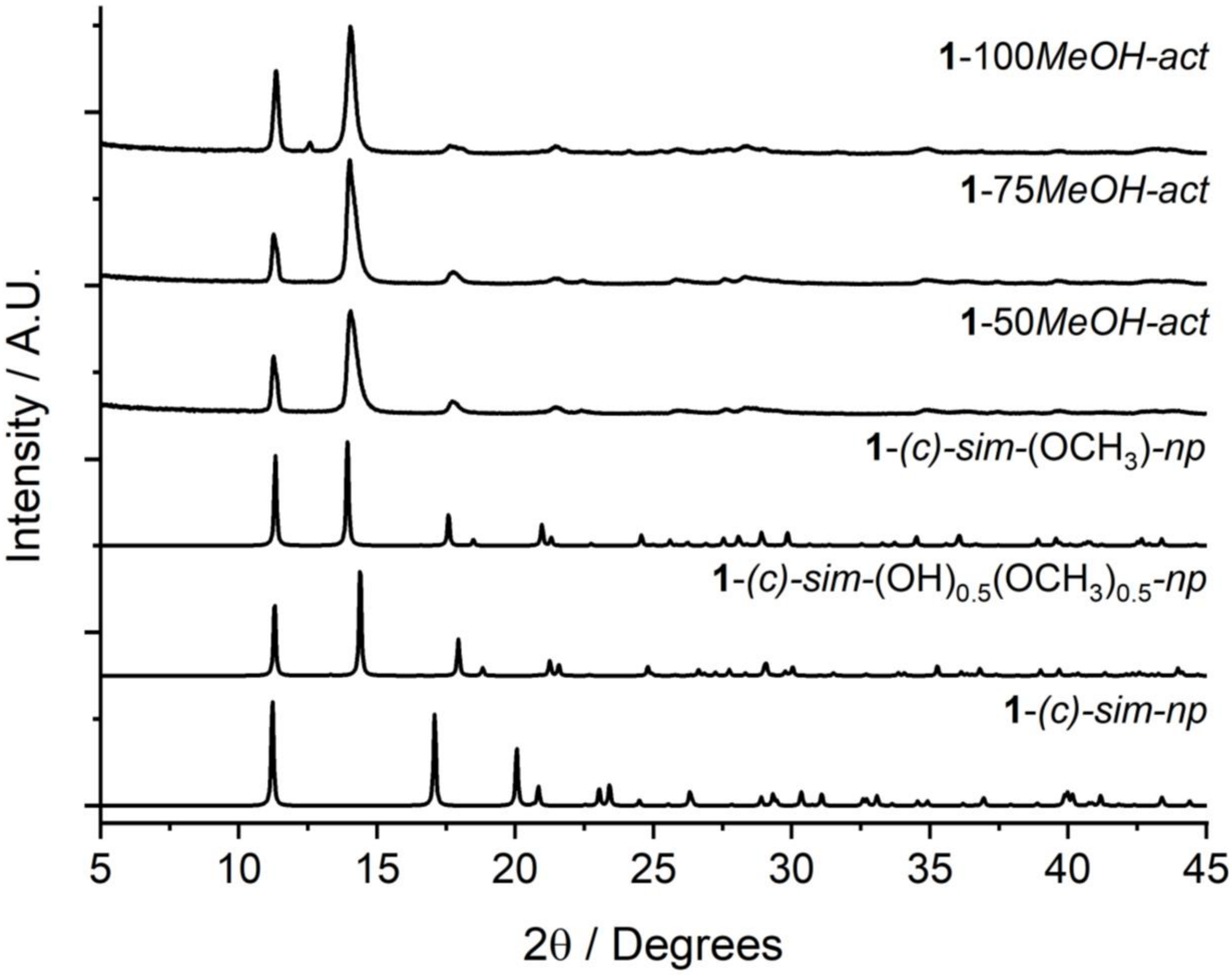

Figure S54. Stacked partial powder X-ray diffractograms of methanol exchanged and activated samples, **1**-100*MeOH-act*, **1**-75*MeOH-act*, and **1**-50*MeOH-act* in comparison to those predicted from the DFT models of the narrow pore forms of the *(c)* polymorphs with different levels of cluster methoxylation: **1**-*(c)*-*sim*-*np*, 0% methoxylation; **1**-*(c)*-*sim*-$(OH)_{0.5}(OCH_3)_{0.5}$-*np*, 50% methoxylation; **1**-*(c)*-*sim*-(OCH_3)-*np*, 100% methoxylation.

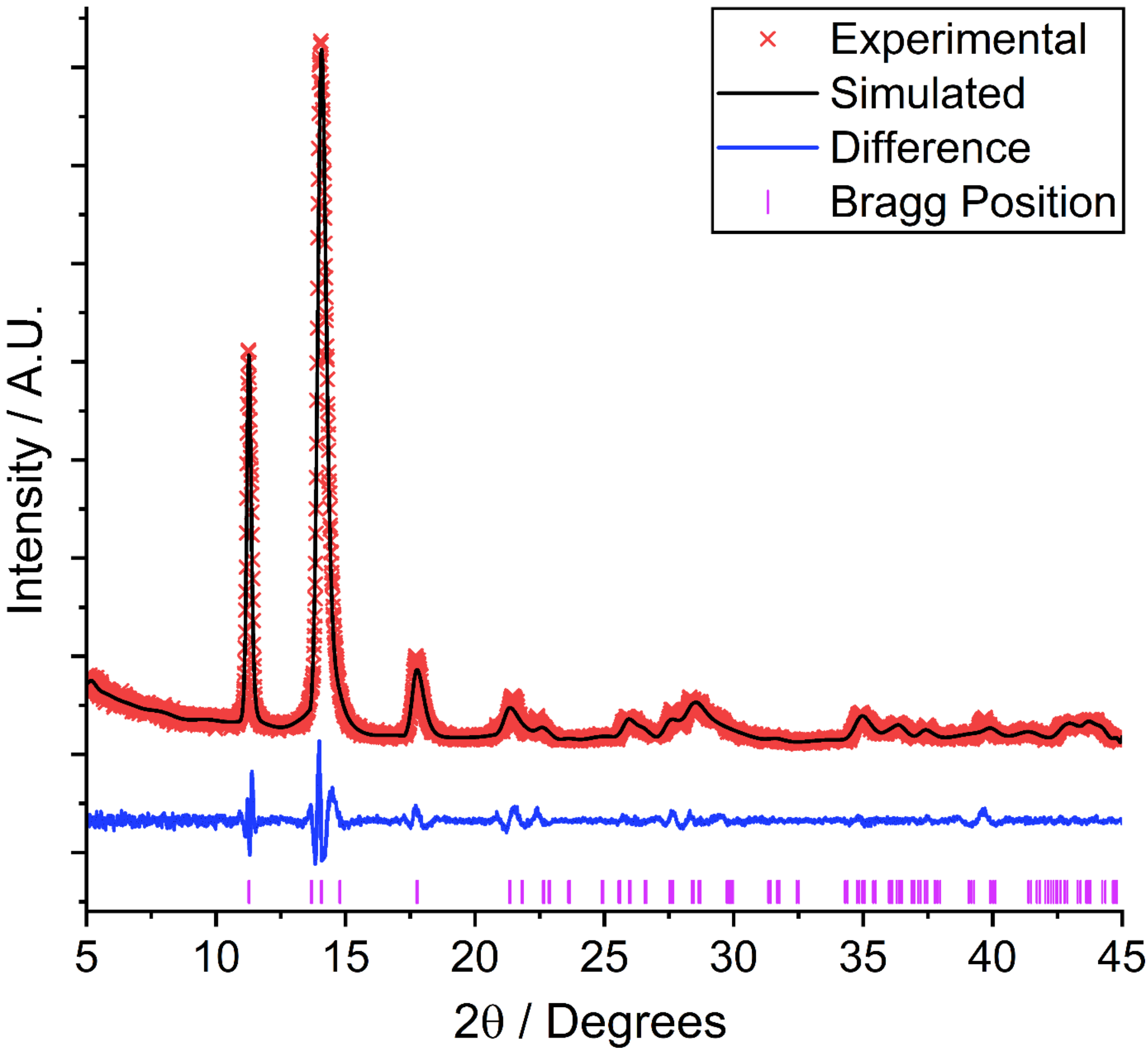

Figure S55. Pawley fitting of the powder X-ray diffractogram of **1**-50*MeOH-act* as a representative fit for all methoxylated samples. Parameters: a = 7.473 Å, b = 15.986 Å, c = 7.916 Å; β = 118.02 °; V = 835 Å^3; R_p = 7.65%, R_{wp} = 9.65%.

Comparison of the diffractograms of all activated samples (Figure S56) clearly shows the significant structural differences brought about by the cluster anion substitution process.

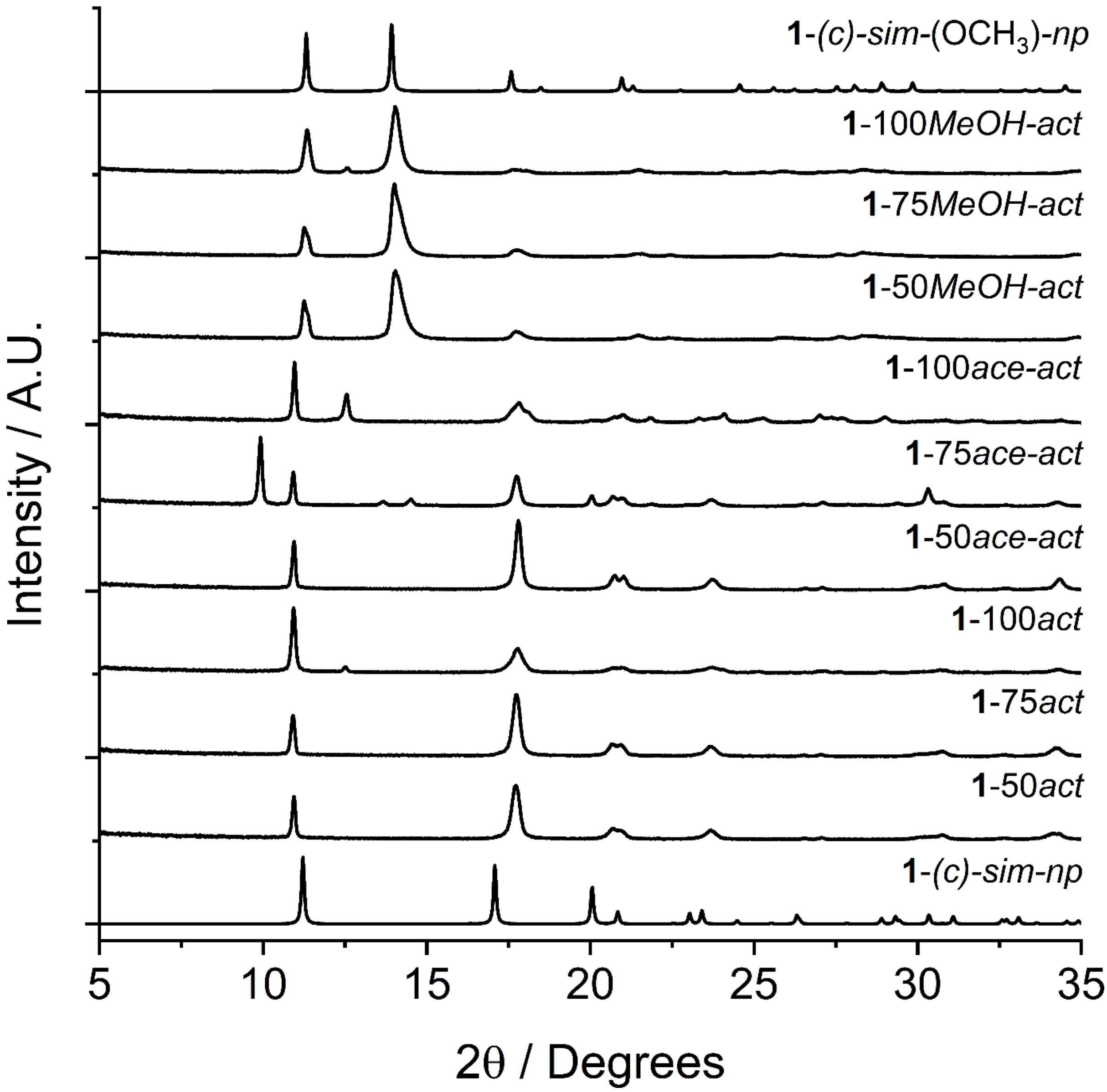

Figure S56. Stacked partial powder X-ray diffractograms of directly activated, acetone exchanged and activated, and methanol exchanged and activated samples in comparison to those predicted from the DFT models of the narrow pore forms of the *(c)* polymorph, **1**-*(c)-sim-np*, and the DFT model of the narrow pore form of the 100% methoxylated *(c)* polymorph, **1**-*(c)-sim*-(OCH_3)-*np*.

N_2 adsorption/desorption isotherms (77 K) were collected on the samples and reported in Figure 6c of the main manuscript. BET areas for the samples were determined using the BETSI software package[S3], the outputs of which are given in Figures S57-S62.

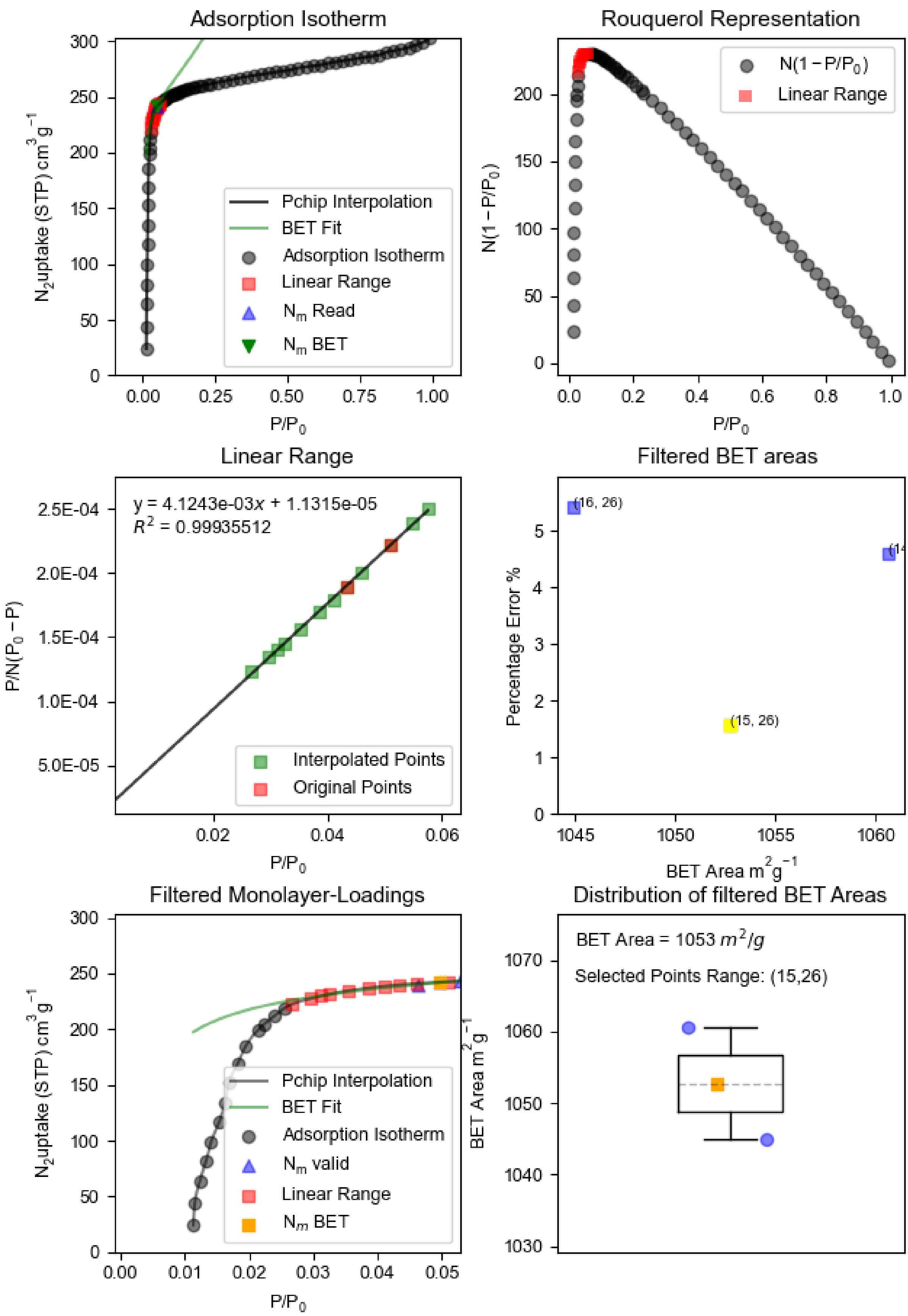

Figure S57. BETSI[S3] analysis for the N_2 adsorption isotherm (77 K) of **1**-100*MeOH-act*.

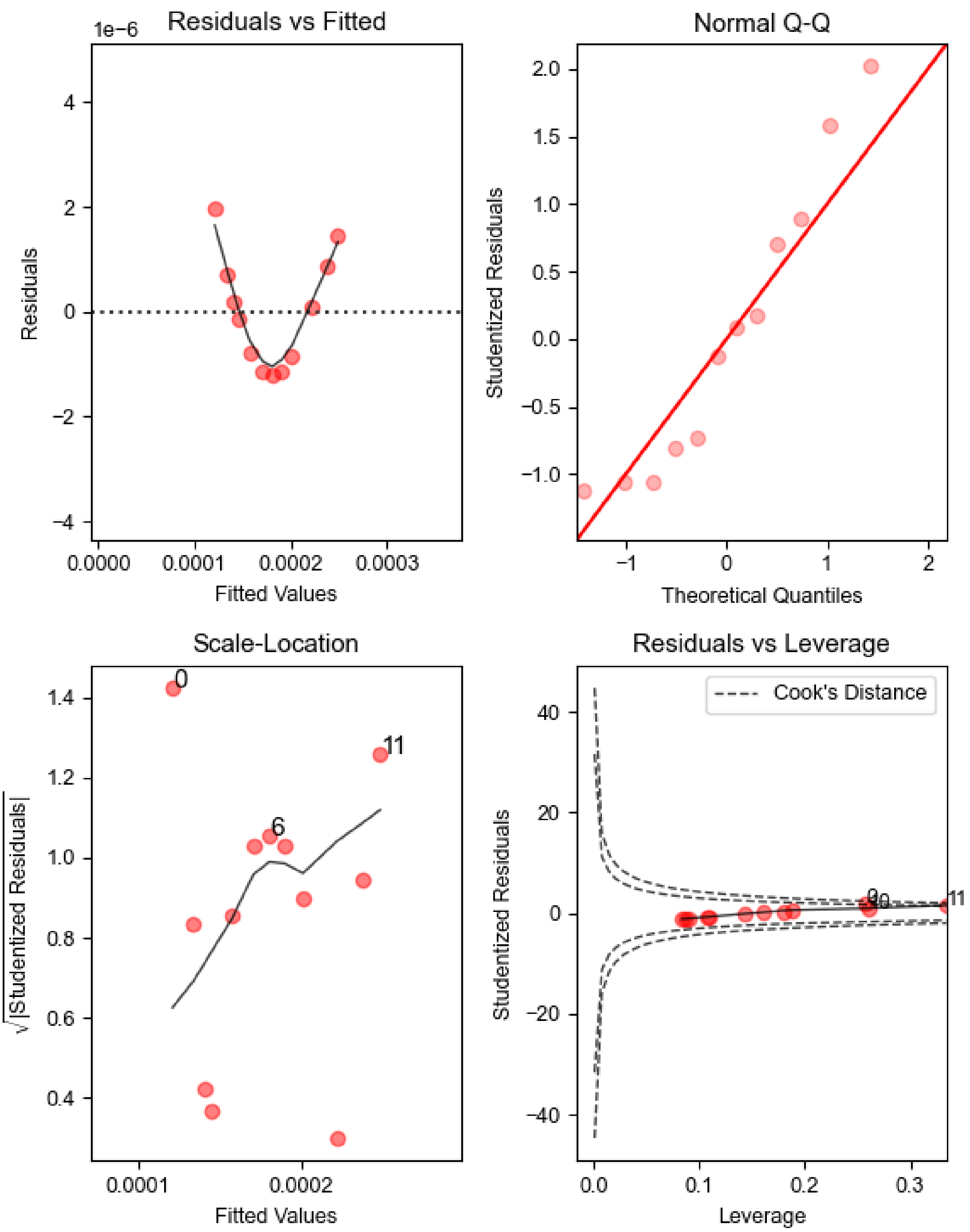

Figure S58. BETSI[S3] regression diagnostics for the N_2 adsorption isotherm (77 K) of **1**-100*MeOH-act*.

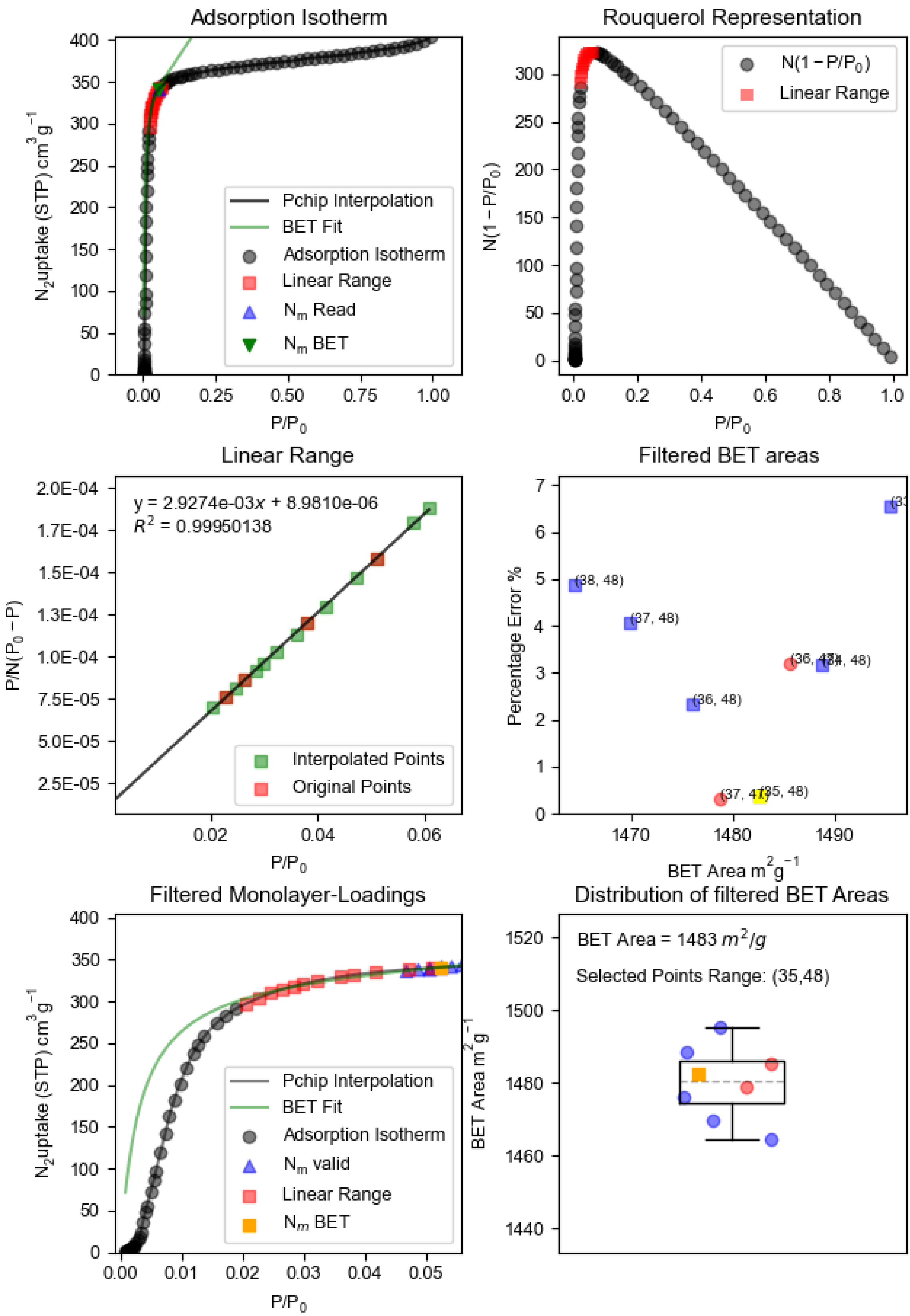

Figure S59. BETSI[S3] analysis for the N_2 adsorption isotherm (77 K) of **1**-75*MeOH-act*.

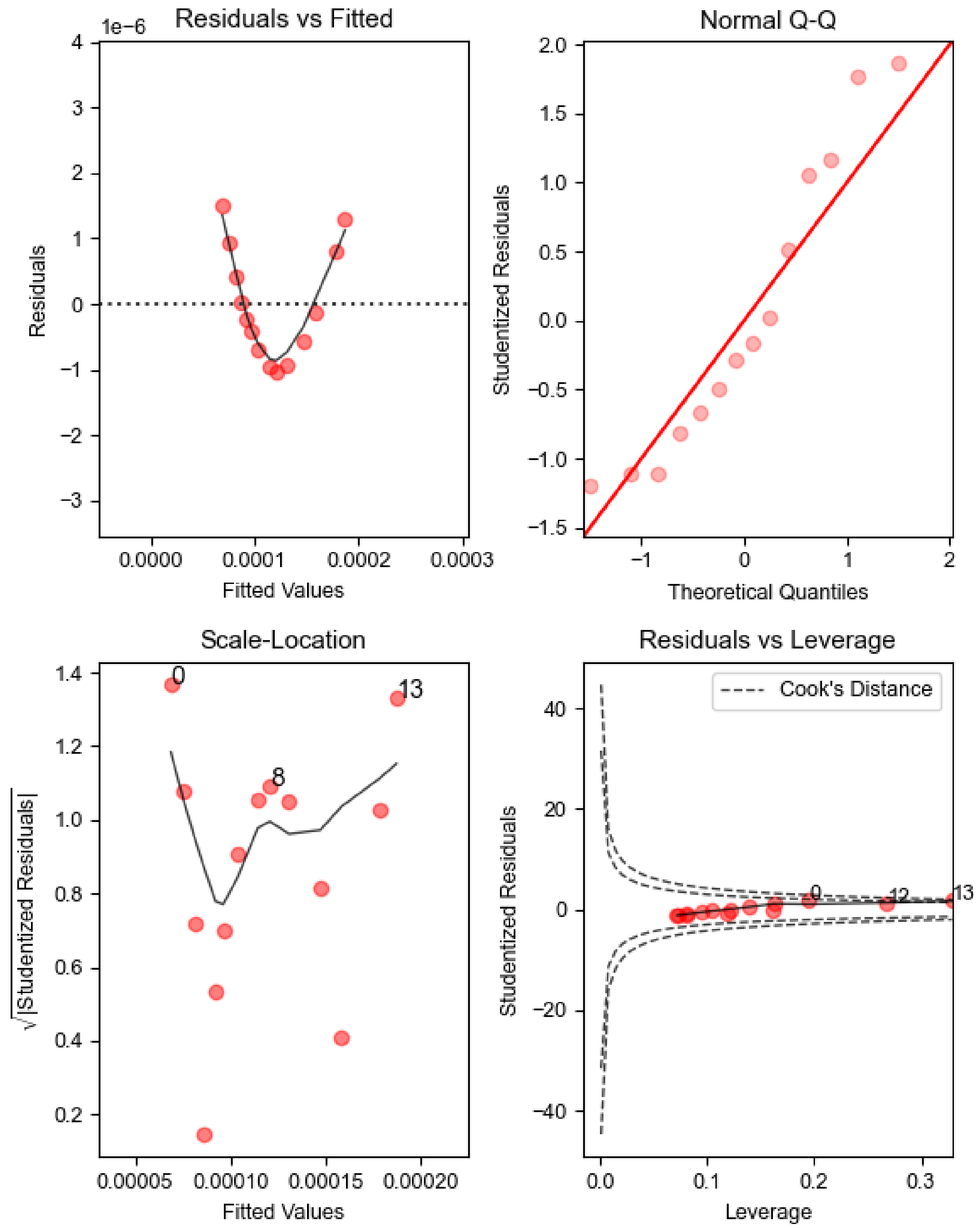

Figure S60. BETSI[S3] regression diagnostics for the N_2 adsorption isotherm (77 K) of **1**-75*MeOH-act*.

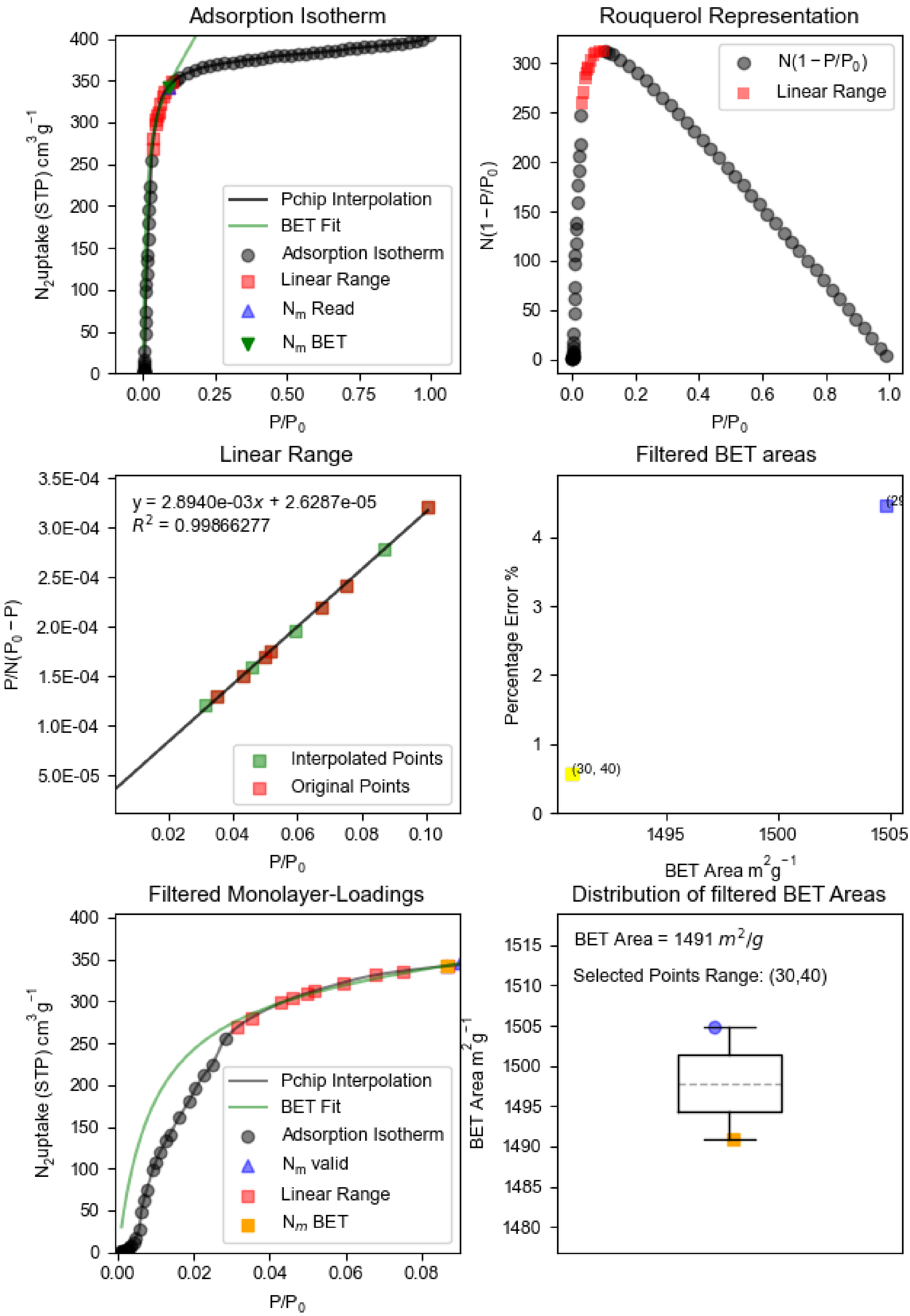

Figure S61. BETSI[S3] analysis for the N_2 adsorption isotherm (77 K) of **1**-50*MeOH-act*.

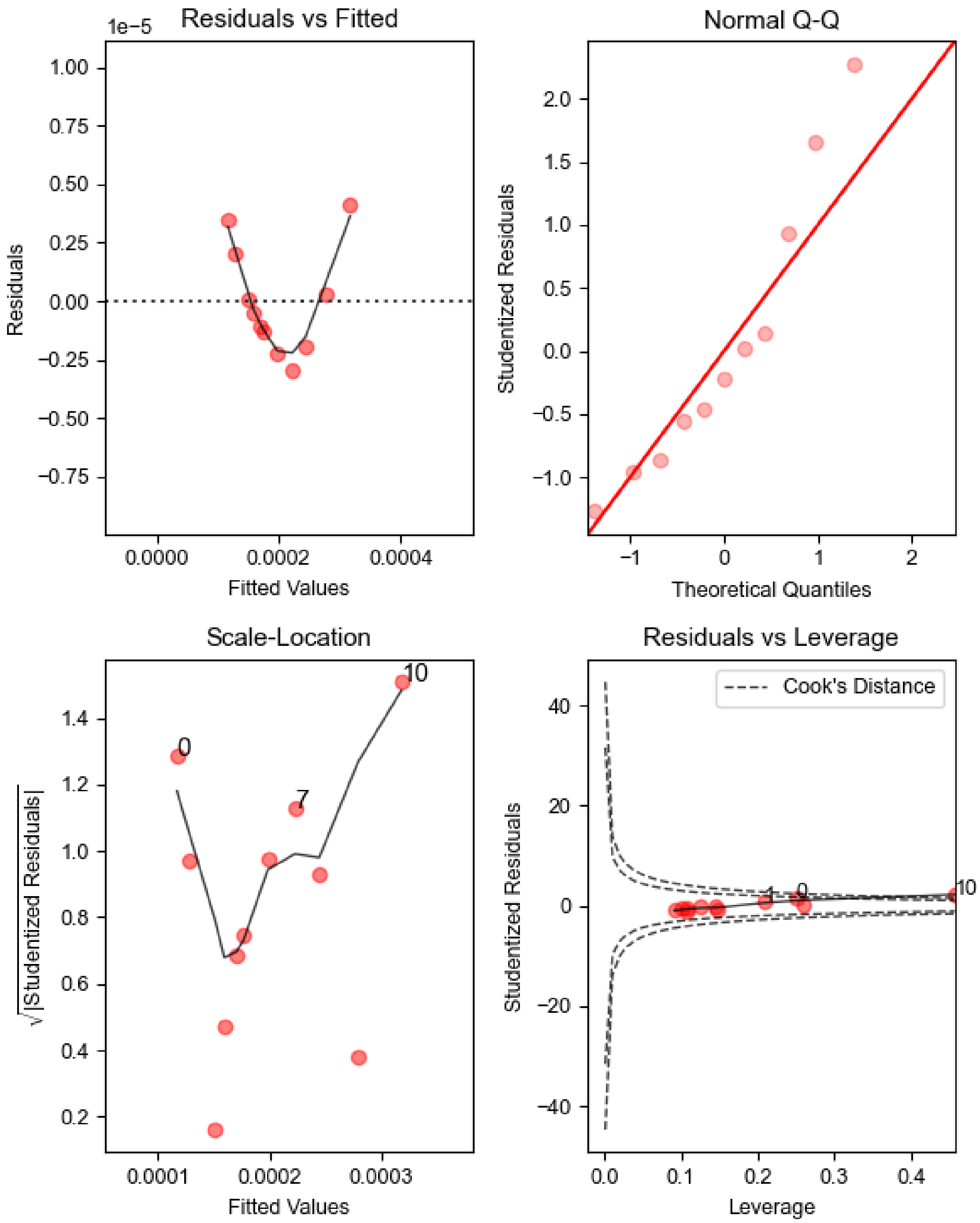

Figure S62. BETSI[S3] regression diagnostics for the N_2 adsorption isotherm (77 K) of **1**-50*MeOH-act*.

S4. References

(S1) Coles, S. J.; Gale, P. A. Changing and challenging times for service crystallography. *Chem. Sci.* **2012**, *3* (3), 683–689, 10.1039/C2SC00955B.

(2) Sheldrick, G. SHELXT - Integrated space-group and crystal-structure determination. *Acta Crystallographica Section A* **2015**, *71* (1), 3–8.

(S3) Osterrieth, J. W. M.; Rampersad, J.; Madden, D.; Rampal, N.; Skoric, L.; Connolly, B.; Allendorf, M. D.; Stavila, V.; Snider, J. L.; Ameloot, R.; et al. How Reproducible are Surface Areas Calculated from the BET Equation? *Adv. Mater.* **2022**, *34* (27), 2201502, 10.1002/adma.202201502.

(S4) Pines, A.; Gibby, M. G.; Waugh, J. S. Proton-Enhanced Nuclear Induction Spectroscopy. A Method for High Resolution NMR of Dilute Spins in Solids. *J. Chem. Phys.* **1972**, *56* (4), 1776–1777, 10.1063/1.1677439.

(S5) Bennett, A. E.; Rienstra, C. M.; Auger, M.; Lakshmi, K. V.; Griffin, R. G. Heteronuclear decoupling in rotating solids. *J. Chem. Phys.* **1995**, *103* (16), 6951–6958, 10.1063/1.470372.

(S6) a) VandeVondele, J.; Krack, M.; Mohamed, F.; Parrinello, M.; Chassaing, T.; Hutter, J. Quickstep: Fast and accurate density functional calculations using a mixed Gaussian and plane waves approach. *Comput. Phys. Commun.* **2005**, *167* (2), 103–128, 10.1016/j.cpc.2004.12.014; b) Hutter, J.; Iannuzzi, M.; Schiffmann, F.; VandeVondele, J. cp2k: atomistic simulations of condensed matter systems. *Wiley Interdiscip. Rev. Comput. Mol. Sci.* **2014**, *4* (1), 15–25, 10.1002/wcms.1159.

(S7) VandeVondele, J.; Hutter, J. Gaussian basis sets for accurate calculations on molecular systems in gas and condensed phases. *J. Chem. Phys.* **2007**, *127* (11), 114105, 10.1063/1.2770708.

(S8) a) Goedecker, S.; Teter, M.; Hutter, J. Separable dual-space Gaussian pseudopotentials. *Phys. Rev.B* **1996**, *54* (3), 1703–1710, 10.1103/PhysRevB.54.1703; b) Krack, M. Pseudopotentials for H to Kr optimized for gradient-corrected exchange-correlation functionals. *Theor. Chem. Acc.* **2005**, *114* (1), 145–152, 10.1007/s00214-005-0655-y.

(S9) Perdew, J. P.; Burke, K.; Ernzerhof, M. Generalized Gradient Approximation Made Simple. *Phys. Rev.Lett.* **1996**, *77* (18), 3865–3868, 10.1103/PhysRevLett.77.3865.
(S10) Grimme, S.; Antony, J.; Ehrlich, S.; Krieg, H. A consistent and accurate ab initio parametrization of density functional dispersion correction (DFT-D) for the 94 elements H-Pu. *J. Chem. Phys.* **2010**, *132* (15), 154104, 10.1063/1.3382344.
(S11) Walshe, C. A.; Thom, A. J. R.; Wilson, C.; Ling, S.; Forgan, R. S. Controlling the Flexibility of MIL-88A(Sc) Through Synthetic Optimisation and Postsynthetic Halogenation. *Chem. Eur. J.* **2022**, *28* (48), e202201364, 10.1002/chem.202201364.
(S12) Hong, C. C.; Leung, W. L. W.; Wilson, C.; Liddle, M. J.; Forgan, R. S.; Levinsky, J. J. B.; Mey, A. S. J. S.; Hobday, C. L. Conformational Isomerism Tunes Barocaloric Potential of MIL-53(Ga)-fum Frameworks. *J. Mater. Chem. A* **2026**, 10.1039/D1035TA08920D.
(S13) Ehrling, S.; Reynolds, E. M.; Bon, V.; Senkovska, I.; Gorelik, T. E.; Evans, J. D.; Rauche, M.; Mendt, M.; Weiss, M. S.; Pöppl, A.; et al. Adaptive response of a metal–organic framework through reversible disorder–disorder transitions. *Nature Chem.* **2021**, *13* (6), 568–574. DOI: 10.1038/s41557-021-00684-4.
(S14) Serre, C.; Millange, F.; Thouvenot, C.; Noguès, M.; Marsolier, G.; Louër, D.; Férey, G. Very Large Breathing Effect in the First Nanoporous Chromium(III)-Based Solids: MIL-53 or CrIII(OH)·$\{O_2C-C_6H_4-CO_2\}\cdot\{HO_2C-C_6H_4-CO_2H\}_x\cdot H_2O_y$. *J. Am. Chem. Soc.* **2002**, *124* (45), 13519–13526, 10.1021/ja0276974.
(S15) Alvarez, E.; Guillou, N.; Martineau, C.; Bueken, B.; Van de Voorde, B.; Le Guillouzer, C.; Fabry, P.; Nouar, F.; Taulelle, F.; de Vos, D.; et al. The Structure of the Aluminum Fumarate Metal–Organic Framework A520. *Angew. Chem. Int. Ed.* **2015**, *54* (12), 3664–3668,10.1002/anie.201410459.
(S16) Ramaswamy, P.; Wieme, J.; Alvarez, E.; Vanduyfhuys, L.; Itié, J.-P.; Fabry, P.; Van Speybroeck, V.; Serre, C.; Yot, P. G.; Maurin, G. Mechanical properties of a gallium fumarate metal–organic framework: a joint experimental-modelling exploration. *J. Mater. Chem. A* **2017**, *5* (22), 11047–11054, 10.1039/C7TA01559C.
(S17) Zhang, Y.; Lucier, B. E. G.; McKenzie, S. M.; Arhangelskis, M.; Morris, A. J.; Friščić, T.; Reid, J. W.; Terskikh, V. V.; Chen, M.; Huang, Y. Welcoming Gallium- and Indium-Fumarate MOFs to the Family: Synthesis, Comprehensive Characterization,

Observation of Porous Hydrophobicity, and CO_2 Dynamics. *ACS Appl. Mater. Interfaces* **2018**, *10* (34), 28582–28596, 10.1021/acsami.8b08562.
